# The SHEF1552 dataset: fundamental trends and physical insights into halogen, chalcogen, pnictogen, and tetrel bonds

**DOI:** 10.1039/d6cp04299f

**Published:** 2026-11-06

**Authors:** Harry W. Nash, Lee Brammer, J. Grant Hill

**Affiliations:** a Chemistry Division, School of Mathematical and Physical Sciences, University of Sheffield Sheffield S3 7HF UK; b Centre for Machine Intelligence, University of Sheffield Sheffield S10 2TN UK grant.hill@sheffield.ac.uk

## Abstract

The Sigma-Hole Energies and additional Factors (SHEF1552) dataset, a comprehensive collection of 1552 σ-hole interactions (halogen, chalcogen, pnictogen, and tetrel bonds) is introduced. Each complex, formed by pairing 82 σ-hole donors with 19 Lewis bases, was analysed using explicitly correlated coupled-cluster interaction energies and symmetry-adapted perturbation theory (SAPT). Analysis of this dataset identifies key geometric and energetic trends. Pnictogen bonds are, on average, the strongest interactions, followed by tetrel, chalcogen, and halogen bonds. The interaction energy-distance relationship exhibits a distinct boomerang-like distribution, defining two regimes of contact strength. Geometrically, while halogen, chalcogen, and tetrel bonds require σ-hole angles approaching 180° for short contacts, pnictogen bonds are unique, displaying no requirement for linearity at short distances. Additionally, roughly 5% of the complexes exhibit appreciable interaction energies despite intermolecular distances exceeding the sum of their van der Waals radii, confirming that strict distance cut-offs are overly restrictive. Finally, SAPT analysis shows the strongest σ-hole interactions are electrostatically dominated, whereas the weakest are primarily dispersion-driven. Beyond these physical insights, the SHEF1552 dataset can serve as a wide-ranging computational benchmark for geometries and energies of σ-hole interactions.

## Abstract 2

The SHEF1552 dataset encompasses 1552 halogen, chalcogen, pnictogen and tetrel complexes. Combining coupled-cluster energetics, SAPT analyses and geometric metrics, it serves as a robust resource for fundamental physical insights.

## Graphical abstract

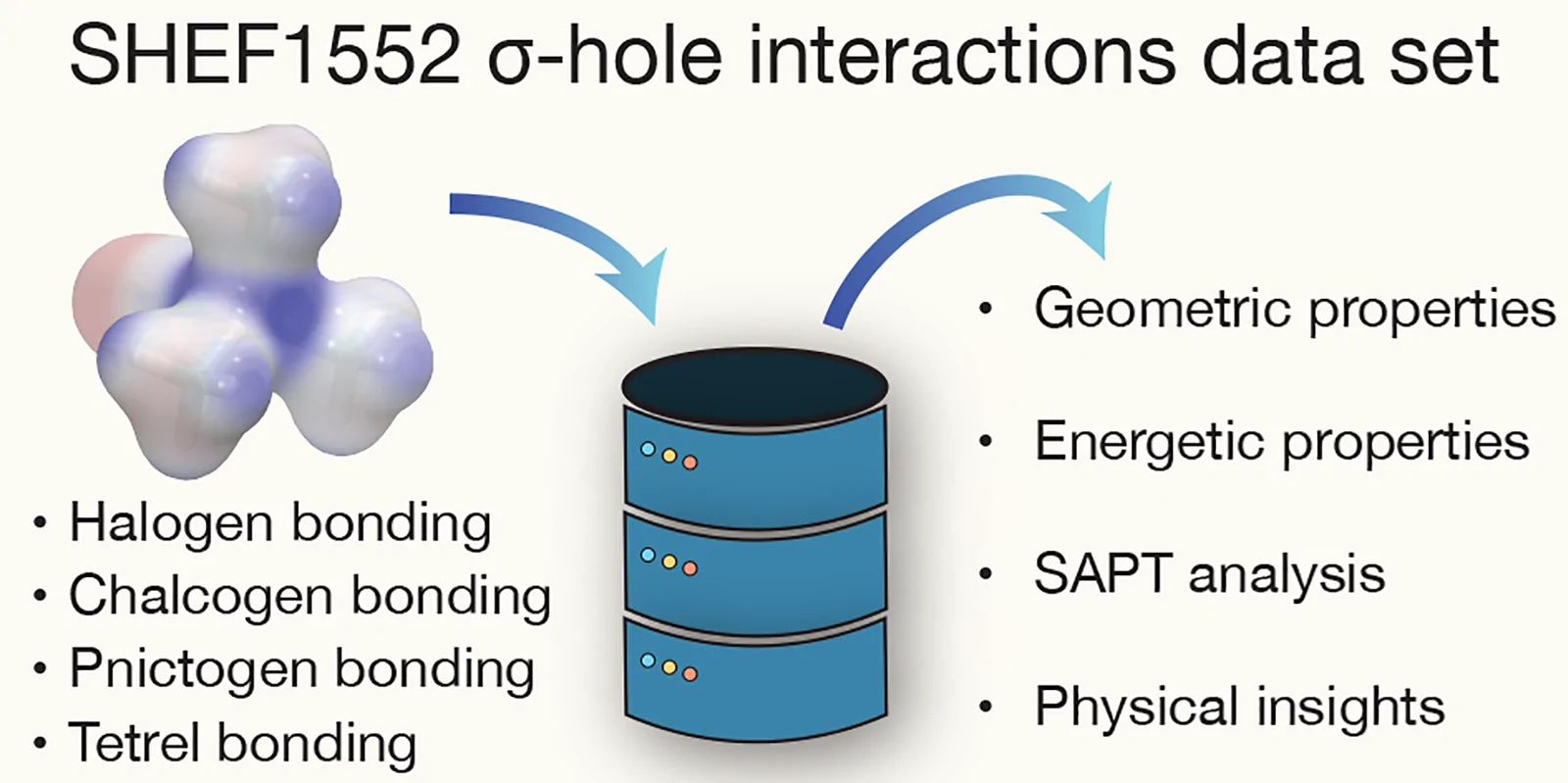

## 1 Introduction

The concept of the σ-hole and the role it plays in the understanding of noncovalent interactions has had a marked effect in fields of both experimental and theoretical chemistry,^1–8^ sparking huge interest and prompting many discussions on terminology and the use of simple models to rationalise the interaction.^9–14^ A σ-hole is a region of reduced electron density found along the extension of a covalent bond,^1,2^ which typically results in a region of positive potential. This phenomenon is often visualised using molecular electrostatic potential maps, which are useful for rationalising the interaction between, for example, a dihalogen and ammonia, and is accessible at the undergraduate level.^3^ An example molecular electrostatic potential for GeCN(Me)_3_ is shown in Fig. 1, where the most positive region is clearly found along the extension of the cyano–Ge σ-bond. Further details on the σ-hole and electrostatic potentials in noncovalent interactions have been addressed in a large number of articles and reviews, and this information is not repeated here. Rather the interested reader is referred to ref. 4–8 and the references therein.

**Fig. 1:**
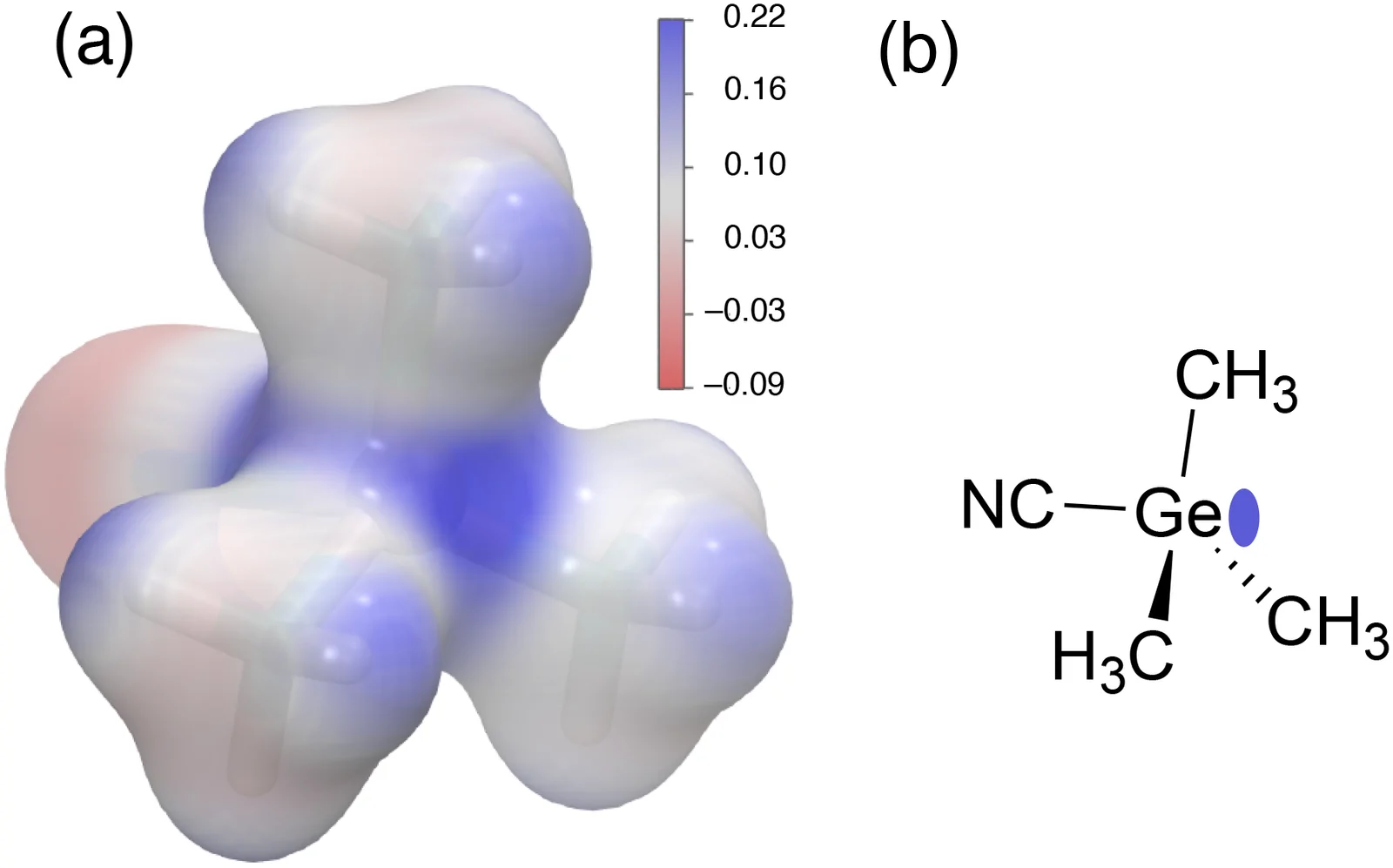
(a) Computed electrostatic potential (a.u.) on the 0.02 a.u. electron density isosurface of GeCN(Me)_3_. (b) Skeletal structure of the same molecule in approximately the same orientation, revealing that the cyano group is positioned to the rear of the molecule. In both representations the blue disc-like area of positive potential along the extension of the cyano–Ge bond indicates the location of the σ-hole.

Perhaps the most well-established category of σ-hole interaction is the halogen bond,^15^ which the International Union of Pure and Applied Chemistry (IUPAC) define as: “*A halogen bond occurs when there is evidence of a net attractive interaction between an electrophilic region associated with a halogen atom in a molecular entity and a nucleophilic region in another, or the same, molecular entity*”.^9^ To make clear the terminology used herein, following the IUPAC definition, the halogen atom will be referred to as the σ-hole donor atom, while the atom or region acting as a nucleophile is the σ-hole acceptor. That each lies within a molecular entity is implicit. Other groups of elements are known to act as donors in σ-hole interactions,^16^ including chalcogens,^11^ pnictogens,^12^ and tetrels,^17^ amongst others. We note that two recent perspectives have suggested that the naming of these interactions by their Periodic Table group and/or element is excessive, with simpler nomenclature such as σ-hole interactions preferred.^13,14^ Although we agree with the principles driving such suggestions, in the present case classifying the interactions into Periodic Table groups (halogen, chalcogen, pnictogen, and tetrel bonds) enables trends, similarities, and divisions to be uncovered.

The IUPAC definition also lists several features of the halogen bond, including that the forces involved in the underlying nature of the interaction are “*primarily electrostatic, but polarization, charge transfer and dispersion contributions all play an important role*”.^9^ This definition also notes that the relative contribution of these terms differ between halogen bonds. As accurately describing all of these contributions is challenging for the majority of quantum chemical methods, combined with the strong interest in σ-hole interactions from both computational and experimental groups, it is perhaps unsurprising that a number of computational datasets have been published that provide optimised geometries and calculated interaction energies for σ-hole interactions. In the majority of cases these datasets are primarily targeted at benchmarking how well approximate computational chemistry methods describe the interactions.

To briefly introduce some of these existing datasets: The GMTKN55 benchmark database contains two relevant subsets;^18^ the PNICO23 set of pnictogen⋯pnictogen noncovalent interactions, which is based on the complexes investigated by Setiawan *et al.*,^19^ and the HAL59 set of noncovalent interactions involving halogenated molecules, which is based on an amalgamation of the XB51 and X40 datasets.^20,21^ We note that these PNICO23 and HAL59 sets include a variety of intermolecular interactions, not just those typically classified as σ-hole interactions. Other important sets include Bauzá *et al.*'s 30-complex set with neutral and anionic σ-hole acceptors and highly fluorinated donors,^22^ Alkorta and Legon's set of 132 intermolecular complexes was used to define a range of “reduced electrophilicities” of Lewis acids,^23^ whereas the CHAL336 set focused on chalcogen-bond interactions,^24^ and concluded that the popular B3LYP and MP2 methods are not recommended for this class of interactions.

More recently, the SH250 × 10 dataset of σ-hole interactions has been added to the wider Non-Covalent Interactions Atlas project.^25^ This dataset contains 250 complexes bound by halogen, chalcogen or pnictogen bonds, each providing a series of ten points along the respective dissociation curve. The σ-hole donor atoms are taken from the third and fourth period of the Periodic Table, plus iodine, and combined with a variety of acceptors. A key limitation of this set for comparative studies is that the set of acceptors used across the halogen, chalcogen and pnictogen bonding complexes is not consistent, meaning that direct comparisons cannot always be drawn. The dataset has been used to benchmark the performance of a large number of DFT functionals, although it also demonstrated that modern semi-empirical quantum chemistry methods such as GFN2-xTB require re-parameterisation to correctly describe the whole breadth of σ-hole interactions. The importance of reusing quantum chemistry benchmark sets, including those dedicated to intermolecular interactions, in the development of force fields for molecular simulations has also been recently highlighted.^26^

Here we present the SHEF1552 (Sigma-hole energies and additional factors) dataset, consisting of 1552 intermolecular complexes, which encompasses halogen, chalcogen, pnictogen and tetrel bonding. The design of the dataset addresses limitations in prior work by focusing on two major goals, which informed the choice of complexes investigated and are prioritised over, for example, the benchmarking of quantum chemical methods:

1. Discovery of new insights into σ-hole interactions in terms of geometric and energetic properties, and the underlying nature of the interaction.

2. Relevance to both theoretical and experimental chemists/material scientists.

The use of the same set of acceptor molecules paired with each donor molecule is essential so that reliable comparisons can be drawn both between classes of interaction (halogen bond, chalcogen bond, *etc.*) and within each of those classes. The lightest elements from each class, from the second period of the Periodic Table, are not included as σ-hole donor atoms as they typically only form very weak interactions. In terms of the heavy elements from the sixth period, we only consider complexes where Pb or Bi act as the donor atom. Both Po and At are highly radioactive and, although there may be some interest in the hypothetical complexes they may form, they are excluded here as they are less relevant to experimental studies. Similarly, we generally avoid adding fluorine substituents *directly* to the donor atoms. It is well-known that these strong electron-withdrawing groups enhance σ-hole interactions, but highly fluorinated compounds pose substantial experimental challenges and such species are less representative of those involved in interactions found in, for example, the Cambridge structural database (CSD).^27^

Further details of the complexes in the dataset and the computational methods used are presented in Section 2. The results of the calculations enable a systematic analysis of these 1552 intermolecular σ-hole complexes and uncover overarching energetic and geometric trends across the four interaction classes. Most notably, the data reveal a dual-regime relationship between energy and distance, highlight the non-linear character of pnictogen bonds, and expose the limitations of traditional van der Waals distance cut-offs.

## 2 Methods

### 2.1 Dataset construction

The SHEF1552 dataset was designed to cover a broad and representative chemical space for σ-hole interactions. The choice of σ-hole donor molecules was partly informed by our experience of CSD searches for σ-hole interactions.^28^ Small neutral molecules that represent commonly-found motifs within the CSD were selected, with each of seven halogen-bonding donor motifs appearing three times (one each for Cl, Br and I), each of seven chalcogen-bonding donor motifs three times (S, Se and Te), each of five pnictogen-bonding donor motifs four times (P, As, Sb and Bi), and each of five tetrel-bonding donor motifs four times (Si, Ge, Sn and Pb). This resulted in a total of 82 σ-hole donor molecules (21 halogen bonding, 21 chalcogen bonding, 20 pnictogen bonding, and 20 tetrel bonding), with a full list of the molecules presented in Tables S1–S4.

All of these σ-hole donor molecules are then paired with a Lewis base/σ-hole acceptor, such that each donor is paired with each possible acceptor. The 19 Lewis bases selected are: ammonia; phosphine; hydrogen cyanide; acetone; thioacetone; trimethylamine; aniline; furan; pyridine; dioxane; water; acetonitrile; thiophene; imidazole; trimethylphosphine; trimethylphosphine sulfide; trimethylphosphine oxide; nitromethane; pyridine-*N*-oxide. Permuting over all of these possibilities results in a total of 1558 complexes, comprised of 399 halogen-bonding complexes, 399 chalcogen, 380 pnictogen, and 380 tetrel. After analysis of the optimised geometries, six of the complexes were removed as their structures did not appear to be consistent with σ-hole interactions. Further details of the structures removed are provided in the SI (Section S5).

All complexes were initialised in a linear arrangement by constructing the σ-hole donor molecule such that the extension of the σ-hole is aligned along the *z*-axis in the negative direction, and the acceptor/Lewis base with the lone pair along the *z*-axis in the positive direction. The initial separation of the σ-hole donor atom and the acceptor atom was set to 85% of the sum of the van der Waals radii of these interacting atoms.

The geometry of each σ-hole complex was subsequently optimised using the M06-2X exchange correlation functional with the D3 dispersion correction [zero damping, D3(0)],^29,30^ and the def2-TZVPD basis set.^31–34^ All geometry optimisations were carried out in ORCA version 5.0.4,^35^ and further technical details of the calculation can be found in the SI (Section S3). The M06-2X-D3(0) functional was chosen as it has demonstrated good performance for intermolecular interactions, including the SH250 dataset, and offers a favorable balance of accuracy and computational efficiency.^18,25,36^

The nature of each stationary point located was ascertained by calculating the harmonic vibrational frequencies at the same level of theory. Structures possessing imaginary frequencies were distorted along the associated normal mode(s) and re-optimised until a minimum was found. After several cycles of optimisation and distortion, 34 structures still possessed low frequency imaginary modes, generally associated with propeller-like rotation of, for example, methyl groups not directly involved in the intermolecular interaction. As such, these structures were retained in the database and are identified in Table S6.

Two key geometric properties were analysed: the intermolecular distance (*d*) between the donor and acceptor atoms, and the σ-hole angle (*θ*). The σ-hole angle is defined as R–D⋯A, where R forms a σ-bond to the σ-hole donor atom (D), and A is the acceptor atom (Fig. 2). To allow for a direct comparison between interactions involving different atom types, we have used a dimensionless, normalised distance (*d*_norm_):^37,38^
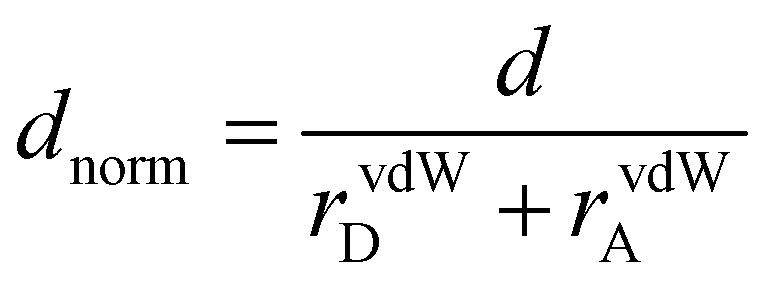
where *r*^vdW^_D_ and *r*^vdW^_A_ are the van der Waals radii of the donor and acceptor atoms, respectively, as tabulated by Alvarez.^39^

**Fig. 2:**
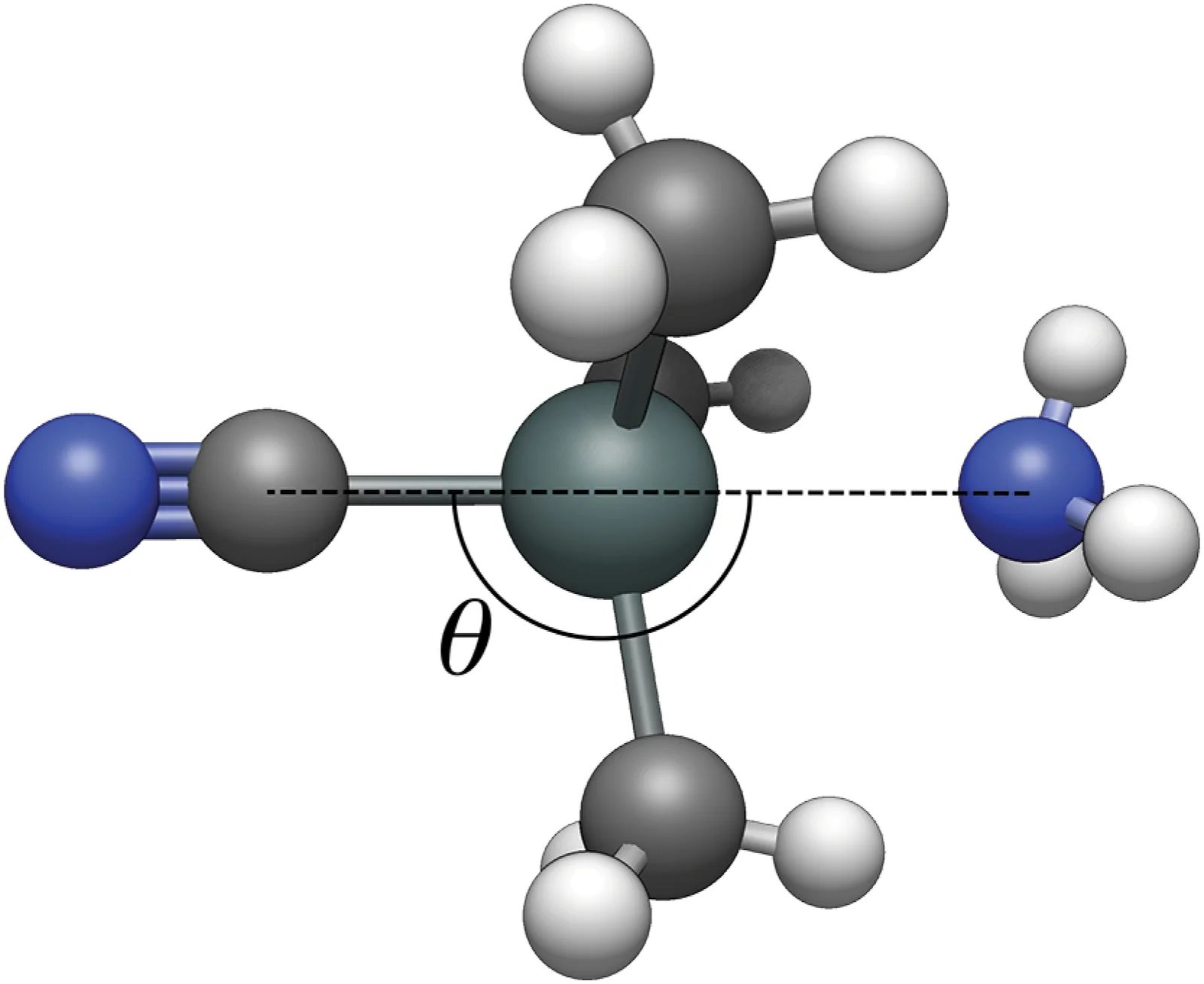
Definition of the σ-hole angle (*θ*) of R–D⋯A, where D is the σ-hole donor atom, R is the electron withdrawing group involved in the formation of the σ-hole, and A is the acceptor atom. The complex shown is NC(Me)_3_Sn⋯NH_3_.

### 2.2 Interaction energies

No attempt was made to incorporate the effects of spin–orbit coupling into the calculation of interaction energies or within geometry optimisations. This will affect the results of complexes containing heavier elements, although a recent study of chalcogen bonding suggests this would lead to a relatively small, albeit non-trivial, reduction in the strength of interaction.^40^

#### 2.2.1 Density functional theory

Interaction energies at the optimised geometries were determined at the M06-2X-D3(0) level using the same basis sets, settings and software as for the geometry optimisations. In this supramolecular approach to interaction energies, the individual molecules were fixed in their interacting geometries and the counterpoise (CP) correction was used to account for basis set superposition error.^41^

#### 2.2.2 Coupled-cluster theory

The most accurate interaction energies for the SHEF1552 dataset were determined using the explicitly correlated local coupled-cluster methodology and can be used to benchmark more approximate calculations. Specifically, for the coupled-cluster calculations we used the PNO-LCCSD(T*)-F12b method [further abbreviated as LCCSD(T*)-F12] that employs pair natural orbitals.^40,42–61^ Full technical details of coupled-cluster interaction energy calculations, including the use of tight domains, outer-core correlation, and the specific mixed basis set (aVDZ-F12) utilised, can be found in the SI (Section S3.2). This level of theory was selected because, despite using a double-zeta basis set, it achieves near-complete basis set limit quality with high computational efficiency. Although this approach shows exceptional accuracy on general non-covalent interaction benchmarks such as the S66 set (RMSD = 0.05 kcal mol^−1^),^62–64^ its suitability for σ-hole interactions was verified using the SH250 dataset.^25^ Against composite CCSD(T)/CBS reference values this level of theory achieves an RMSD of 0.23 kcal mol^−1^. Additionally, basis set convergence was directly evaluated on a stratified sample of 28 complexes from the SH250 dataset. Expanding the basis set to aug-cc-pVTZ-F12 (without triples scaling) reduced both the RMSD and mean absolute deviation by only 0.03 kcal mol^−1^ (Section S7 of the SI), confirming that basis set incompleteness error is very small, even at the double-zeta level. All coupled-cluster calculations were carried out in the Molpro package of *ab initio* programs, version 2023.2.^65,66^

#### 2.2.3 Symmetry-adapted perturbation theory

Additional insights into the underlying physical nature of the intermolecular interactions were obtained through symmetry-adapted perturbation theory (SAPT).^67^ SAPT2 calculations were carried out using the PSI4 program package version 1.8.2,^68^ with the def2-TZVPD basis set for all heavy atoms. Full technical details of these calculations are provided in the SI. The individual SAPT terms are grouped into an electrostatic component (*E*_elec_), an exchange component (*E*_exch_), an induction component (*E*_ind_), and a dispersion component (*E*_disp_), following the standard “chemist's grouping”.^69^

## 3 Results and discussion

The SHEF1552 dataset is large enough to provide chemically valuable information on trends in the geometries and energies of σ-hole interactions, in addition to serving as a benchmark dataset for future computational studies of such interactions.

### 3.1 Geometric parameters

The two principal defining geometric parameters for σ-hole interactions are the interaction distance, presented here as a normalised value (*d*_norm_ – see Section 2.1) to enable facile comparison across different class of interaction, and the σ-hole angle (*θ*). These geometric parameters for all 1552 complexes are plotted against each other and shown, along with kernel density estimate plots of the distributions grouped by interaction class in Fig. 3 (Further numerical and statistical details in Table S8).

**Fig. 3:**
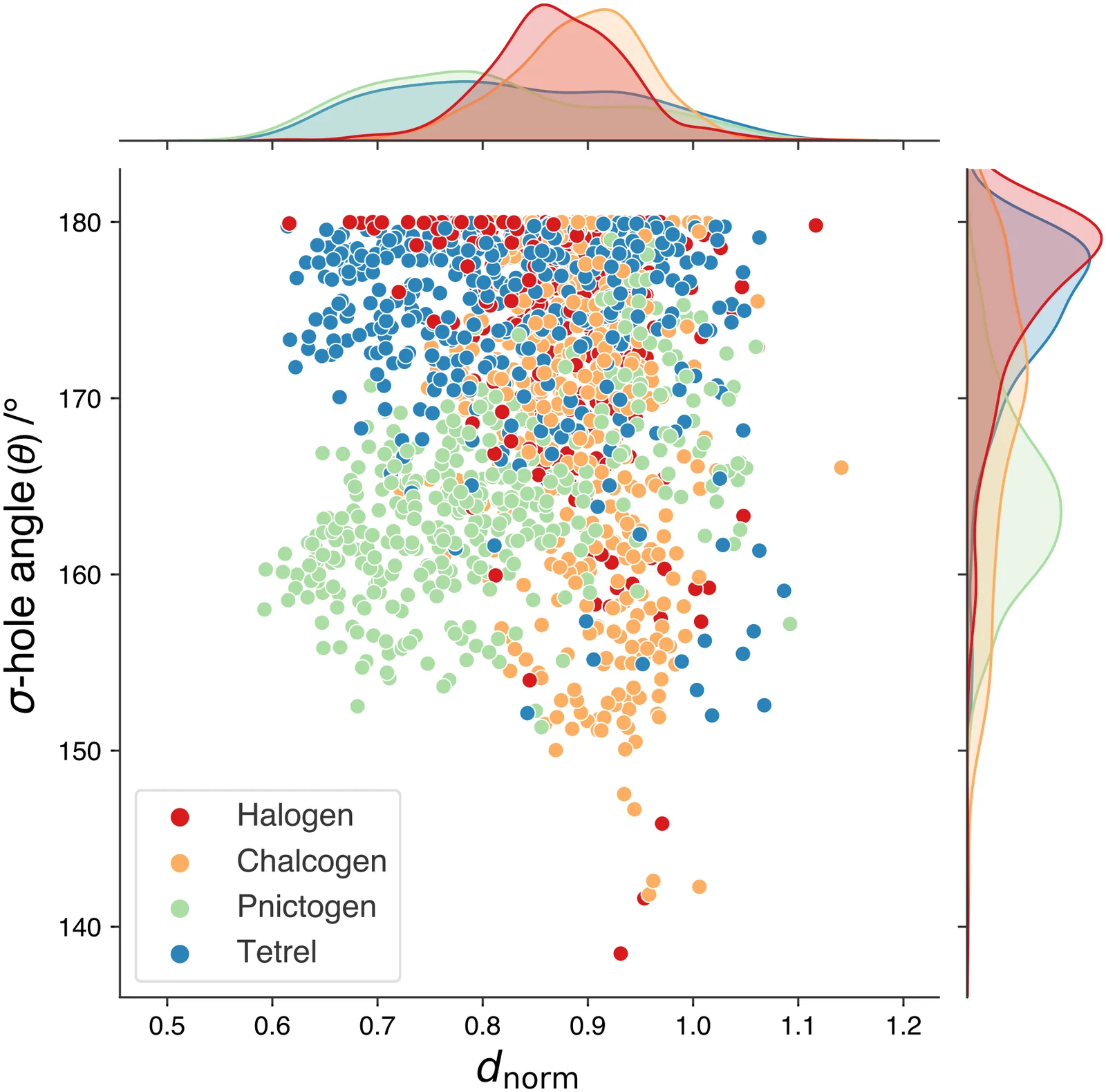
Normalised distance (*d*_norm_) and σ-hole angle from M06-2X-D3(0)/def2-TZVPD optimised geometries for the 1552 complexes in the dataset. Also shown are kernel density estimate plots of the distribution of the normalised distances and σ-hole angles grouped by interaction class.

Analysis of the *d*_norm_ distribution reveals key differences between the four classes. Pnictogen bonds are, on average, the shortest (*d̄*_norm_ = 0.805) followed closely by tetrel bonds (*d̄*_norm_ = 0.827). Halogen (*d̄*_norm_ = 0.872) and chalcogen bonds (*d̄*_norm_ = 0.892) exhibit similar average distances, both being slightly longer than the pnictogen and tetrel interactions. The distributions for tetrel and pnictogen bonds are visibly broader and possess some bimodal character, whereas halogen and chalcogen bond distributions are sharper. Despite these differences, all four classes span similar relatively wide ranges of *d*_norm_ values (Table S8). In terms of σ-hole angle, tetrel (*θ̄* = 174.4°) and halogen bonds (*θ̄* = 175.2°) are the closest to linearity. Pnictogen bonds show a noticeable deviation from linearity (*θ̄* = 164.4°), with a clearly defined peak around the mean value (Fig. 3). Chalcogen bond interactions display the widest distribution of angles (*θ̄* = 168.1°, with a standard deviation of 8.7°).

To explore how representative these computational gas-phase structures are, we conducted searches of the CSD [database version 5.46 (November 2024)].^27,70,71^ The queries targeted σ-hole interactions to so-called “divalent oxygens”, which have a R–O–R′ motif and describe a wide variety of potential σ-hole acceptors. Full technical details of the CSD search are provided in the SI (Section S5.2). To facilitate a meaningful comparison with SHEF1552, we applied a relatively aggressive search filter, retaining only hits with a σ-hole angle ≥ 150° and a *d*_norm_ ≤ 0.93, resulting in geometric data for 1541 σ-hole interactions. The visualisation of the raw and filtered data in terms of *d*_norm_ and *θ* is provided in Fig. S4.

Comparison of the SHEF1552 computational dataset and the CSD experimental data show strong qualitative agreement in *d*_norm_ distributions (Fig. 4). For instance, both show the bimodal distributions of distances for pnictogen and tetrel bonds. Similarly, the relative ordering of the σ-hole angle distribution maxima is preserved across both datasets (pnictogen < chalcogen < tetrel < halogen; Fig. S5). The narrower distribution of pnictogen-bond angles is notable in both datasets. Bearing in mind this is a comparison between computational gas-phase and experimental crystal structures, these Figures provide confidence that the structures in SHEF1552 are relevant to experimental chemists.

**Fig. 4:**
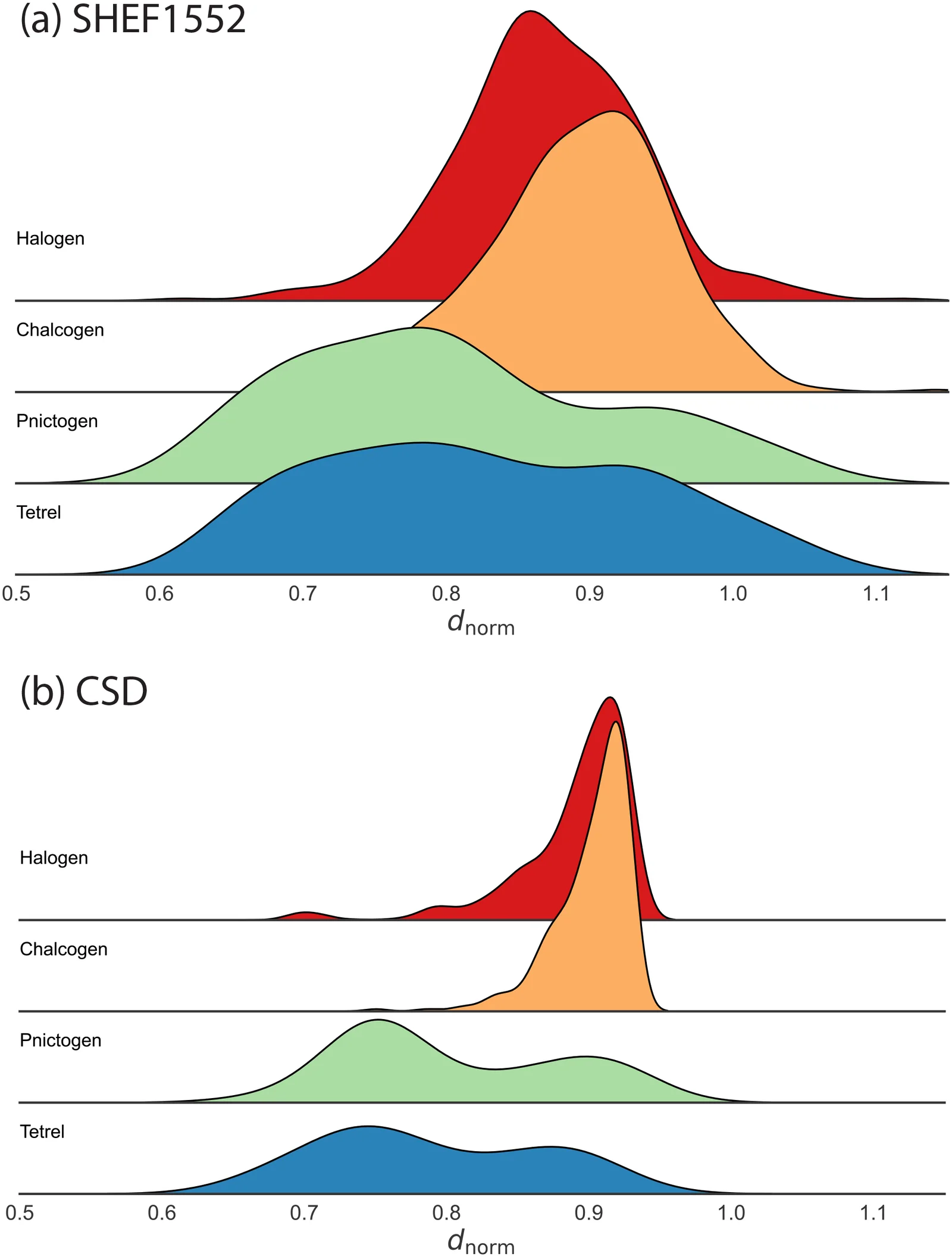
Kernel density estimate plots, per interaction class, of the distribution of normalised distances (*d*_norm_) found in: (a) the SHEF1552 dataset of computational gas-phase structures, (b) a subset of the CSD search results for σ-hole interactions with divalent oxygen as the acceptor atom on the Lewis base.

A commonly used criterion for the presence of an intermolecular interaction is that *d*_norm_ ≤ 1.0. In SHEF1552, however, ∼5% (81 complexes) of the interactions have *d*_norm_ > 1.0 (Fig. 3). Several of these longer interactions have σ-hole angles close to 180° and possess appreciable interaction energies (some >5 kcal mol^−1^). These 81 structures, retained in SHEF1552, add further evidence to the growing consensus that a strict *d*_norm_ ≤ 1.0 cut-off is overly restrictive for defining σ-hole interactions,^2,39,72,73^ which aligns with the prevailing view for hydrogen bonds.^74–76^

Clear differences in the geometric requirements for the four interaction classes become apparent when mapping *d*_norm_ against σ-hole angle. These relationships are visualised in the scatter plots of Fig. 5, which are further subdivided by donor atom. Halogen-, chalcogen- and tetrel-bonding interactions all display a similar “wedge”-shaped population of the scatterplot. At short *d*_norm_ values, the σ-hole angles are close to 180° (upper left of the plots) and as *d*_norm_ increases, the complexes form an approximately diagonal “interaction front”, indicating that longer distances permit a greater deviation from linearity. This qualitative relationship (shown as dashed grey lines), similar to that previously reported from crystal structure data for a set of halogen-bonding interactions to phosphine oxides,^77^ varies in gradient and position between the three classes of σ-hole interaction. This diagonal relationship is analogous to the well-established directional trends observed for hydrogen bonds.^78,79^ In stark contrast, pnictogen-bonding interactions do not show this relationship between *d*_norm_ and σ-hole angle, meaning short pnictogen bonds have no apparent requirement to be linear.

**Fig. 5:**
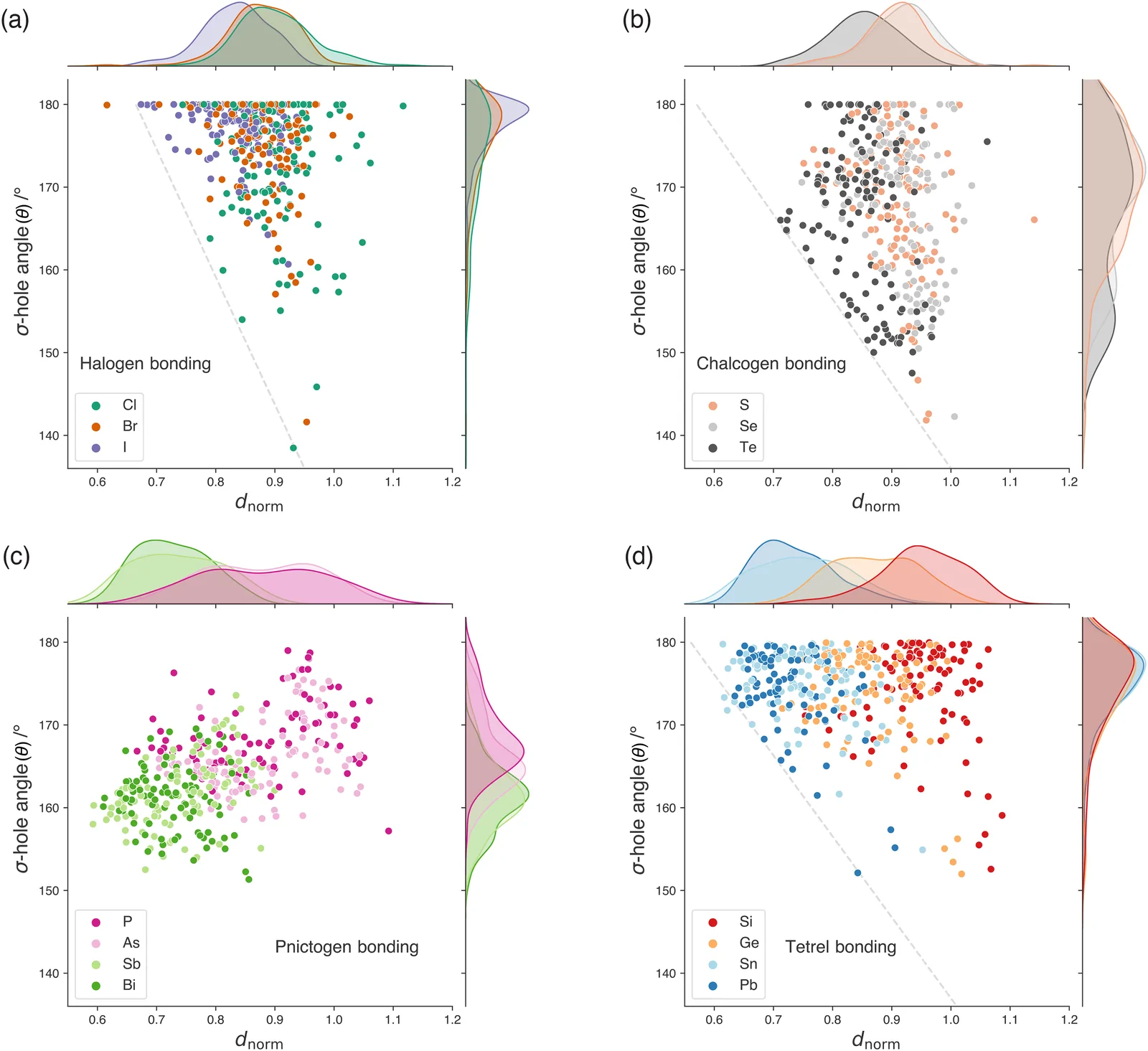
Normalised distance (*d*_norm_) and σ-hole angle (*θ*) from M06-2X-D3(0)/def2-TZVPD optimised geometries subdivided into (a) halogen, (b) chalcogen, (c) pnictogen and (d) tetrel bonding interaction classes. Each of these interaction classes are also shown as kernel density estimate plots grouped by σ-hole donor atom. The dashed grey lines for halogen, chalcogen and tetrel bonds highlight the diagonal “interaction front”, which indicates shorter distances require σ-hole angles closer to 180°.

Although the fronts highlighted in Fig. 5 are mostly to guide the eye towards a qualitative relationship between *d*_norm_ and the minimum σ-hole angle, they may also be useful in identifying points of interest. For example, there is a halogen bond involving bromine that sits noticeably to the left of the interaction front, which may indicate an interaction that is stronger than expected. This point corresponds to the complex between *N*-bromosuccinimide and trimethylphosphine, which will be further discussed in the Section 3.2 on Interaction Energies. The diagonal interaction front for the chalcogen interactions also suggests that there may be area of interesting chemical space that the dataset does not fully explore. The upper left of the scatter plot has an area to the right of the diagonal front where no interactions are found. This suggests it may be possible to engineer chalcogen bonds that have shorter distances (while possessing a σ-hole angle close to linearity) than those from this dataset, although such a qualitative picture provides no information on the exact nature of σ-hole donor and acceptor required to do so. Nor does it account for any additional factors, for example, steric effects, that may prevent interactions in this region.

The subdivisions by donor atom in Fig. 5 also provide insights into intra-group trends (see also Tables S9 and S10). For halogen bonds, iodine consistently produces the shortest *d*_norm_ values and the closest to linear interactions, while bromine and chlorine distributions are similar to each other. This trend is consistent with the interaction strength following the polarisability of the halogen atom (Cl < Br < I).^15,80–82^ Chalcogen bond trends are analogous: tellurium (most polarisable) has shorter *d*_norm_ values than selenium and sulfur. Pnictogen bonds clearly form two groups: the heavier elements (Sb, Bi) have shorter normalised distances than the lighter elements (P, As). The pnictogen σ-hole angles follow a general trend of Bi ≈ Sb < As < P. Finally, for tetrel bonds, the shortest interactions are observed for Sn, followed by Pb. This does match the trend in polarisabilities (Sn = 53 a.u., Pb = 47 a.u.),^83^ but the mean *d*_norm_ for Pb (0.736) is slightly shorter than that of Sn (0.750). Interactions involving Ge are somewhat longer, and are longer still for Si, which does match expectations from polarisability alone.

### 3.2 Interaction energies: relationship to geometries

The interaction energy for each of the 1552 complexes was computed at the coupled-cluster level of theory [CP-corrected LCCSD(T*)-F12]. This method produced an RMSD of 0.05 kcal mol^−1^ for the established S66 set of intermolecular interactions,^64^ and is designated as the benchmark level of theory for the SHEF1552 dataset. The interaction energies provide both a quantitative measure of the interaction strength and reveal its relationship with the geometric parameters (Fig. 6) for SHEF1552.

**Fig. 6:**
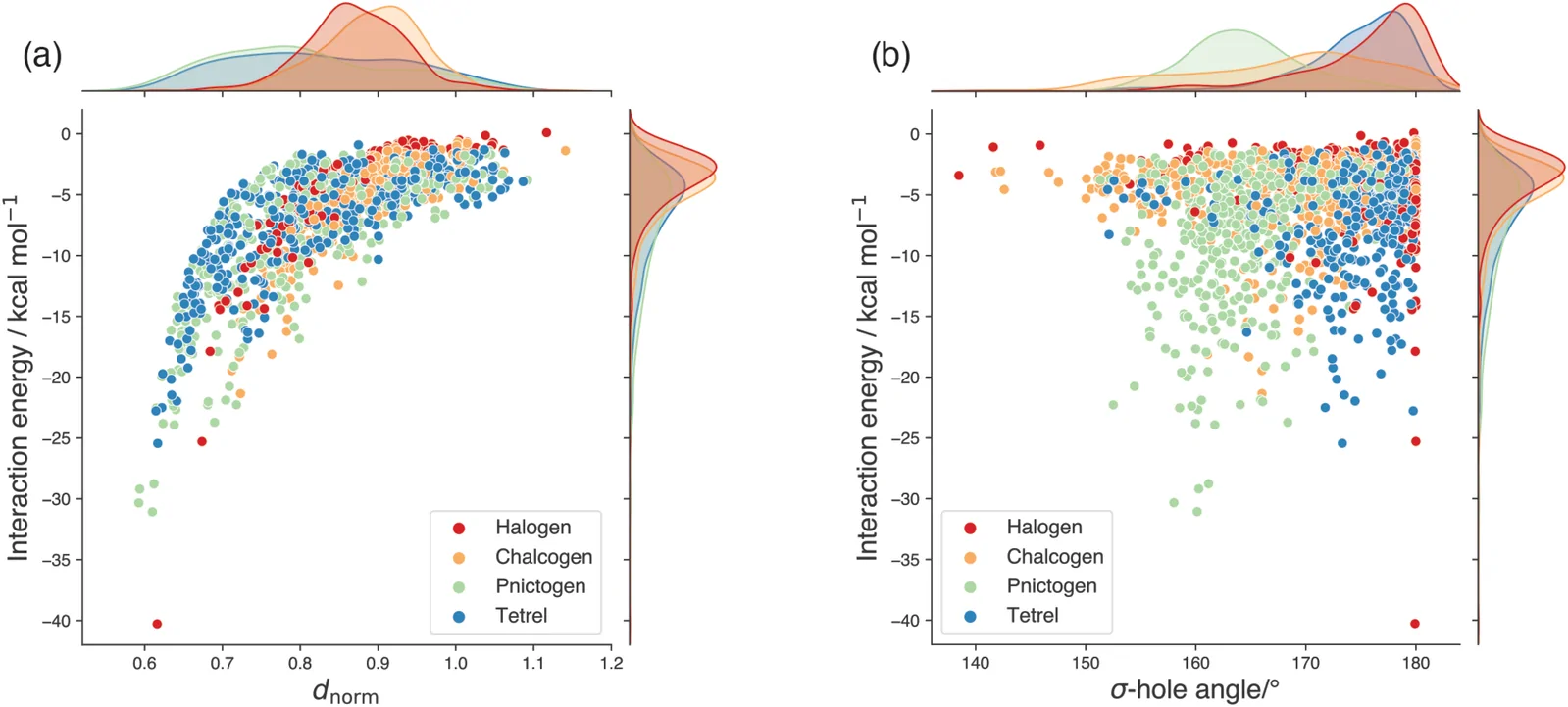
Counterpoise-corrected LCCSD(T*)-F12 interaction energies plotted against: (a) normalised distance (*d*_norm_); (b) σ-hole angle. All geometries were optimised at the M06-2X-D3(0)/def2-TZVPD level of theory. Also shown are kernel density estimate plots of the distributions grouped by interaction class.

The relationship between interaction energy and *d*_norm_ [Fig. 6(a)] shows, unsurprisingly, an overall trend in which shorter normalised distances lead to stronger interactions. More revealingly, the overall distribution adopts a distinctive boomerang-like shape that indicates two regimes of interaction strength (see Fig. S8 for an exponential function fit to this distribution). Below *d*_norm_≈0.8, the strength increases rapidly with decreasing *d*_norm_ (∼48% of complexes stronger than −10 kcal mol^−1^). In contrast, at *d*_norm_ ≥ 0.8, interactions are generally weaker than −10 kcal mol^−1^, indicating that the *d*_norm_ = 0.8 region effectively divides weak/moderate σ-hole interactions from the stronger complexes.

Mean interaction strengths follow the clear trend: halogen bonds < chalcogen bonds < tetrel bonds < pnictogen bonds (see summary statistics in Table S11). It is worth noting that if the average normalised distance was used as the sole factor to judge the strength of interaction, it would appear that halogen-bond interactions are stronger than chalcogen bonds, which is inverted relative to the mean interaction energies. Furthermore, comparison across the dataset shows phosphorous-based pnictogen bonds exhibit a stronger mean interaction energy than iodine-based halogen bonds (−6.75 *vs.*−5.45 kcal mol^−1^). Although some care is needed to avoid overreaching conclusions from considering the seven iodine-based donors (133 complexes) and the five phosphorous-based donors (95 complexes), the fact that mean interaction energies involving CH_3_I or C_6_H_5_I are weaker than those involving PMe_3_, and CF_3_I are weaker than those involving PH_2_F suggests an under-appreciated strength of phosphorous as a σ-hole donor and underscores the potential of pnictogen bonding more broadly in supramolecular chemistry.

Fig. 6(b) displays the variation of interaction energy with σ-hole angle. Excluding pnictogen bonds (as seen in Fig. S6), the distribution is wedge-like: stronger interactions tend to be close to linear, while smaller σ-hole angles (more bent geometries) correspond to weaker interactions. Pnictogen bonds are distinct, having a narrow angular distribution (*θ̄* = 164.4°), they show seemingly little dependence of interaction energy on the σ-hole angle.

This geometric behaviour can be qualitatively rationalised using VSEPR-type arguments (Fig. 7). Halogen and tetrel bond interactions, which have σ-hole axes that are extensions of covalent bonds to the most electron-withdrawing substituents, predictably favour angles close to 180° due to minimised steric and electronic repulsion [Fig. 7(a) and (d)] and maximised electrostatic attraction. Chalcogen bonds, which involve divalent donors [Fig. 7(b)], exhibit a wider angle distribution due to the possibility of secondary interactions and fewer strict geometric constraints. For trivalent pnictogens [Fig. 7(c)], the approaching Lewis base encounters repulsion from the pnictogen lone-pair, which promotes deviation from linearity. This lone-pair repulsion, combined with steric interactions from the two substituents, constrains the σ-hole angle to the narrow range around the 164.4° mean. Geometric evidence supporting this deviation from linearity caused by lone-pair repulsion is provided in Section S5.1 of the SI.

**Fig. 7:**
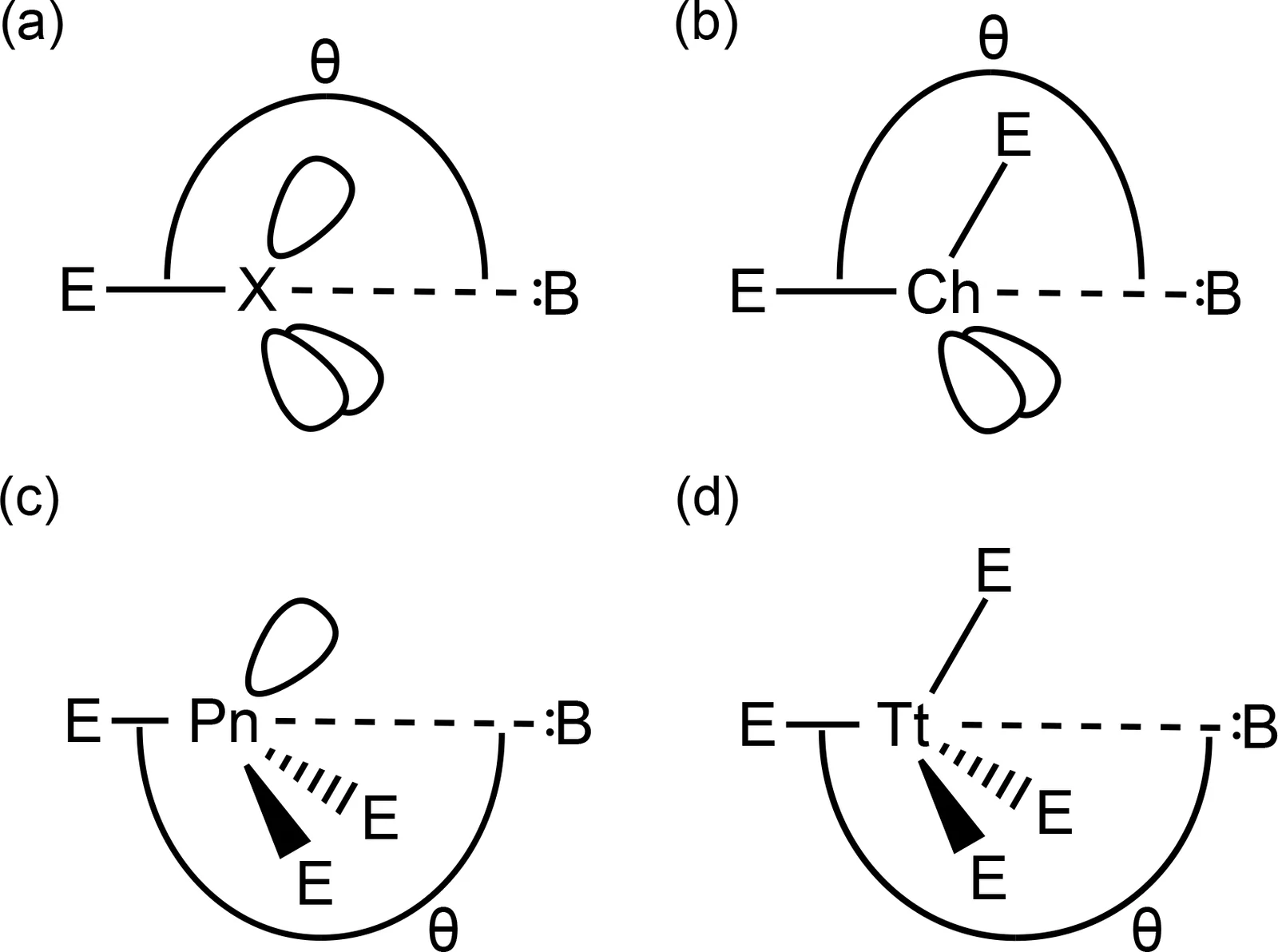
VSEPR-style diagrams, explicitly showing lone-pairs, for the interaction of a σ-hole donor molecule with a Lewis base, B. The Sigma-hole angle, *θ* is also highlighted. The donor molecules are: (a) a halogen bond (X) donor; (b) a chalcogen bond (Ch) donor; (c) a pnictogen bond (Pn) donor; (d) a tetrel bond (Tt) donor. E represents any substituent/element, and the dashed line defines the σ-hole axis.

Individual plots of interaction energy against *d*_norm_ for the four interaction classes are presented in Fig. S7 (summary statistics Table S12). The energetic trends within each interaction class align with the polarisability trend of the donor atom. One notable point is that interactions of the lightest, least polarisable elements of the third period are weaker than those of the fourth period (Table S12), which is less obvious when inspecting the normalised distances alone (Table S9).

The use of *d*_norm_ ⩽1.0 as a crude interaction criterion would exclude ∼5% (81 complexes) of the SHEF1552 dataset. Although the LCCSD(T*)-F12 interaction energies of these 81 complexes show that some are weaker than −1 kcal mol^−1^ (Fig. 8), the mean interaction energy for all of these longer-ranged interactions is −2.70 kcal mol^−1^. This confirms that σ-hole interactions can occur at distances greater than the sum of the van der Waals radii, justifying their inclusion in SHEF1552 and adding extra evidence against rigid distance thresholds.^2,39,72,73^

**Fig. 8:**
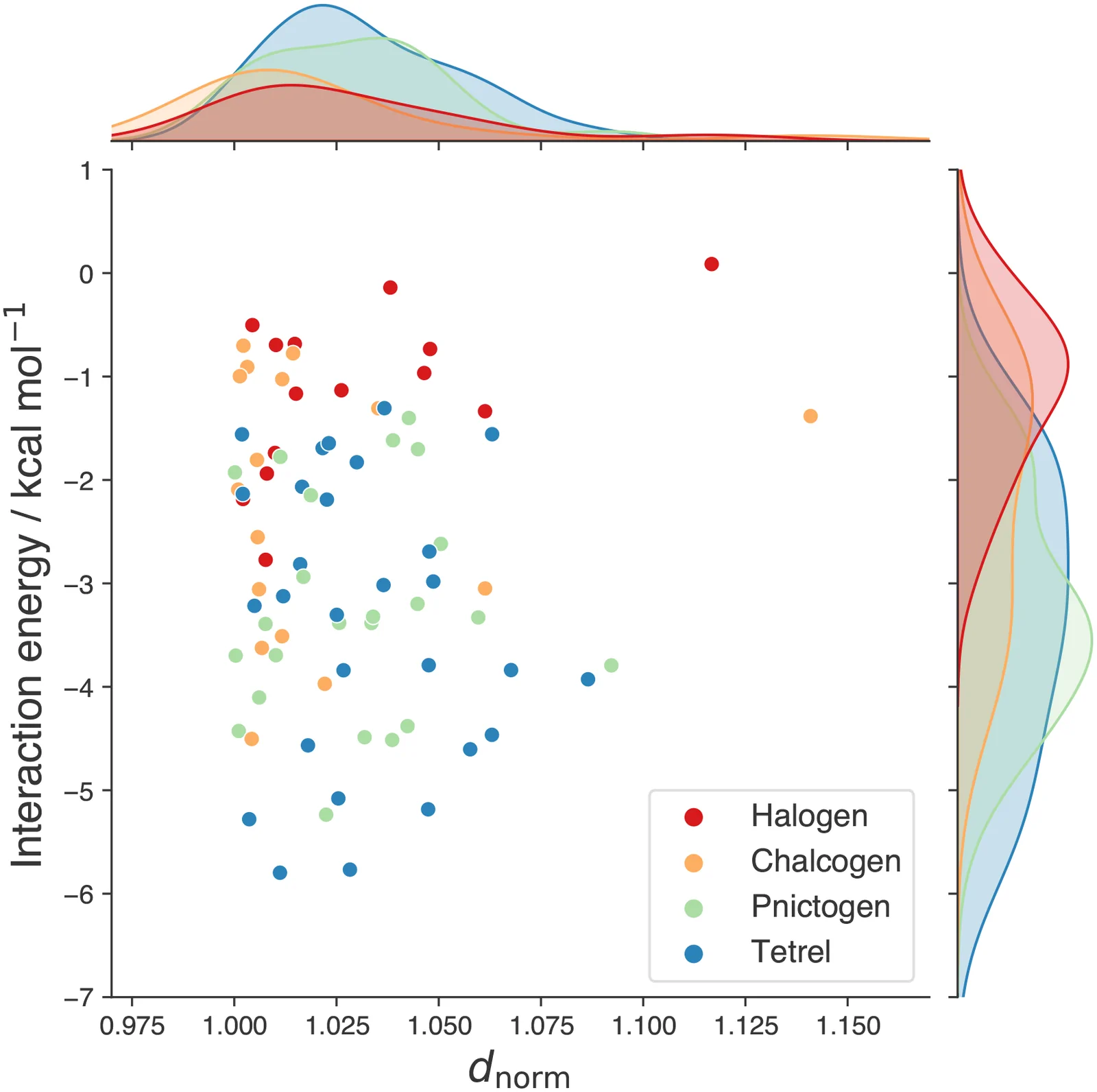
Counterpoise-corrected LCCSD(T*)-F12 interaction energies plotted against *d*_norm_ values for the complexes with. All geometries were optimised at the M06-2X-D3(0)/def2-TZVPD level of theory. Also shown are kernel density estimate plots of the distributions grouped by interaction class.

The strongest interaction within the dataset, the complex between *N*-bromosuccinimide (NBS) and trimethylphosphine (PMe_3_), has an interaction energy of −40.27 kcal mol^−1^. Its optimised geometry is shown in Fig. 9. NBS is used to introduce bromine (nominally as Br^+^) in both electrophilic addition and substitution reactions,^84^ hence interacting strongly with a soft Lewis base containing methyl groups is perhaps not surprising. Crystal structures of the *N*-halosuccinimides also indicate that NBS and *N*-iodosuccinimide (NIS) are strong halogen bond donors, whereas *N*-chlorosuccinimide (NClS) is markedly weaker.^85,86^ The interaction energies for the NIS⋯ and NClS⋯PMe_3_ complexes in SHEF1552 are −25.29 and −4.13 kcal mol^−1^, respectively.

**Fig. 9:**
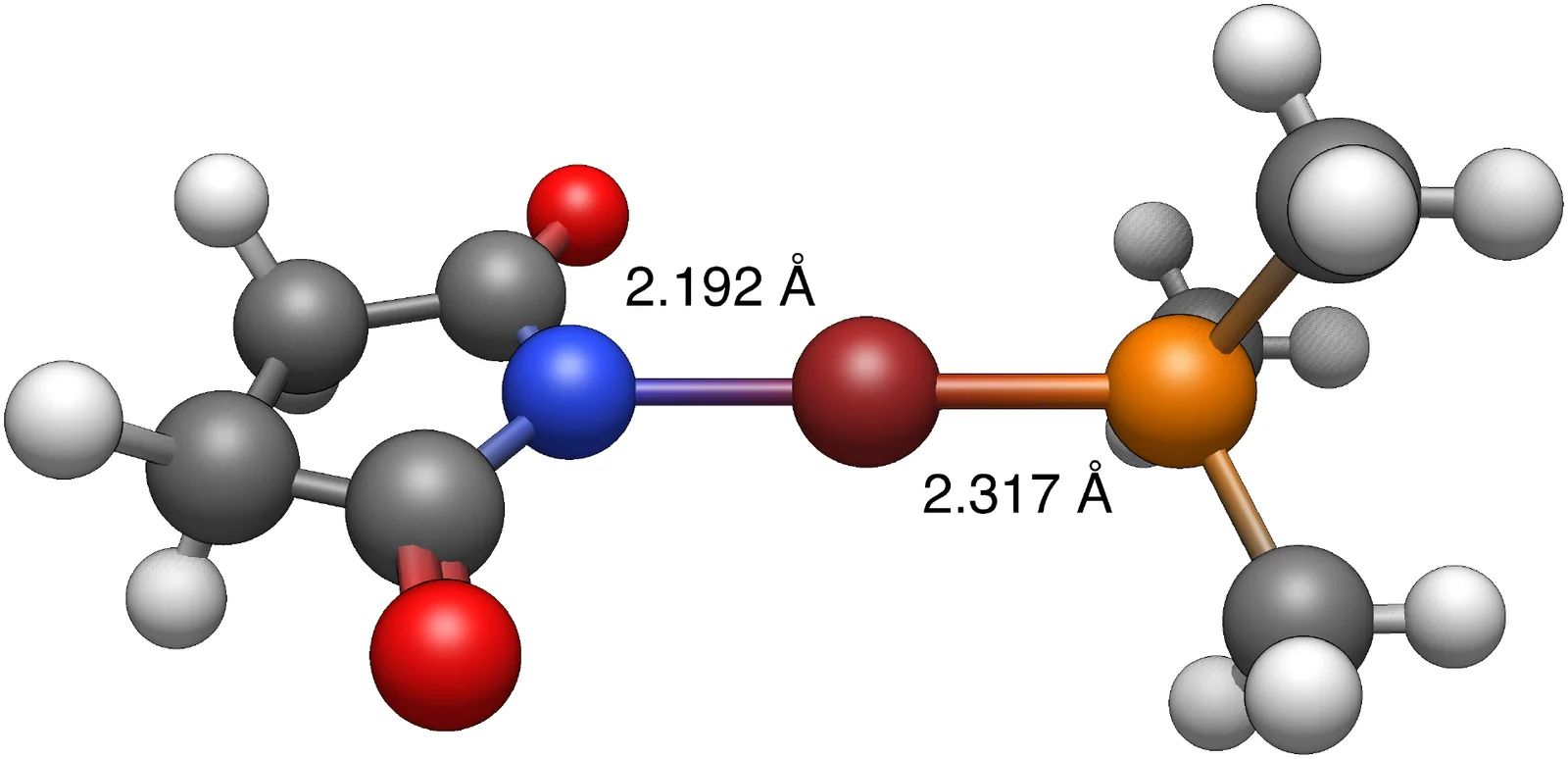
The *N*-bromosuccinimide⋯trimethylphosphine complex (structure 1325 in the dataset) optimised at the M06-2X-D3(0)/def2-TZVPD level of theory. The nitrogen-bromine (upper) and bromine-phosphorus (lower) interatomic distances are noted in the image. The molecular visualisation software, Avogadro2,^87,88^ has drawn covalent bonds from the bromine to both the nitrogen of the succinimide and to the trimethylphosphine. These lines are based on the interatomic distance falling within the sum of the covalent radii plus 0.45 Å.

Surprisingly, the strongly bound NBS⋯PMe_3_ complex has a much stronger interaction than its iodine analogue, inverting the expected halogen bonding trend. This can be attributed to a dual role of the PMe_3_ methyl groups. Electronically, the methyl groups are electron donating, which enhances the Lewis basicity of the phosphorous lone pair compared to PH_3_ and drives interactions energies from −4.00 kcal mol^−1^ (for NBS⋯PH_3_) to −40.27 kcal mol^−1^. However, accessing this lone pair carries a steric penalty. Geometric analysis shows that the smaller bromine is able to approach closely (*d*_norm_ = 0.616), while SAPT analysis explicitly captures the physical consequence of this approach, with the electrostatic, induction and exchange-repulsion terms for the NBS complex roughly double those of the NIS complex. In contrast, the larger iodine centre clashes with the steric bulk of the methyl groups, preventing it from fully exploiting the activated Lewis base (*d*_norm_ = 0.674). This is confirmed by the sterically unhindered PH_3_ analogues within the dataset, where the absence of methyl groups restores the expected I > Br energy trend.

To further characterise the interaction between NBS and PMe_3_, we used the interpenetration index (*p*_AB_, see SI, Section S6.2) developed by Alvarez and co-workers,^89,90^ which measures the degree to which two atoms' van der Waals crusts overlap. The index defines the sum of the van der Waals radii of the interacting atoms as 0% interpenetration and the (single) covalent bond length as 100%. The calculated *p*_AB_ for the NBS⋯PMe_3_ complex is 96.88%, confirming a geometry very close to a single covalent bond and rationalising the strength of the interaction. Such pronounced covalent character is known to invert the expected halogen-bonding hierarchy between iodine and bromine, as previously observed in similarly strong adducts.^91^

Beyond this specific case, analysis of the entire dataset shows that *p*_AB_ is a superior distance-based descriptor for interaction strength compared to *d*_norm_. Indeed, a simple fifth-order polynomial fit achieves an RMSE of 2.25 kcal mol^−1^ for interaction energy *vs. p*_AB_ (Fig. S9). Although more sophisticated models for predicting the strength of halogen-bonding interactions already exist,^92–95^ the strong correlation demonstrated here is promising for a wider predictive model of σ-hole interaction strength based upon geometry.

### 3.3 Interaction energies: computational benchmarking opportunities

Beyond providing chemical and physical insights, the high-level LCCSD(T*)-F12 interaction energies within SHEF1552 can be used as a reference dataset to benchmark lower-cost computational approaches, such as DFT functionals, aiming to identify methods that balance accuracy with computational efficiency. Although extensive testing of alternative electronic structure methods for the SHEF1552 set is reserved for future work, an initial comparison of interaction energies calculated during this work [CP-LMP2-F12, SAPT0, SAPT2, and CP-M06-2X-D3(0)] against the LCCSD(T*)-F12 benchmark was performed. The error in the interaction energy (ΔI.E.) is calculated as the benchmark value minus the comparison method value. Under this sign convention, positive values indicate that a method predicts that the interaction is stronger than the benchmark value (overbinding). Summary statistics for ΔI.E. across each method are presented in Table 1 and visualised as violin plots in Fig. S10.

**Table 1:** Summary statistics of the error in interaction energies, ΔI.E., (kcal mol^−1^) relative to the CP-LCCSD(T*)-F12/aug-cc-pVDZ-F12 benchmark data. Both the mean absolute error (MAE) and mean signed error (MSE) are reported

ΔI.E.	CP-LMP2-F12	SAPT2	SAPT0	CP-M06-2X-D3(0)
MAE	0.81	0.72	2.03	0.76
MSE	0.81	0.66	2.02	0.59
S.D.	0.61	0.91	2.07	0.85
Max	6.30	12.99	17.40	3.87
Min	−0.11	−1.02	−0.38	−2.10
Q1	0.38	0.11	0.66	−0.03
Q3	1.09	0.94	2.66	1.03

All four methods typically predict interactions stronger than those from the coupled-cluster benchmark (Table 1) and both SAPT truncations produce some energies with substantial errors (with SAPT0 having a maximum error of 17.40 kcal mol^−1^). The largest error for CP-LMP2-F12, SAPT2 and SAPT0 methods occurs for the NBS⋯PMe_3_ complex, demonstrating that SAPT methods do not perform well for interactions with significant covalent character. If this structure is removed, the maximum errors drop substantially to 3.96 kcal mol^−1^ for CP-LMP2-F12, 6.44 kcal mol^−1^ for SAPT2, and 16.80 kcal mol^−1^ for SAPT0.

SAPT2 markedly outperforms SAPT0 and approaches the overall accuracy of CP-LMP2-F12 in terms of error relative to the benchmark. Meanwhile, the performance of CP-M06-2X-D3(0) is broadly similar to CP-LMP2-F12, but with a slightly lower mean absolute error (0.76 kcal mol^−1^ compared to 0.81 kcal mol^−1^). Further analysis split by interaction class (Fig. S11–S14) shows that CP-M06-2X-D3(0) outperforms CP-LMP2-F12 for both halogen and chalcogen bonds, and has similar performance for pnictogen and tetrel bonds.

### 3.4 The nature of *σ*-hole interactions: energy partitioning by SAPT analysis

Symmetry-adapted perturbation theory (SAPT) provides an opportunity for further analysis of the nature of the different σ-hole interactions as it reveals how the interaction energy is constructed from electrostatic (*E*_elec_), exchange (*E*_exch_), induction (*E*_ind_), and dispersion (*E*_disp_) components. The reasonable agreement of the total SAPT2 interaction energy with the CP-LCCSD(T*)-F12 benchmark lends additional confidence to these energy components, although they should be used with caution for the strongest interactions with covalent character, such as the NBS⋯PMe_3_ complex (*vide supra*).

To classify the nature of the σ-hole interactions in SHEF1552, we adopted the approach of Gráfová *et al.*,^96^ which was originally designed for DFT-SAPT, based on the relative importance of the SAPT2 terms. Complexes are designated as:

• Electrostatic-dominated if |*E*_elec_| ≥ 2 × |*E*_disp_|.

• Dispersion-dominated if |*E*_disp_| ≥ 2 × |*E*_elec_|.

• Mixed-character if neither criterion is met.

This approach is illustrated in Fig. 10, which displays the CP-LCCSD(T*)-F12 interaction energy plotted against *d*_norm_, with each complex colour-coded by its SAPT classification. This classification loosely correlates with distance and strength as the 56 dispersion-dominated complexes tend to have longer *d*_norm_ and smaller interaction energies (Fig. S18), whereas the 214 electrostatic-dominated complexes are generally shorter and stronger (Fig. S16). The majority (1282 complexes) are mixed-character, sitting between these two extremes (Fig. S17). This mixed character is consistent with the IUPAC definition for halogen bonds “*The attractive nature of the interaction is mainly due to electrostatic effects, but polarization, charge-transfer, and dispersion contributions all play an important role in causing interpenetration of van der Waals volumes*”,^9^ which also holds for chalcogen and pnictogen bonds,^11,12^ and presumably any future definition of tetrel bonds.

**Fig. 10:**
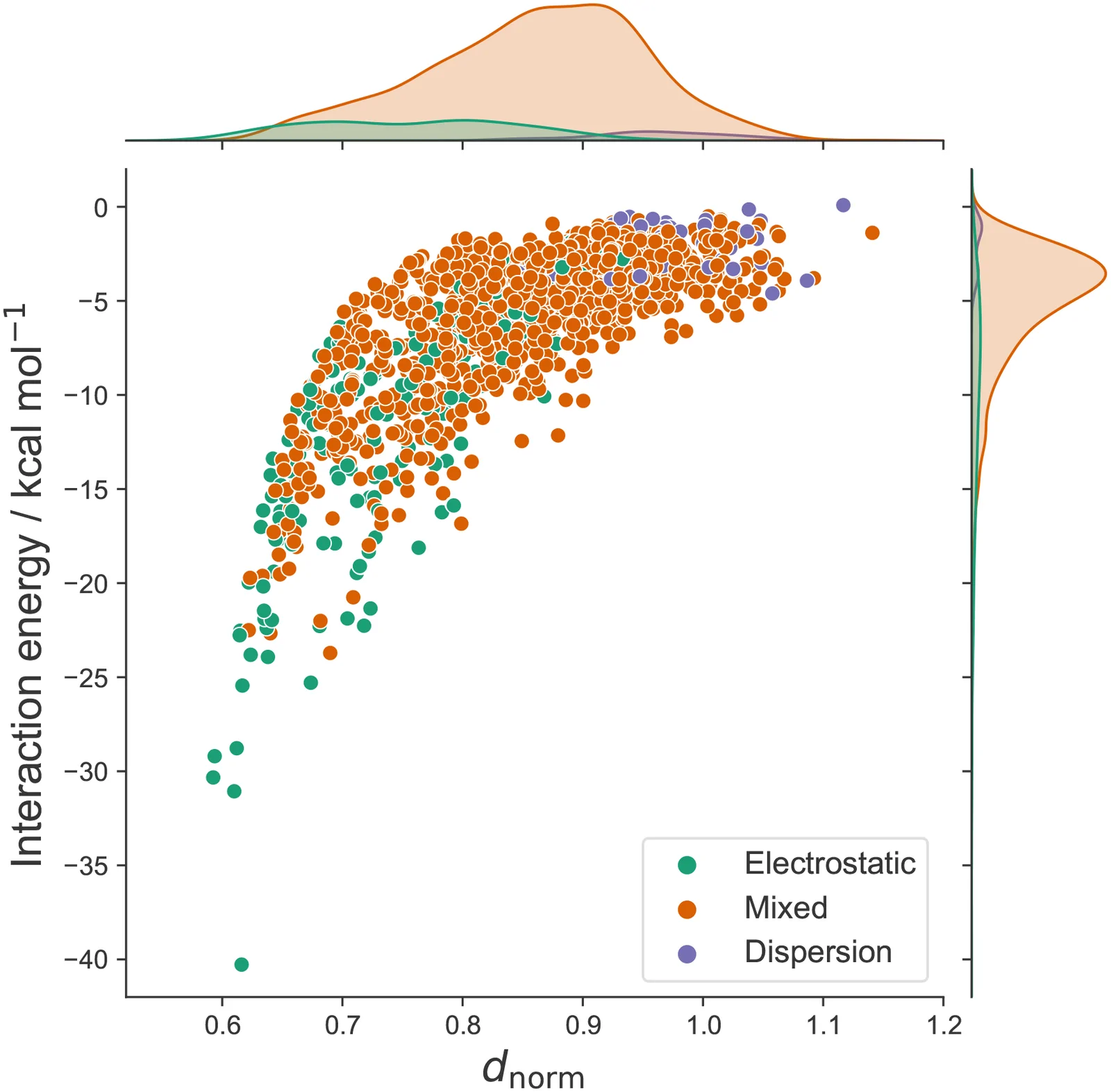
Counterpoise-corrected LCCSD(T*)-F12 interaction energies plotted against the normalised distance (*d*_norm_), with each point colour-coded as electrostatic-dominated, dispersion-dominated, or mixed-character interaction type, as determined by SAPT2 analysis. All geometries were optimised at the M06-2X-D3(0)/def2-TZVPD level of theory.

Although no interaction is dominated by the induction component, Fig. 11 confirms that stronger interactions exhibit a greater percentage contribution from induction. This is particularly true for complexes with sizeable covalent character, such as NBS⋯PMe_3_, although SAPT may be less reliable in cases where the interactions approach those of covalent chemical bonds.

**Fig. 11:**
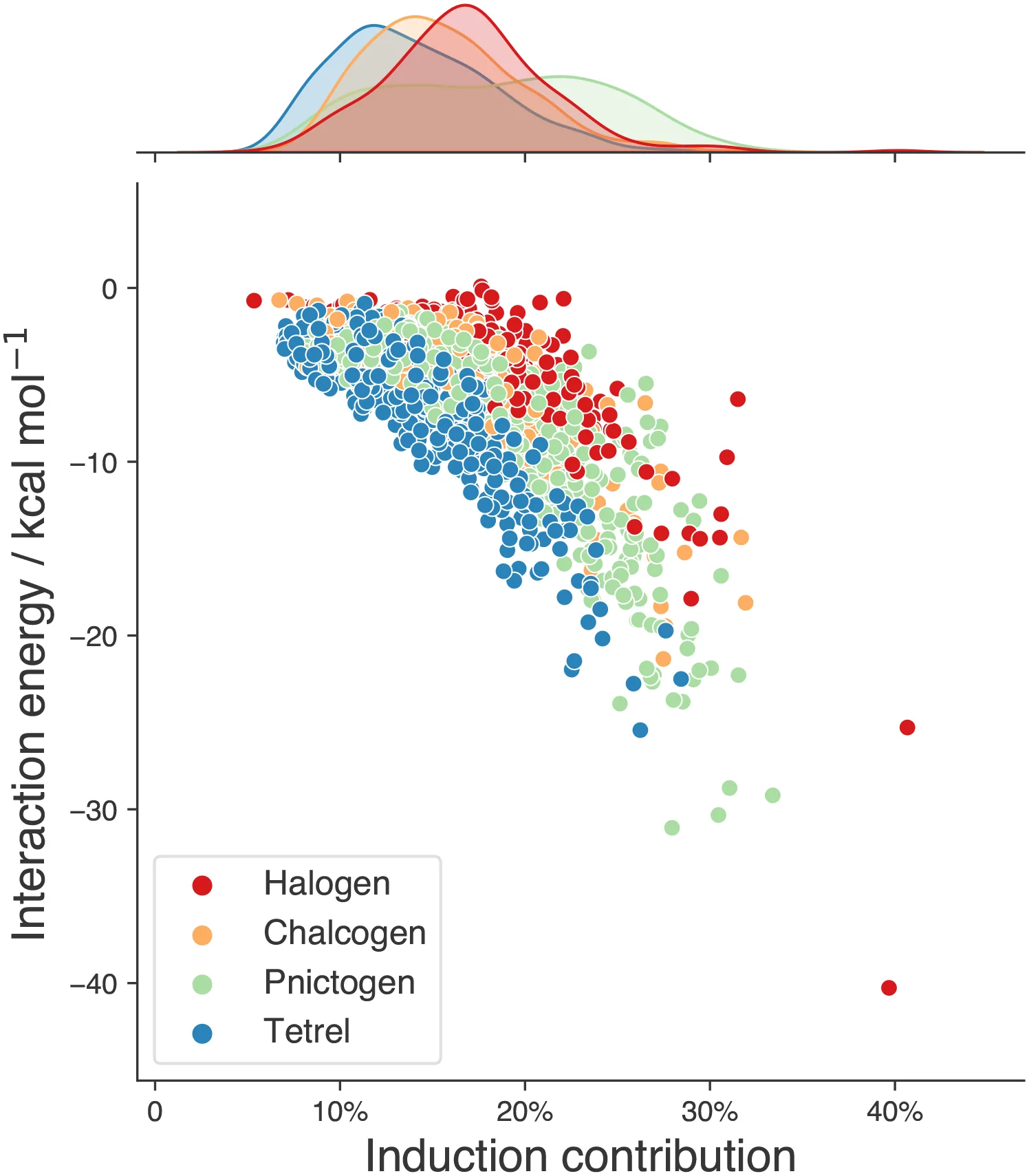
Counterpoise corrected LCCSD(T*)-F12 interaction energies plotted against the percentage contribution of the induction component to the total of the attractive SAPT terms. Also shown are kernel density estimate plots of the induction contributions grouped by interaction class.

The distribution of the induction contribution varies notably by Periodic Table group. Halogen bonds show slightly greater induction character than tetrel or chalcogen bonds. Pnictogen bond interactions are clearly bimodal, one peak has low induction character similar to tetrel bonds, while the other shows substantially greater induction contributions than any other σ-hole interaction class. This high induction character for a subset of pnictogen bonds suggests a greater degree of charge transfer/polarisation in these specific complexes, which are typically composed of σ-hole donors that contain multiple strongly electron withdrawing groups (*e.g.*, heavy pnictogen tricyanides) interacting with activated Lewis bases such as pyridine-*N*-oxide or PMe_3_.

Further analysis of the SAPT energy components is presented in the SI (Section S6.4), including breakdowns of the induction contribution to the classification types (Fig. S19–S21), and trends related to the dispersion component. The dispersion contribution is found to have a non-linear inverse relationship with interaction strength (Fig. S22), where pnictogen bonds generally show the smallest dispersion contribution. Tetrel bond interactions are bimodal in dispersion character, with one maximum close to that of chalcogen bonds and another for the weaker, dispersion-dominated interactions.

Taken together, the geometric parameters, high-accuracy interaction energies and SAPT components across the SHEF1552 dataset provides a continuous picture of σ-hole interactions that fundamentally moves beyond the limitations of common, distance-only based analysis.

## 4 Conclusions

This study introduces the SHEF1552 dataset, a compilation of 1552 intermolecular σ-hole interactions (halogen, chalcogen, pnictogen and tetrel bonds). The dataset spans a wide range of relevant chemical space, covering various σ-hole donor atoms interacting with a consistent set of 19 Lewis bases as the σ-hole acceptors. High-level PNO-LCCSD(T*)-F12b interaction energies were computed, complemented by a detailed SAPT analysis of the nature of these interactions.

Examination of this extensive dataset uncovered several key trends and provides valuable physical insights into σ-hole interactions:

1. Pnictogen bonds generally exhibit the shortest normalised interaction distance (*d*_norm_), followed by tetrel bonds, with halogen- and chalcogen bonds being the longest. Tetrel and halogen bonds are typically closest to linearity (*θ* ≈ 180°). In contrast, pnictogen bonds have an average σ-hole angle of ≈164° and uniquely display no clear relationship between *d*_norm_ and the σ-hole angle.

2. The interaction strength increases with the polarisability of the σ-hole donor atom within each group. Overall, pnictogen bonds are the strongest interactions, followed by tetrel, chalcogen, and finally halogen bonds. The relationship between energy and *d*_norm_ shows a distinct boomerang-shaped distribution, signifying a division between weaker and stronger interactions, the transition occurring around *d*_norm_ = 0.8.

3. The common practice of using *d*_norm_ ≤ 1.0 as a cut-off for intermolecular interactions is shown to be too crude. Approximately 5% of the structures in dataset display longer-range (*d*_norm_ > 1.0) interactions that nonetheless retain appreciable interaction energies (mean of −2.70 kcal mol^−1^). The *p*_AB_ index, which maps the interaction distance relative to both the covalent bond distance and the sum of the van der Waals radii of the interacting atoms, is demonstrated to be a superior distance-based descriptor for interaction strength.

4. In a comparison of electronic structure methods against our benchmark, CP-LMP2-F12, SAPT2, SAPT0, and CP-M06-2X-D3(0) generally overestimate the interaction energy. CP-M06-2X-D3(0) exhibited the lowest mean error (0.59 kcal mol^−1^), performing comparably to CP-LMP2-F12.

5. Classification of the interactions using SAPT2 energy partitioning terms reveals that the strongest are electrostatic-dominated, whereas the weakest are dispersion-dominated. The majority of interactions are mixed-character (that is, between these two extremes), consistent with IUPAC definitions of σ-hole interactions. Pnictogen bonds show a bimodal distribution of the induction component, indicating a greater degree of charge-transfer/polarisation in a subset of these complexes with the strongest interactions.

This comprehensive dataset opens numerous avenues for future research. The SHEF1552 data could serve as an invaluable resource for the extensive benchmarking and development of electronic structure methods relevant to non-covalent interactions. Furthermore, it is ideally suited for the training and validation of machine learning models, as we have recently demonstrated through our physics-informed model (σ-hat) for predicting σ-hole interaction energies.^97^ The dataset could also be extended to include additional descriptors from methods like quantum theory of atoms in molecules or the non-covalent interactions index.^98–100^ It is our hope that by making this dataset and its underlying geometries openly available, the broader chemical community will be able to devise new methodologies and insights, further expanding the applications of σ-hole interactions across the chemical sciences.

## Author contributions

H. W. N.: methodology, data curation, investigation. L. B.: conceptualisation, formal analysis, writing – review & editing, funding acquisition. J. G. H.: conceptualisation, methodology, data curation, investigation, formal analysis, writing – original draft, writing – review & editing, funding acquisition.

## Conflicts of interest

The authors have no conflicts to disclose.

## Supplementary Material

CP-OLF-D6CP04299F-s001

CP-OLF-D6CP04299F-s002

CP-OLF-D6CP04299F-s003

## Data Availability

The optimised geometries for all of the intermolecular complexes in the SHEF1552 datatset, in XYZ Cartesian coordinate format, are available *via* the University of Sheffield ORDA research data repository at https://doi.org/10.15131/shef.data.33269589. Supplementary information (SI): comprises a full description of the computational methods, listings of the σ-hole donor molecules, analysis of the deviation from linearity in pnictogen-bonding interactions, details of the CSD search construction, further analysis and visualisation of the interaction energies and SAPT components, and analysis of the performance of the PNO-LCCSD(T*)-F12b method for interaction energies of the intermolecular complexes in the SH250 dataset. A separate comma-separated values (CSV) file tabulating the σ-hole angle, *d*_norm_, interaction energies at several levels of theory, and other useful information for all complexes is also provided, along with machine readable files containing the auxiliary basis sets developed in this work (Molpro format). See DOI: https://doi.org/10.1039/d6cp04299f.
