# Supplementary material for: The SHEF1552 dataset: fundamental trends and physical insights into halogen, chalcogen, pnictogen, and tetrel bonds
Source: Phys Chem Chem Phys. 2026 Nov 6. Online ahead of print. doi: 10.1039/d6cp04299f (PMC13760327; doi:10.1039/d6cp04299f)
Supplement: Supplementary file 1 [file CP-OLF-D6CP04299F-s003.pdf]

Supplementary Information (SI) for Physical Chemistry Chemical Physics.
This journal is © the Owner Societies

Supporting Information: The SHEF1552 dataset: Fundamental trends and physical insights into halogen, chalcogen, pnictogen, and tetrel bonds

Harry W. Nash,[1] Lee Brammer,[1] and J. Grant Hill[1,2,*]

[1] *Chemistry Division, School of Mathematical and Physical Sciences, University of Sheffield, Sheffield S3 7HF, U.K.*

[2] *Centre for Machine Intelligence, University of Sheffield, Sheffield S10 2TN, U.K.*

[*] grant.hill@sheffield.ac.uk

CONTENTS

S1. The SHEF1552 dataset 3

S2. σ-hole donor molecules contained with the SHEF1552 dataset 4

S3. Technical details of electronic structure calculations 8

S3.1. DFT calculations 8

S3.2. Coupled-cluster calculations 8

S3.3. Symmetry-adapted perturbation theory 10

S3.4. MP2Fit auxiliary basis sets for H, B–Ne, and Al–Ar 10

S3.5. Orbital and auxiliary basis sets used in F12 calculations 12

S4. Complexes with imaginary frequencies 13

S5. Geometric Parameters 15

S5.1. Deviation from linearity in pnictogen-bonding interactions 19

S5.2. CSD search construction 21

S5.3. Comparison of σ-hole angles in SHEF1552 and the subset of CSD search results 23

S6. Interaction Energies 24

S6.1. Interaction energies subdivided into interaction classes 25

S6.2. Interpenetration of van der Waals crusts 28

S6.3. Interaction energies from other levels of theory: Errors relative to benchmark data 31

S6.4. SAPT Analysis 36

S6.5. Dispersion contributions to the SAPT interaction energy 43

S7. Performance of the PNO-LCCSD(T*)-F12b method for the SH250 dataset interaction energies 47

S8. References 51

S1. THE SHEF1552 DATASET

The dataset, which includes key geometrical parameters, energies, SAPT components, and other information for each complex, is provided as a machine readable CSV file.

The optimised geometries for each complex are available as a separate download (archive of machine readable XYZ coordinate files) from the University of Sheffield ORDA research data repository at `http://doi.org/10.15131/shef.data.33269589`. These files are zero-indexed, with the filename matching the "Fileindex" column within the CSV file.

S2. σ-HOLE DONOR MOLECULES CONTAINED WITH THE SHEF1552 DATASET

TABLE S1: Halogen bond donor molecules contained within the SHEF1552 σ-hole dataset.

Molecule name	Chemical formula	Short name used in CSV file
Trifluorochloromethane	CF_3Cl	cf3cl
Trifluorobromomethane	CF_3Br	cf3br
Trifluoroiodomethane	CF_3I	cf3i
Chloromethane	CH_3Cl	ch3cl
Bromomethane	CH_3Br	ch3br
Iodomethane	CH_3I	ch3i
Dichloroethyne	ClCCCl	clcccl
Dibromoethyne	BrCCBr	brccbr
Diiodoethyne	ICCI	icci
Chloroethyne	HCCCl	hcccl
Bromoethyne	HCCBr	hccbr
Iodoethyne	HCCI	hcci
Chlorobenzene	C_6H_5Cl	c6h5cl
Bromobenzene	C_6H_5Br	c6h5br
Iodobenzene	C_6H_5I	c6h5i
2,4,6-trifluorochlorobenzene	$C_6F_3H_2Cl$	c6f3h2cl
2,4,6-trifluorobromobenzene	$C_6F_3H_2Br$	c6f3h2br
2,4,6-trifluoroiodobenzene	$C_6F_3H_2I$	c6f3h2i
N-chlorosuccinimide	$C_2H_4(CO)_2NCl$	suc-cl
N-bromosuccinimide	$C_2H_4(CO)_2NBr$	suc-br
N-iodosuccinimide	$C_2H_4(CO)_2NI$	suc-i

TABLE S2: Chalcogen bond donor molecules contained within the SHEF1552 σ-hole dataset.

Molecule name	Chemical formula	Short name used in CSV file
Thiazole	C_3H_3NS	thiazole
Selenazole	C_3H_3NSe	selenazole
Tellurazole	C_3H_3NTe	tellurazole
Carbonyl sulfide	OCS	ocs
Carbonyl selenide	OCSe	ocse
Carbonyl telluride	OCTe	octe
Sulfur dicyanide	$(NC)_2S$	cn_2s
Selenium dicyanide	$(NC)_2Se$	cn_2se
Tellurium dicyanide	$(NC)_2Te$	cn_2te
Thiophene-3(2H)-one	C_4H_4OS	thiophene-3-one
Selenophene-3(2H)-one	C_4H_4OSe	selenophene-3-one
Tellerophene-3(2H)-one	C_4H_4OTe	tellerophene-3-one
Difluoro thioformaldehyde	F_2CS	f2cs
Difluoro selenoformaldehyde	F_2CSe	f2cse
Difluoro telleroformaldehyde	F_2CTe	f2cte
Methyl thiocyanate	$CN(CH_3)S$	ncmes
Methyl selenocyanate	$CN(CH_3)Se$	ncmese
Methyl tellerocyanate	$CN(CH_3)Te$	ncmete
Dimethlyammino methanethiol	$(CH_3)_2NSCH_3$	nme_2mes
Dimethlyammino methaneselenol	$(CH_3)_2NSeCH_3$	nme_2mese
Dimethlyammino methanetellerol	$(CH_3)_2NTeCH_3$	nme_2mete

TABLE S3: Pnictogen bond donor molecules contained within the SHEF1552 σ-hole dataset.

Molecule name	Chemical formula	Short name used in CSV file
Phosphorus tricyanide	$P(CN)_3$	cn_3p
Arsenic tricyanide	$As(CN)_3$	cn_3as
Antimony tricyanide	$Sb(CN)_3$	cn_3sb
Bismuth tricyanide	$Bi(CN)_3$	cn_3bi
Fluorophosphine	FH_2P	fh2p
Fluoroarsane	FH_2As	fh2as
Fluorostibane	FH_2Sb	fh2sb
Fluorobismuthane	FH_2Bi	fh2bi
Trimethylphosphine	$P(CH_3)_3$	me3p
Trimethylarsane	$As(CH_3)_3$	me3as
Trimethylstibane	$Sb(CH_3)_3$	me3sb
Trimethylbismuthane	$Bi(CH_3)_3$	me3bi
Methylphosphine dicyanide	$(CN)_2PCH_3$	cn_2mep
Methylarsane dicyanide	$(CN)_2AsCH_3$	cn_2meas
Methylstibane dicyanide	$(CN)_2SbCH_3$	cn_2mesb
Methylbismuthane dicyanide	$(CN)_2BiCH_3$	cn_2mebi
Dimethylamino dimethylphosphine	$(CH_3)_2NP(CH_3)_2$	nme_2me_2p
Dimethylamino dimethylarsane	$(CH_3)_2NAs(CH_3)_2$	nme_2me_2as
Dimethylamino dimethylstibane	$(CH_3)_2NSb(CH_3)_2$	nme_2me_2sb
Dimethylamino dimethylbismuthane	$(CH_3)_2NBi(CH_3)_2$	nme_2me_2bi

TABLE S4: Tetrel bond donor molecules contained within the SHEF1552 σ-hole dataset.

Molecule name	Chemical formula	Short name used in CSV file
Dicyanodimethylsilane	$(CN)_2Si(CH_3)_2$	me_2cn_2si
Dicyanodimethylgermane	$(CN)_2Ge(CH_3)_2$	me_2cn_2ge
Dicyanodimethylstannane	$(CN)_2Sn(CH_3)_2$	me_2cn_2sn
Dicyanodimethylplumbane	$(CN)_2Pb(CH_3)_2$	me_2cn_2pb
Tetramethylsilane	$Si(CH_3)_4$	me_4si
Tetramethylgermane	$Ge(CH_3)_4$	me_4ge
Tetramethylstannane	$Sn(CH_3)_4$	me_4sn
Tetramethylplumbane	$Pb(CH_3)_4$	me_4pb
Dichlorodimethylsilane	$(CH_3)_2Si(Cl)_2$	me_2cl_2si
Dichlorodimethylgermane	$(CH_3)_2Ge(Cl)_2$	me_2cl_2ge
Dichlorodimethylstannane	$(CH_3)_2Sn(Cl)_2$	me_2cl_2sn
Dichlorodimethylplumbane	$(CH_3)_2Pb(Cl)_2$	me_2cl_2pb
Trimethylsilanecarbonitrile	$(CH_3)_3SiCN$	me_3cnsi
Trimethylgermanecarbonitrile	$(CH_3)_3GeCN$	me_3cnge
Trimethylstannanecarbonitrile	$(CH_3)_3SnCN$	me_3cnsn
Trimethylplumbanecarbonitrile	$(CH_3)_3PbCN$	me_3cnpb
N-trimethylsilane succinimide	$(CH_3)_3Si(C_2H_4(CO)_2N)$	sucme_3si
N-trimethylgermane succinimide	$(CH_3)_3Ge(C_2H_4(CO)_2N)$	sucme_3ge
N-trimethylstannane succinimide	$(CH_3)_3Sn(C_2H_4(CO)_2N)$	sucme_3sn
N-trimethylplumbane succinimide	$(CH_3)_3Pb(C_2H_4(CO)_2N)$	sucme_3pb

S3. TECHNICAL DETAILS OF ELECTRONIC STRUCTURE CALCULATIONS

No attempt was made to incorporate the effects of spin-orbit coupling into the calculation of interaction energies or within geometry optimisations. Although "correcting" the interaction energies may be possible by using some form of composite computational scheme, accounting for this within the geometries of all complexes contained within this dataset, at a suitable level of theory, is far beyond current, pragmatic computational power.

S3.1. DFT calculations

The def2-TZVPD basis set used in the DFT calculations is a polarised triple-zeta basis that includes additional diffuse functions and small-core, relativistic effective core potentials (ECPs) that replace 28 electrons for Sn, Sb, Te, and I, and 60 electrons for Pb and Bi.[1–4]

All geometry optimisations were carried out in ORCA version 5.0.4,[5] with tight SCF conversion criteria and the defgrid3 integration grid.[5] Density fitting was used in the evaluation of the Coulomb integrals, using the 'universal' def2 auxiliary Coulomb basis.[6] Structures possessing imaginary frequencies were distorted along the associated normal mode(s) using a Python script that makes use of the cclib (version 1.8.1) library to parse ORCA output.[7,8] The resulting structures were then re-optimised.

S3.2. Coupled-cluster calculations

The explicitly correlated local coupled-cluster methodology was used to produce interaction energies at the DFT optimised geometries. More specifically, the PNO-LCCSD(T)-F12b method that uses pair natural orbitals was used,[9–11] where the local correlation approach addresses the steep scaling of coupled-cluster methods, and explicit correlation (F12) both accelerates convergence with respect to basis set size and partly corrects the errors introduced by the domain approximation in local correlation.[12] The use of approximation b in these F12 calculations is implicit herein. For elements from the first two rows of the Periodic Table, the aug-cc-pVDZ-F12 basis set was used,[13,14] whereas cc-pVDZ-F12 was used for hydrogen to prevent the basis set becoming linearly dependent. The aug-cc-pVDZ-PP-F12 basis set was used for the heavier p-block elements,[15,16] with the ECP10MDF ECP for $4p$ elements, ECP28MDF for $5p$, and ECP60MDF for $6p$.[3,4,17,18] Herein, this mixed basis

set shall be referred to as aVDZ-F12. It is notable that although this PNO-LCCSD(T)-F12/aVDZ-F12 level of theory only employs a double-zeta basis set, for the S66 benchmark set of Řezáč, Riley and Hobza,[19,20] it produces interaction energies with a root-mean-square deviation (RMSD) of only 0.05 kcal mol^{-1} and a maximum deviation of 0.11 kcal mol^{-1},[21] making it an accurate and efficient choice for the relatively large number of complexes in the present investigation. All coupled-cluster calculations were carried out in the Molpro package of *ab initio* programs, version 2023.2.[22,23]

F12 calculations require three different types of auxiliary basis set (ABS) for integral evaluations. In the following, the Molpro notation for ABSs is used, where text after a forward slash denotes the context of the type of intergrals approximated. The density-fitted Hartree-Fock calculations used the aug-cc-pV(T+d)Z/JKFit ABS for lighter elements,[24,25] and def2-AQZVPP/JKFit for 4p, 5p, and 6p elements.[26] In both cases, the exponents of the additional diffuse functions were determined by an even-tempered extension. The complementary auxiliary basis set (CABS) singles correction was added to the Hartree-Fock energies,[27] and this was evaluated using the cc-pVDZ-F12+/OptRI ABS for lighter elements,[28] and aug-cc-pVDZ-PP-F12/OptRI for the heavier p-block.[16] These CABS singles corrections were evaluated without local approximations. All of the remaining two-electron integrals in the PNO-LCCSD(T)-F12 calculations used the aug-cc-pVDZ(-PP)-F12/MP2Fit ABSs,[16,29] with local density fitting.[11,30] For the lighter elements, the aug-cc-pVDZ-F12/MP2Fit sets were produced by even-tempered extension of the cc-pVDZ-F12/MP2Fit sets with additional diffuse functions, and further details are provided below. The resolution-of-the-identity (RI) ABS used for the many-electron integrals that arise in F12 theory was the union of the orbital basis with aug-cc-pV(T+d)Z/JKFit for the lighter elements and def2-AQZVPP/JKFit for the heavier p-block. For ease of reference, Table S5 details which basis sets, orbital and auxiliary, are used for each element.

As the perturbative triples in PNO-LCCSD(T)-F12 are not directly affected by the explicit correlation, their convergence with respect to basis set size can be much slower than the singles and doubles. To address this, the triples contribution was scaled by the ratio between the MP2-F12 correlation energy and the conventional MP2 correlation energy, which is commonly known as the (T*) energy.[31] To retain size consistency, the scaling factor was determined for each dimer complex and then held fixed for the respective monomers.[32] Tight domains, as defined by Ma and Werner,[12] were used in the PNO-LCCSD(T)-F12

calculations, along with the 3*A(FIX) *Ansatz* and F12b approximation.[33,34]

In the context of halogen and chalcogen bonding, it has been shown that $(n-1)d$ subvalence correlation is important for the calculation of accurate interaction energies with the heavier p-block elements.[35,36] Herein, all coupled-cluster calculations include the $(n-1)d$ electrons in the correlation treatment, and the aug-cc-pVDZ-PP-F12 basis set does include functions specifically designed for this form of correlation. To provide a balance between valence-valence, core-valence and core-core correlation, pair specific geminal exponents are used in the F12 treatment for complexes containing the heavy p-block elements.[37] A geminal exponent of 1.0 a_0^{-1} was used for the valence-valence pairs, and 1.4 a_0^{-1} for all other pairs.[38]

Interaction energies at the following levels of theory, both with and without CP correction, are extracted from the explicitly correlated local coupled-cluster calculations: Hartree-Fock with the CABS singles correction (HF+CABS), LMP2-F12, PNO-LCCSD(T)-F12b [further abbreviated as LCCSD(T)-F12], and PNO-LCCSD(T*)-F12b [LCCSD(T*)-F12].

S3.3. Symmetry-adapted perturbation theory

SAPT2 calculations were carried out using the PSI4 program package version 1.8.2,[39] with the def2-TZVPD basis set for all heavy atoms, and def2-TZVP for hydrogens. For the complexes $C_6H_5I\cdots NCCH_3$ and $Me_2N(Me)_2Sb\cdots$dioxane the diffuse functions caused SCF convergence problems related to linear dependency, hence the def2-TZVP basis was used for all atoms for these two complexes. Density fitting in the HF parts of the calculation use the def2-QZVPP/JKFit ABS,[26] while the density fitting of the intramonomer correlation corrections use the def2-TZVP(D)/MP2Fit ABS.[40–43] We note that SAPT0 energies are also provided as part of the output of a SAPT2 calculation.

S3.4. MP2Fit auxiliary basis sets for H, B–Ne, and Al–Ar

Although cc-pVnZ-F12/MP2Fit auxiliary sets have been developed for the lighter elements (H, B—Ne, and Al–Ar),[29] these have not been designed for density fitting with the diffuse-augmented aug-cc-pVnZ-F12 orbital sets.[14] To reduce the density fitting error in such cases, the cc-pVnZ-F12/MP2Fit sets have been adapted following a procedure analogous to that adopted in aug-cc-pVnZ-PP-F12/MP2Fit sets for the heavy p-block elements.[16] This

entails an even-tempered extension of the existing exponents in specific angular shells; if ζ_1^l and ζ_2^l are the lowest value and second lowest value exponents in given shell l, respectively, then the new diffuse exponent is defined as $\zeta_{\text{diffuse}}^l = \zeta_1^l/(\zeta_2^l/\zeta_1^l)$.

The previous work on the heavier p-block elements confirmed that only a subset of the higher-angular shells require this augmentation, and a similar approach is again adopted here. After initial testing to determine which angular shells had the largest impact, at the DZ level, the H ABS is augmented with an additional d exponent, while those for B–Ne and Al–Ar add a single additional f exponent for each element. TZ adds d and f exponents for H, and f and g for B–Ne and Al–Ar. Finally, QZ adds d, f and g exponents for H, and f, g and h for B–Ne and Al–Ar.

The resulting ABS exponents are provided as machine readable text files in Molpro format.

S3.5. Orbital and auxiliary basis sets used in F12 calculations

TABLE S5: Orbital basis set (OBS) and auxiliary basis sets (ABSs) used for each element in PNO-LCCSD(T)-F12b calculations. References for each basis set are provided in the main text. The (aug-)cc-pVnZ stem of the basis sets is abbreviated as (a)VnZ.

Element(s)	OBS	JKFit ABS	MP2Fit ABS	RI ABS
H	aVDZ-F12	aVTZ/JKFit	aVDZ-F12/MP2Fit	VDZ-F12+/OptRI
C–F	aVDZ-F12	aVTZ/JKFit	aVDZ-F12/MP2Fit	VDZ-F12+/OptRI
Si–Cl	aVDZ-F12	aV(T+d)Z/JKFit	aVDZ-F12/MP2Fit	VDZ-F12+/OptRI
Ge–Br	aVDZ-PP-F12	def2-AQZVPP/JKFit	aVDZ-PP-F12/MP2Fit	aVDZ-PP-F12/OptRI
Sn–I	aVDZ-PP-F12	def2-AQZVPP/JKFit	aVDZ-PP-F12/MP2Fit	aVDZ-PP-F12/OptRI
Pb & Bi	aVDZ-PP-F12	def2-AQZVPP/JKFit	aVDZ-PP-F12/MP2Fit	aVDZ-PP-F12/OptRI

S4. COMPLEXES WITH IMAGINARY FREQUENCIES

Each stationary point located in the geometry optimisations underwent an analysis of the harmonic vibrational frequencies, again at the M06-2X-D3(0)/def2-TZVPD level of theory, to determine if the a true minimum had been located. After several cycles of distortion along normal modes associated with imaginary frequencies and subsequent geometry optimisations, 34 structures still possessed one or more low frequency imaginary modes. These complexes are listed in Table S6. The remaining imaginary modes were generally associated with propeller-like rotation of methyl (or similar) groups not involved in the intermolecular interaction. The $CH_3Br\cdots OH_2$ complex (file index 86.xyz) was removed from the final dataset of 1552 complexes as the optimised geometry does not correspond to a σ-hole interaction. A further five structures, which did not possess imaginary modes, were also excluded (further details in Section S5).

TABLE S6: Complexes with optimised geometries that possess one or more low frequency imaginary modes.

Complex	File index	Complex	File index
$CF_3I\cdots P(CH_3)_3$	14.xyz	$(CN)_2Pb(CH_3)_2\cdots SP(CH_3)_3$	1060.xyz
$CF_3I\cdots OP(CH_3)_3$	16.xyz	$(CH_3)_4Sn\cdots N(NH)(CH)_3$	1115.xyz
$CF_3Cl\cdots SC_4H_4$	50.xyz	$(CH_3)_4Pb\cdots PH_3$	1122.xyz
$CH_3Br\cdots OH_2$	86.xyz	$(CH_3)_4Pb\cdots NCH$	1123.xyz
$CH_3Cl\cdots OH_2$	105.xyz	$(CH_3)_4Pb\cdots N(CH_3)_3$	1126.xyz
$C_6H_5Cl\cdots PH_3$	267.xyz	$(CH_3)_4Pb\cdots OH_2$	1131.xyz
$C_6H_5Cl\cdots OH_2$	276.xyz	$(CH_3)_2Sn(Cl)_2\cdots ONC_5H_5$	1196.xyz
$C_6H_5Cl\cdots SC_4H_4$	278.xyz	$(CH_3)_3PbCN\cdots NCH$	1275.xyz
$C_6F_3H_2I\cdots SC_4H_4$	297.xyz	$(CH_3)_3PbCN\cdots ONC_5H_5$	1291.xyz
$C_6F_3H_2Cl\cdots P(CH_3)_3$	337.xyz	$C_2H_4(CO)_2NBr\cdots NCCH_3$	1322.xyz
$F_2CS\cdots PH_3$	571.xyz	$(CH_3)_2NBi(CH_3)_2\cdots NCCH_3$	1474.xyz
$F_2CSe\cdots PH_3$	590.xyz	$(CH_3)_3Si(C_2H_4(CO)_2N)\cdots NCCH_3$	1493.xyz
$F_2CSe\cdots SC_4H_4$	601.xyz	$(CH_3)_3Si(C_2H_4(CO)_2N)\cdots SP(CH_3)_3$	1497.xyz
$F_2CTe\cdots PH_3$	609.xyz	$(CH_3)_3Ge(C_2H_4(CO)_2N)\cdots NH_2C_6H_5$	1507.xyz
$CN(CH_3)Te\cdots NCCH_3$	676.xyz	$(CH_3)_3Ge(C_2H_4(CO)_2N)\cdots NCCH_3$	1512.xyz
$FH_2Bi\cdots NCCH_3$	828.xyz	$(CH_3)_3Pb(C_2H_4(CO)_2N)\cdots SC(CH_3)_2$	1543.xyz
$(CN)_2Sn(CH_3)_2\cdots NCCH_3$	1037.xyz	$(CH_3)_3Pb(C_2H_4(CO)_2N)\cdots NCCH_3$	1550.xyz

S5. GEOMETRIC PARAMETERS

Analysis of the optimised geometries revealed that five structures had σ-hole angles that were surprisingly small ($< 130°$), and one structure had an exceptionally long normalised distance ($d_{\rm norm} = 1.21$). Visual inspection of the geometries indicated that these complexes were not interacting via a σ-hole and, after attempts to re-optimize the geometries led to the same minima, these complexes were removed from the dataset. Further information on the composition and geometric parameters of these complexes can be found in Table S7. An additional two complexes were found to have repulsive interaction energies at the CP-M06-2X-D3(0)/def2-TZVPD level of theory; the $CH_3Cl\cdots NCH$ interaction energy is $+0.21$ kcal mol^{-1} and $CH_3Cl\cdots O_2NMe$ is $+0.16$ kcal mol^{-1}. At the CP-LCCSD(T*)-F12 level, the $CH_3Cl\cdots O_2NMe$ interaction becomes very weakly attractive at -0.14 kcal mol^{-1}, whereas $CH_3Cl\cdots NCH$ remains slightly repulsive at $+0.09$ kcal mol^{-1}. For the latter case, constrained potential energy scans along the internuclear distance coordinate indicate that there is some form of meta-stable minimum at the geometry presented within the dataset. As the data set should contain examples of σ-hole interactions that span as full a range of interaction strengths as practicable, the decision was taken to retain both of these complexes.

TABLE S7: Details of the six structures removed from the dataset as the complexes do not appear to be interacting via a σ-hole interaction.

Complex	File index	$d_{\rm norm}$	σ-hole angle (degrees)
$CH_3Br\cdots OH_2$	86.xyz	1.0864	119.2
$(CH_3)_3P\cdots NH_2C_6H_5$	842.xyz	1.0165	121.1
$(CH_3)_2NSCH_3\cdots PH_3$	1350.xyz	0.9314	120.8
$(CH_3)_2NSCH_3\cdots OC(CH_3)_2$	1352.xyz	1.0860	111.8
$(CH_3)_2NSCH_3\cdots O_2NCH_3$	1366.xyz	1.0207	126.7
$(CH_3)_2NP(CH_3)_2\cdots SP(CH_3)_3$	1421.xyz	1.2094	148.0

The summary statistics for the geometric parameters $d_{\rm norm}$ and θ are presented in Table S8, where the SHEF1552 dataset has been grouped by interaction class. Discussion of these data can be found in Section 3.1 of the main text. The normalised distances and σ-hole angles are further broken down by σ-hole donor atom in Tables S9 and S10, respectively.

TABLE S8: Summary statistics of the normalised distances (d_{norm}) and σ-hole angles (in degrees) from M06-2X-D3(0)/def2-TZVPD optimised geometries grouped by interaction class

d_{norm}	Tetrel	Pnictogen	Chalcogen	Halogen
Mean	0.8268	0.8049	0.8923	0.8715
S.D.	0.1127	0.1114	0.0632	0.0647
Min	0.6145	0.5925	0.7120	0.6161
Max	1.0865	1.0922	1.1409	1.1167
Q1	0.7333	0.7163	0.8556	0.8337
Q3	0.9212	0.8797	0.9352	0.9120
σ-hole angle	Tetrel	Pnictogen	Chalcogen	Halogen
Mean	174.4	164.4	168.1	175.2
S.D.	5.3	5.2	8.7	6.2
Min	152.0	151.3	141.8	138.5
Max	180.0	179.0	180.0	180.0
Q1	172.5	160.9	161.7	173.4
Q3	178.2	167.4	174.4	179.9

TABLE S9: Summary statistics of the normalised distances (d_{norm}) from M06-2X-D3(0)/def2-TZVPD optimised geometries grouped by interaction class and σ-hole donor atom.

Class	Donor atom	Mean	S.D.	Min	Max	Q1	Q3
Halogen	Cl	0.9029	0.0622	0.7441	1.1167	0.8622	0.9396
	Br	0.8777	0.0570	0.6161	1.0262	0.8461	0.9170
	I	0.8339	0.0550	0.6737	0.9408	0.8004	0.8690
Chalcogen	S	0.9082	0.0564	0.7512	1.1409	0.8765	0.9399
	Se	0.9163	0.0541	0.7479	1.0352	0.8822	0.9525
	Te	0.8528	0.0595	0.7120	1.0613	0.8168	0.8926
Pnictogen	P	0.8788	0.1037	0.6671	1.0922	0.7960	0.9573
	As	0.8776	0.0926	0.7042	1.0448	0.7988	0.9496
	Sb	0.7369	0.0747	0.5925	0.8995	0.6814	0.7958
	Bi	0.7278	0.0605	0.6100	0.8561	0.6799	0.7712
Tetrel	Si	0.9507	0.0700	0.7537	1.0865	0.9154	1.0020
	Ge	0.8704	0.0715	0.7150	1.0180	0.8103	0.9242
	Sn	0.7503	0.0754	0.6145	0.9521	0.6934	0.8086
	Pb	0.7360	0.0623	0.6347	0.9054	0.6879	0.7747

TABLE S10: Summary statistics of the σ-hole angles (in degrees) from M06-2X-D3(0)/def2-TZVPD optimized geometries grouped by interaction class and σ-hole donor atom.

Class	Donor atom	Mean	S.D.	Min	Max	Q1	Q3
Halogen	Cl	173.0	7.7	138.5	180.0	169.4	179.2
	Br	175.4	5.9	141.6	180.0	173.5	179.9
	I	177.4	3.6	160.7	180.0	176.1	180.0
Chalcogen	S	169.3	8.1	141.8	180.0	164.9	174.5
	Se	168.2	8.2	142.3	180.0	161.2	174.4
	Te	166.7	9.5	147.5	180.0	157.1	174.4
Pnictogen	P	168.5	4.3	157.2	179.0	165.5	170.9
	As	166.2	4.4	158.6	176.7	163.2	168.7
	Sb	161.8	4.2	152.5	173.6	159.2	164.0
	Bi	161.1	3.9	151.3	169.5	163.2	168.7
Tetrel	Si	174.1	5.9	152.6	180.0	171.3	178.5
	Ge	174.1	5.7	152.0	180.0	172.1	178.2
	Sn	174.9	4.2	154.9	179.9	173.0	177.9
	Pb	174.5	5.2	152.1	179.6	173.3	178.1

S5.1. Deviation from linearity in pnictogen-bonding interactions

Within the main text (Section 3.2 and Figure 7), an argument based upon VSEPR is made to rationalise the narrow range of σ-hole angles observed for pnictogen-bond interactions in this dataset. This is based upon a Lewis base approaching along the σ-hole axis encountering repulsion from the pnictogen lone pair, resulting in a deviation of the σ-hole angle from linearity. Some evidence for this positioning "away" from the lone pair is found from the optimised geometric parameters by calculating the value of the deviation from linearity.

$$\text{Deviation angle} = 180^\circ - \bar{\theta}_{\text{is}} - \bar{\theta}_{\text{sub}}$$

where $\bar{\theta}_{\text{is}}$ is the average of the inter-substituent angles [bond between the electron withdrawing group to pnictogen makes an angle with the bond between the pnictogen and a given ligand substituent, see Figure S1(a)], and $\bar{\theta}_{\text{sub}}$ is the average of the angle the bond between the pnictogen and a given substituent makes to the pnictogen bond [Figure S1(b)]. As such, a positive value of the deviation angle indicates that the Lewis base is located on the side towards the ligand substituents, whereas a negative value indicates the Lewis base is on the side towards the lone pair.

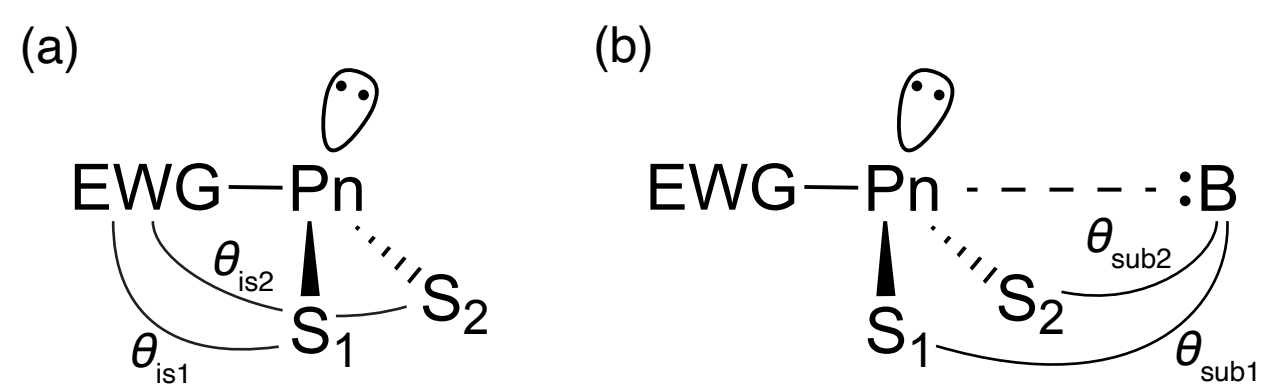

FIG. S1: Definition of the angles used to calculate the deviation from linearity when a pnictogen bond donor molecule interacts with a Lewis base, B. The primary (electron withdrawing) substituent is denoted EWG, whereas S_1 and S_2 represent other substituents. (a) Shows the two inter-substituent angles, θ_{is1} and θ_{is12}, which are averaged to find $\bar{\theta}_{\text{is}}$. (b) Shows the two angles formed from the substituents to the intermolecular pnictogen bond, θ_{sub1} and θ_{sub2}, which are averaged to find $\bar{\theta}_{\text{sub}}$.

Figure S2 shows that the majority of the deviation angles fall within a relatively small range, consistent with the overall pnictogen bond σ-hole angles discussed in the main text. Of the 378 pnictogen bonding interactions, only nine possess negative deviation angles. Thus, in general, the Lewis base will be positioned towards the substituents and away from the

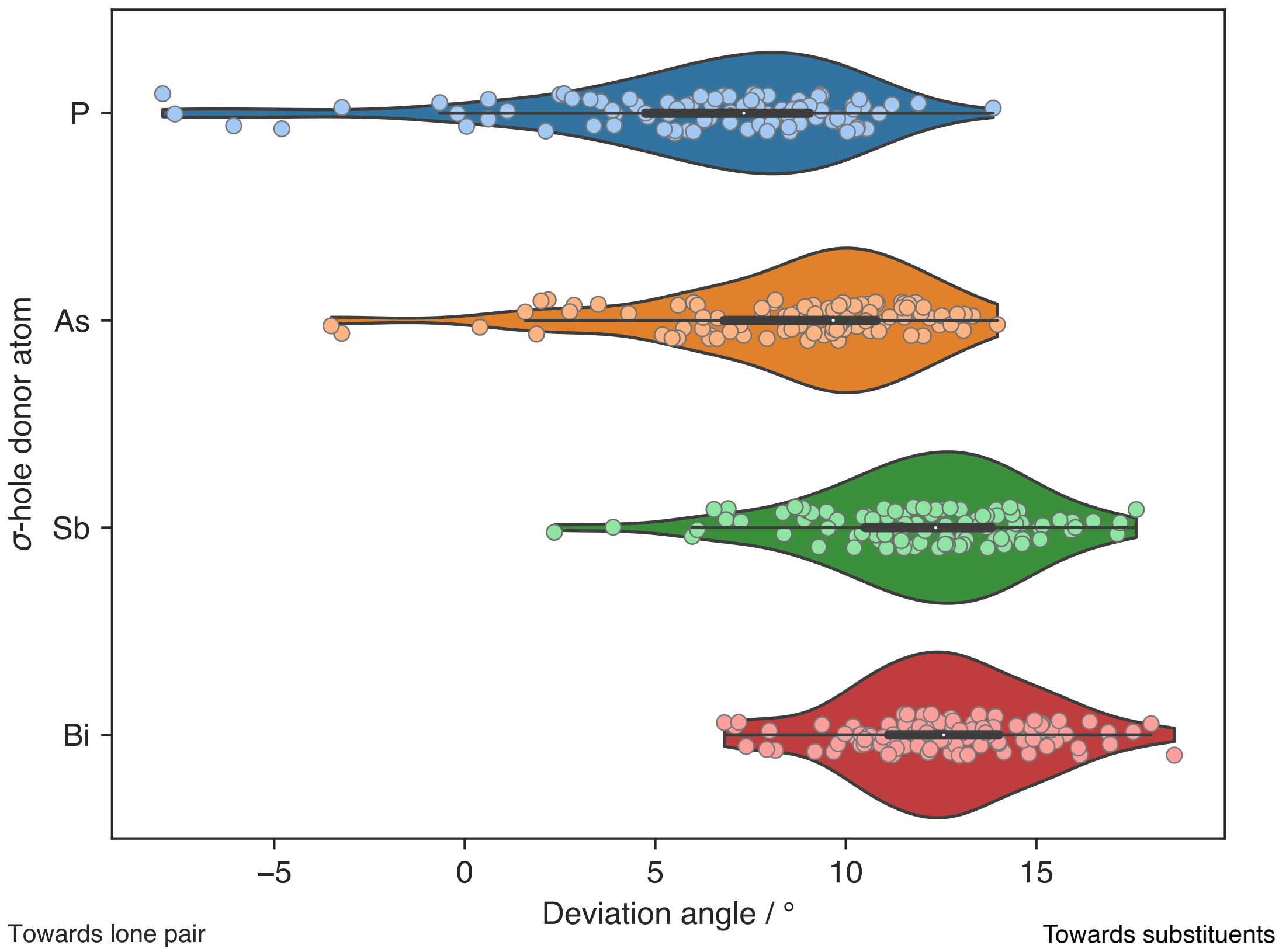

FIG. S2: Violin plots of the deviation angle for all pnictogen bond interactions in the SHEF1552 dataset. Angles for individual intermolecular complexes are shown as circle markers, with small amounts of jitter applied to reduce the overlap of markers.

pnictogen lone pair. All of the complexes with negative deviation angles have two methyl group substituents causing steric repulsion, and are weaker pnictogen-bond interactions involving P or As.

S5.2. CSD search construction

Searches were performed using the 2024.3 release of the CSD software suite, with database version 5.46 (November 2024) using a combination of ConQuest query files and version 3.3.0 of the CSD Python API.[44–46] The following restrictions were placed on search criteria: (i) crystallographic R-factor < 0.1; (ii) entry contains no errors; (iii) 3D atomic coordinates have been determined; (iv) no disorder reported; (v) structure is not polymeric. Any structures that have identical geometric measurements yet different CSD Refcodes were also removed to prevent duplicate data.

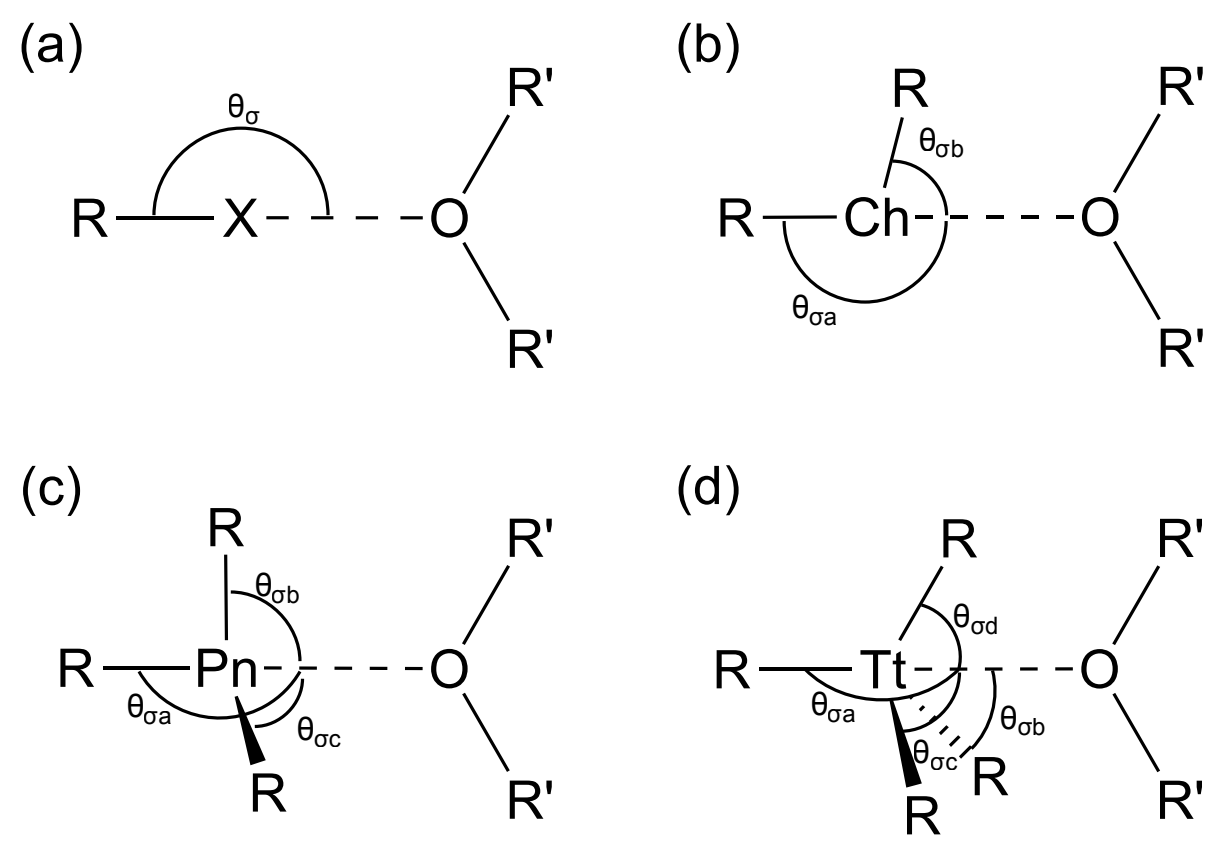

FIG. S3: A representation of the queries used to find (a) halogen [X] bonding, (b) chalcogen [Ch] bonding, (c) pnictogen [Pn] bonding, (d) tetrel [Tt] bonding contacts to divalent oxygen moieties within the CSD. θ_σ and $\theta_{\sigma\mathrm{a-d}}$ refer to the potential σ-hole angles, with the largest of these angles taken as the σ-hole angle. R = B, C, N, O, Si, P, S, F, Cl, Br, I, Se, Te. R′ = H, B, C, N, O, Si, P, S, F, Cl, Br, I, Se, Te. Allowed "bond types" for O−R′ within the searches were single, double, and delocalised.

Figure S3 illustrates the queries to find close contacts within the CSD that are indicative of σ-hole interactions to so-called "divalent oxygens" that take the form R′−O−R′. This divalent oxygen pseudo-functional group describes a wide range of potential σ-hole acceptors, including but not limited to ethers, alcohols, water, furans, and esters. A close contact is defined in the query as any distance between the halogen, chalcogen, pinictogen, or tetrel atom and the oxygen atom which is less than the sum of the van der Waals radii plus 0.2 Å. Each of the σ-hole donors have a number of singly bonded substituents (R) equal to their saturated valency (R = B, C, N, O, Si, P, S, F, Cl, Br, I, Se, Te). The angle each

R substituent makes with the σ-hole donor atom and the divalent oxygen is measured as a potential σ-hole angle, which for the halogen bonding systems is the actual σ-hole angle and is restricted in the searches to be between 110–180°. For the systems with multiple potential σ-hole angles the angle closest to linearity is selected as the σ-hole angle, provided it is greater than 110°. The oxygen atom has two R′ substituents (R′ = H, B, C, N, O, Si, P, S, F, Cl, Br, I, Se, Te) with allowance for CSD bond types of single bond, double bond or delocalised bond, and is restricted to a valency of two.

The searches described above resulted in 14,264 "hits", with a scatterplot showing the $d_{\rm norm}$ and σ-hole angle from these interactions presented in Figure S4(a). Applying filters of $d_{\rm norm} \leqslant 0.93$ and σ-hole angle $\geqslant 150°$ results in 1,541 hits [Figure S4(b)].

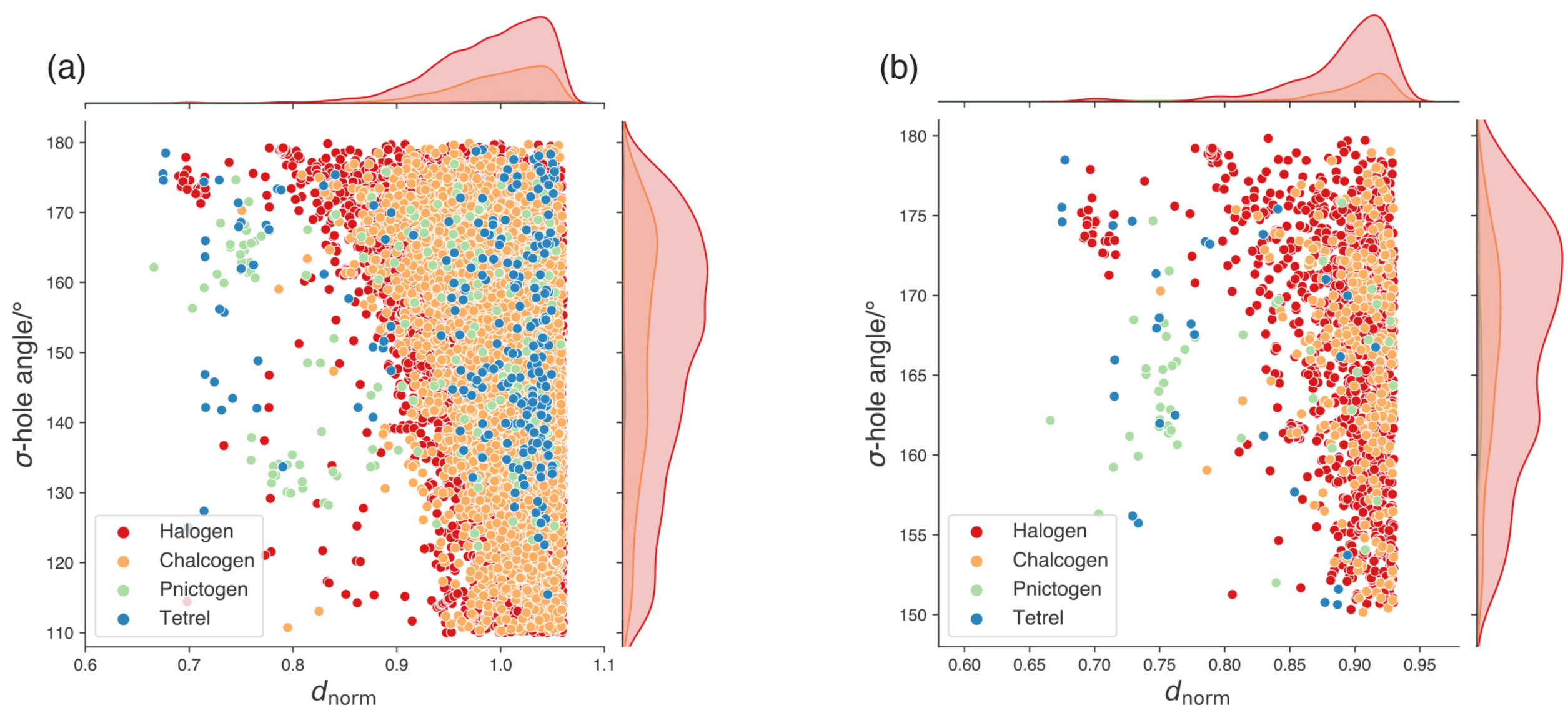

FIG. S4: Normalised distance ($d_{\rm norm}$) and σ-hole angle from searches of the CSD for σ-hole interactions with divalent oxygen as the acceptor atom on the Lewis base. (a) Data for the full search criteria (see text), which resulted in 14,264 "hits". (b) Data for the 1,541 results with $d_{\rm norm} \leqslant 0.93$ and σ-hole angle $\geqslant 150°$. Also shown are kernel density estimate plots of the distribution of the normalised distances and σ-hole angles grouped by interaction class.

S5.3. Comparison of σ-hole angles in SHEF1552 and the subset of CSD search results

A comparison of the σ-hole angles in the SHEF1552 dataset and the subset of CSD search results ($d_{\text{norm}} \leqslant 0.93$ and σ-hole angle $\geqslant 150°$) shows strong qualitative agreement (Figure S5).

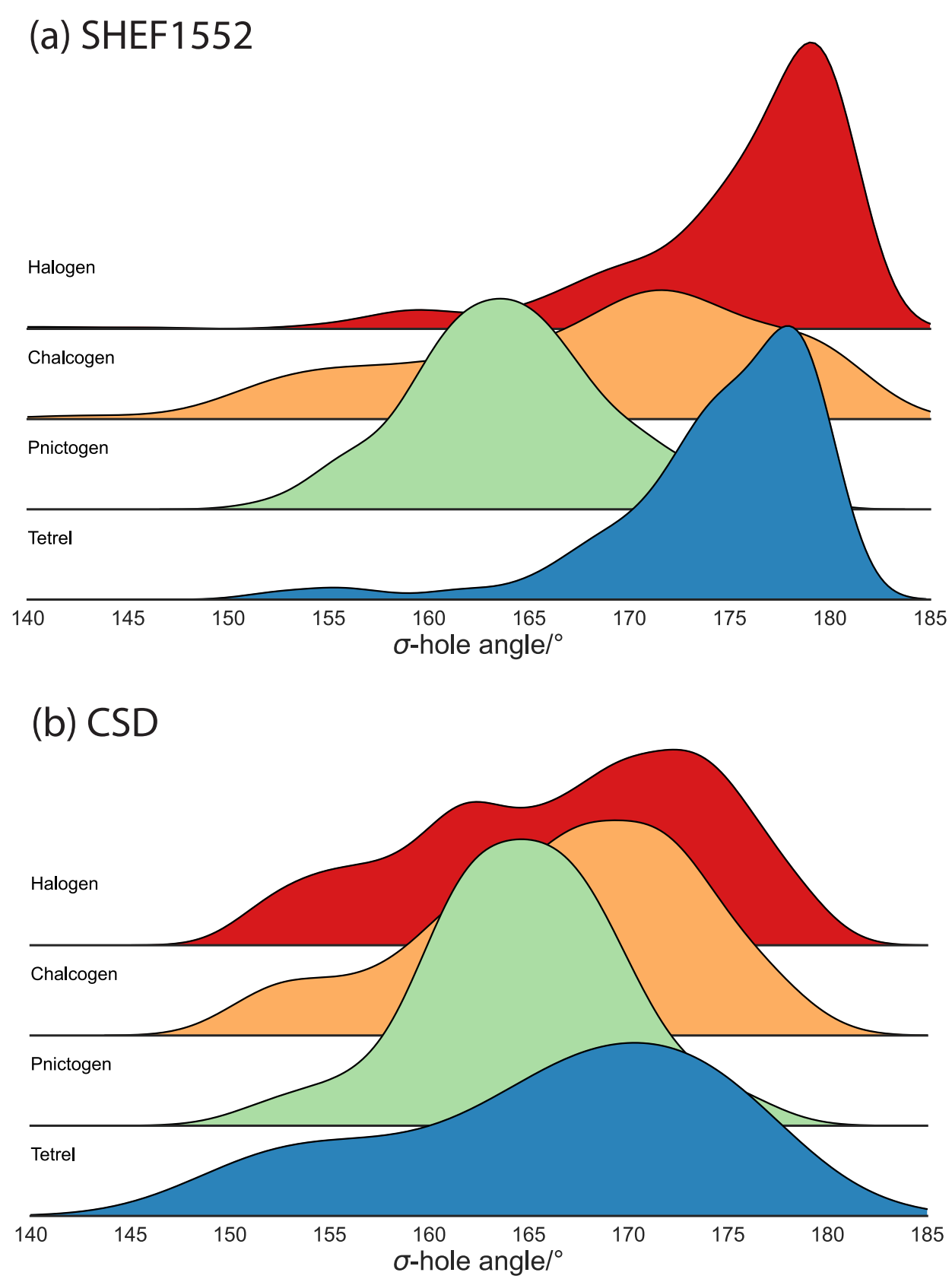

FIG. S5: Kernel density estimate plots, per interaction class, of the distribution of σ-hole angles found in: (a) the SHEF1552 dataset of computational gas-phase structures, (b) a subset of the CSD search results for σ-hole interactions with divalent oxygen as the acceptor atom on the Lewis base. The visualisations of the distributions contain smoothing "tails", hence they appear to extend slightly beyond the maximum and minimum values contained in the datasets.

S6. INTERACTION ENERGIES

The designated benchmark for interaction energies in SHEF1552 is CP-corrected LCCSD(T*)-F12. The relationship between this quantitative measure of the interaction strength and the geometric parameters is explored in Section 3.2 of the main text. Figure S6 shows the variation of the interaction energy with σ-hole angle, similar to Figure 6(b), but does not include any of the pnictogen-bond interactions.

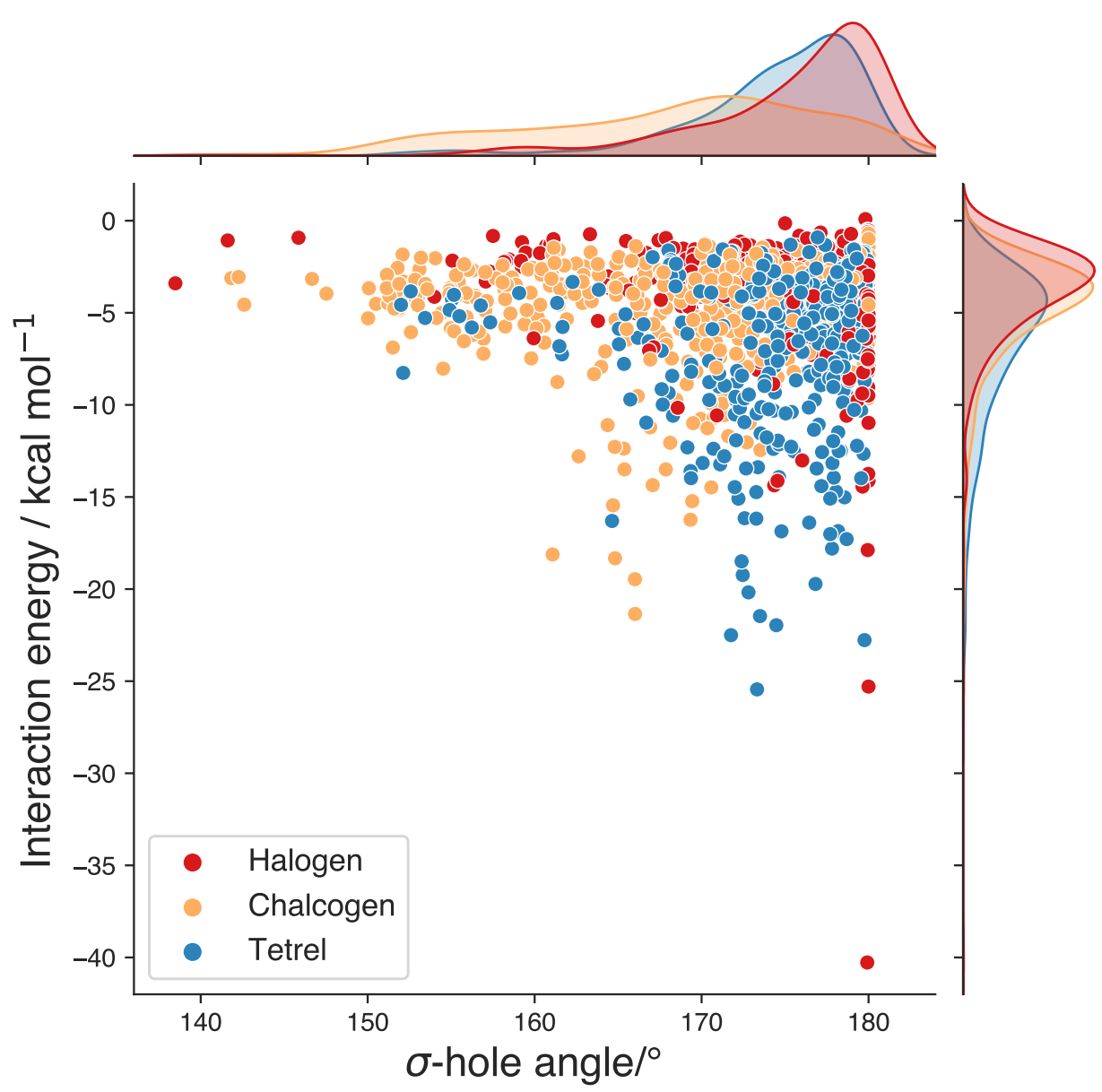

FIG. S6: Counterpoise-corrected LCCSD(T*)-F12 interaction energies plotted against the σ-hole angle, specifically excluding any pnictogen-bond interactions. All geometries were optimised at the M06-2X-D3(0)/def2-TZVPD level of theory. Also shown are kernel density estimate plots of the distributions grouped by interaction class.

S6.1. Interaction energies subdivided into interaction classes

Figure S7 plots interaction energy against d_{norm}, subdivided by interaction class. Summary statistics for these data are presented in Table S11, and further broken down by σ-hole donor atom in Table S12.

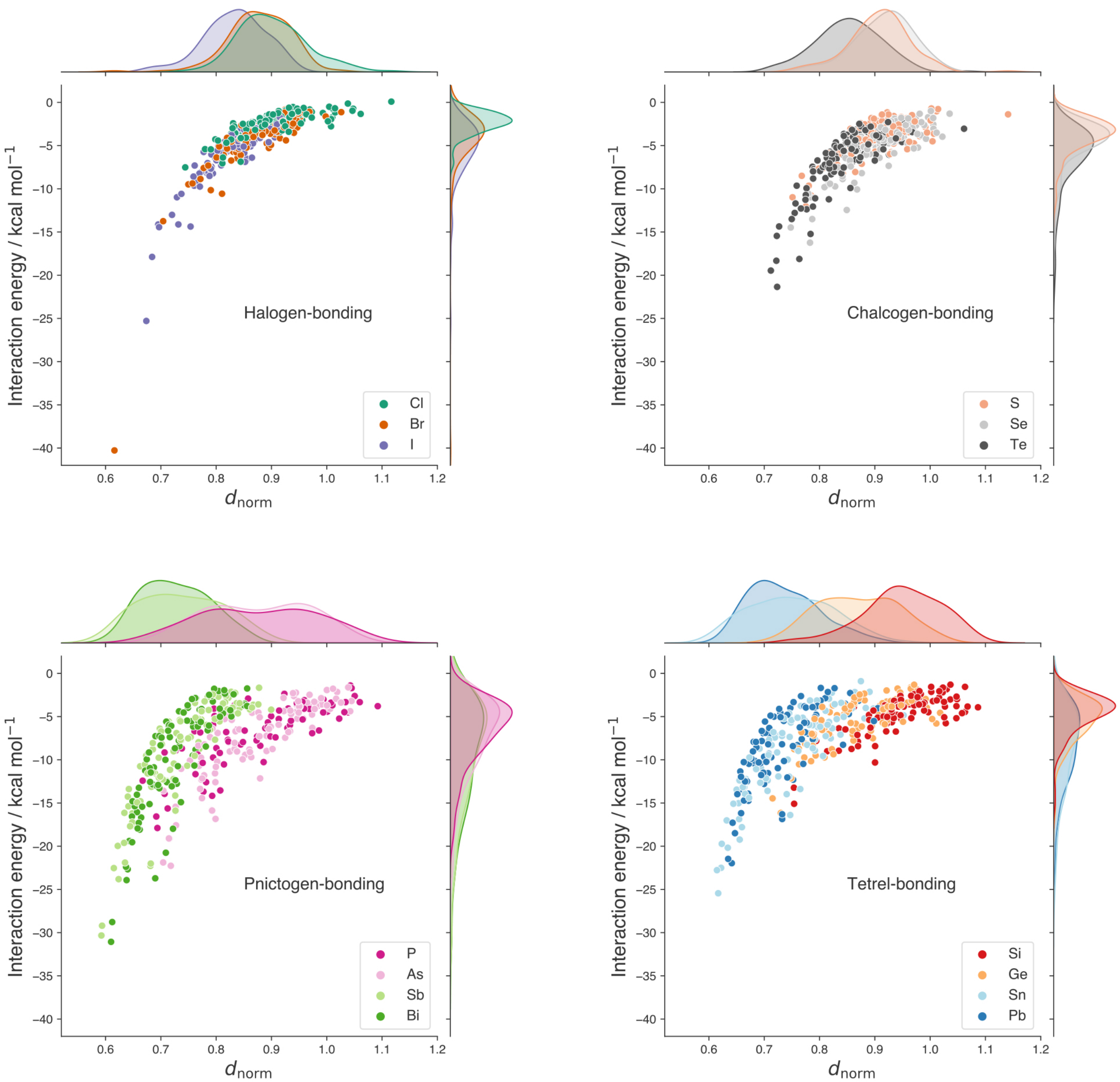

FIG. S7: Normalised distance (d_{norm}) and LCCSD(T*)-F12b interaction energies (kcal mol^{-1}) subdivided into halogen, chalcogen, pnictogen and tetrel interaction classes. Each of these interaction classes are also shown as kernel density estimate plots grouped by σ-hole donor atom.

TABLE S11: Summary statistics of the counterpoise-corrected LCCSD(T*)-F12b interaction energies (kcal mol^{-1}) grouped by interaction class.

Class	Mean	S.D.	Min	Max	Q1	Q3
Halogen	−3.96	3.30	−40.27	+0.09	−4.97	−2.11
Chalcogen	−4.94	3.07	−21.35	−0.70	−5.88	−2.96
Pnictogen	−8.38	5.54	−31.06	−1.40	−11.23	−4.13
Tetrel	−6.84	4.24	−25.45	−0.90	−8.73	−3.82

TABLE S12: Summary statistics of the counterpoise-corrected LCCSD(T*)-F12b interaction energies (kcal mol^{-1}) grouped by interaction class and σ-hole donor atom.

Class	Donor atom	Mean	S.D.	Min	Max	Q1	Q3
Halogen	Cl	−2.39	1.35	−7.52	+0.09	−3.05	−1.39
	Br	−4.04	3.81	−40.27	−0.73	−4.66	−2.35
	I	−5.45	3.43	−25.29	−1.19	−6.53	−3.23
Chalcogen	S	−3.80	2.09	−12.03	−0.70	−4.55	−2.40
	Se	−4.68	2.67	−16.24	−1.00	−5.33	−2.90
	Te	−6.31	3.69	−21.35	−1.84	−7.48	−3.83
Pnictogen	P	−6.75	3.73	−19.12	−1.40	−8.65	−4.06
	As	−7.49	4.56	−22.26	−1.62	−9.85	−3.92
	Sb	−9.46	6.31	−30.33	−1.66	−12.80	−4.28
	Bi	−9.80	6.47	−31.06	−1.75	−13.27	−4.46
Tetrel	Si	−4.64	2.36	−15.09	−1.31	−5.45	−3.18
	Ge	−5.46	2.70	−16.15	−1.32	−6.90	−3.63
	Sn	−8.95	5.17	−25.45	−0.90	−12.33	−5.14
	Pb	−8.33	4.40	−21.96	−1.68	−10.78	−4.85

The scatterplot of interaction energy against d_{norm} [Figure 6(a)] shows a boomerang-like shape that can be thought of as two regions with different gradients of this relationship. The stronger interactions have a steeper gradient, such that the energy changes rapidly with (normalised) distance, whereas the interactions with $d_{\text{norm}} > 0.8$ have a shallower gradient.

This relationship can also be seen in Figure S8, where an exponential has been fit to the data that has an RMSE of 2.54 kcal mol^{-1}.

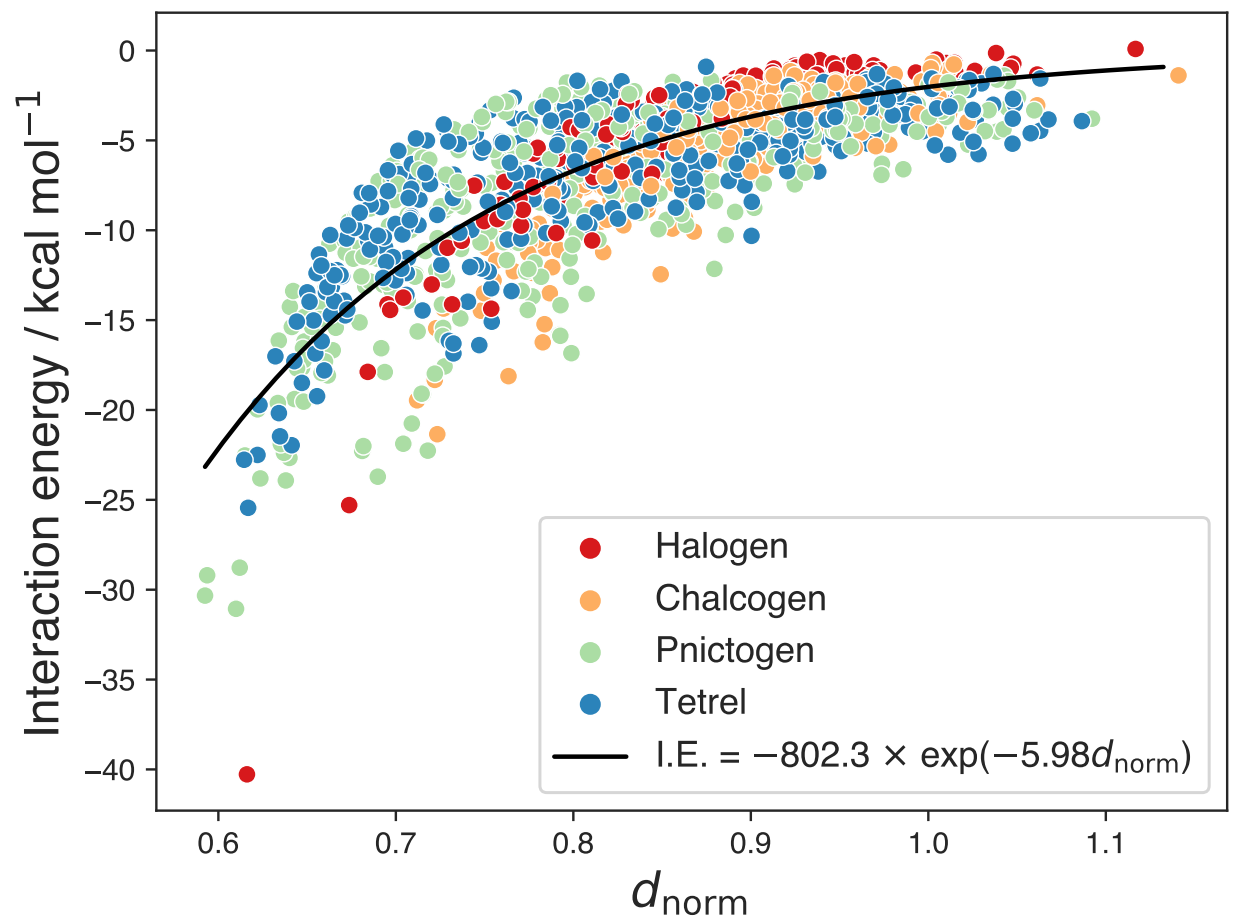

FIG. S8: Counterpoise-corrected LCCSD(T*)-F12 interaction energies plotted against the normalised distance (d_{norm}). An exponential function fit to the data is also shown.

S6.2. Interpenetration of van der Waals crusts

To further assess the contact distance between N-bromosuccinimide and trimethylphosphine, the degree of interpenetration of the van der Waals crusts of the Br and P have been calculated using the index (p_{AB}) of Alvarez and co-workers:[47,48]

$$p_{\mathrm{AB}} = \frac{v_{\mathrm{A}} + v_{\mathrm{B}} - d_{\mathrm{AB}}}{v_{\mathrm{A}} + v_{\mathrm{B}} - r_{\mathrm{A}} - r_{\mathrm{B}}} \times 100\%$$

where v_{A} and v_{B} are the van der Waals radii of atoms A and B, respectively, r_{A} and r_{B} are the covalent radii of the atoms, and d_{AB} is the interatomic distance between A and B. A p_{AB} value of 0% corresponds to the distance between the two atoms being equal to the sum of their van der Waals radii (or $d_{\mathrm{norm}} = 1.0$), whereas 100% corresponds to this distance being the sum of their covalent radii. The "crusts" terminology of Alvarez and co-workers thus refers to the section of the van der Waals sphere between the covalent and van der Waals radii. The value of p_{AB} for the N-bromosuccinimide$\cdots$trimethylphosphine complex is 96.88%, indicating this is very close to a single covalent bond and rationalising the strength of the interaction.

A scatter plot of the interaction energy against p_{AB} for all complexes in the SHEF1552 dataset is presented in Figure S9(a), where the overall distribution once again has a boomerang-like shape, although more strongly interacting complexes now appear to the lower right of the plot. Figure S9(b) shows the same points with a fifth order polynomial fit to the data, which has an RMSE of 2.25 kcal mol^{-1}. Although more sophisticated models for predicting the strength of halogen bonding interactions already exist,[49–52] it is interesting that this simple curve fitting to a single distance-based descriptor produces a reasonable RMSE. Indeed, using p_{AB} leads to a lower RMSE than d_{norm} (Figure S7), suggesting that p_{AB} may be a better descriptor for the strength/distance relationship than using the normalised distance alone.

Statistical details on p_{AB} grouped by interaction class are presented in Table S13, which can be compared with the d_{norm} data summarised in Table S8. As one might expect, the class with the shortest average d_{norm} (pnictogen bonds) also has the greatest p_{AB} index. Other trends noted in the main text (Section 3.1) for d_{norm} also map onto p_{AB}. The summary statistics for p_{AB} are further subdivided by σ-hole donor atom in Table S14.

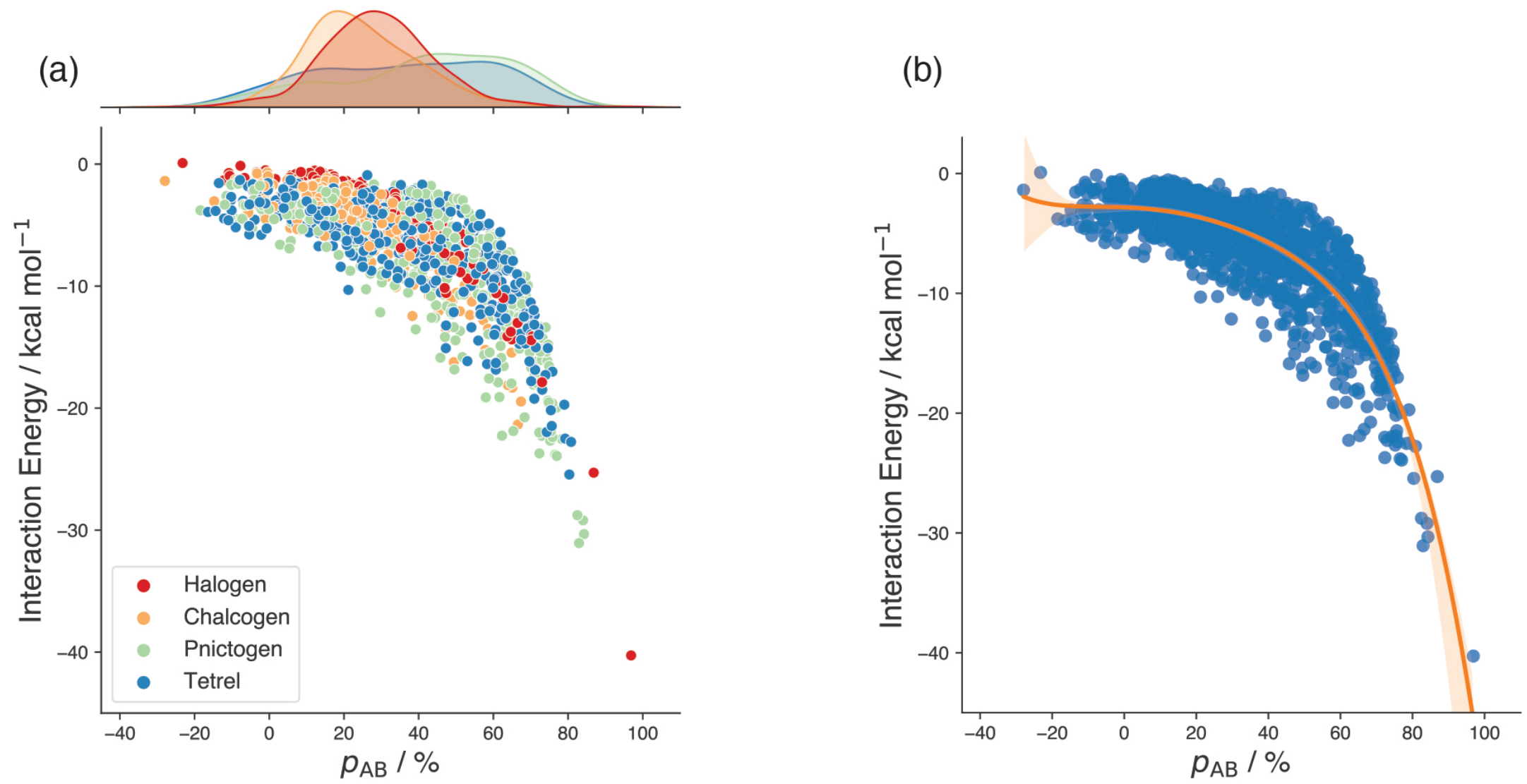

FIG. S9: Counterpoise-corrected LCCSD(T*)-F12 interaction energies plotted against the interpenetration of van der Waals crusts (p_{AB})[47,48]. (a) indicates the class of interaction and includes kernel density estimate plots of the p_{AB} distributions grouped by interaction class. (b) a fifth order polynomial fit to the data (solid orange line) with the 95% confidence interval shown as translucent bands.

TABLE S13: Summary statistics of the interpenetration of van der Waals crusts (p_{AB}, as %) from M06-2X-D3(0)/def2-TZVPD optimised geometries grouped by interaction class

	Full dataset	Tetrel	Pnictogen	Chalcogen	Halogen
Mean	32.7	35.9	41.7	24.6	29.2
S.D.	20.6	23.6	23.4	14.8	15.1
Min	−28.0	−16.4	−18.4	−28.0	−23.2
Max	96.9	80.8	84.3	67.4	96.9
Q1	17.1	15.7	26.0	14.7	19.7
Q3	47.0	56.0	61.3	33.9	37.9

TABLE S14: Summary statistics of the interpenetration of van der Waals crusts (p_{AB}, as %) grouped by interaction class and σ-hole donor atom. Geometries were optimised at the M06-2X-D3(0)/def2-TZVPD level

Class	Donor atom	Mean	S.D.	Min	Max	Q1	Q3
Halogen	Cl	19.8	12.5	−23.2	50.9	12.5	27.4
	Br	27.7	12.8	−6.6	96.9	18.8	34.2
	I	39.9	12.6	15.2	86.9	31.2	47.0
Chalcogen	S	18.7	11.3	−28.0	49.4	12.7	24.7
	Se	19.1	12.1	−9.0	55.9	11.4	26.6
	Te	35.7	13.8	−14.8	67.4	26.4	43.9
Pnictogen	P	25.1	21.3	−18.4	66.6	8.7	40.8
	As	27.3	20.5	−11.0	65.4	10.9	44.1
	Sb	55.2	14.9	22.8	84.3	43.5	67.2
	Bi	58.7	12.1	33.8	82.9	49.7	68.0
Tetrel	Si	9.4	13.5	−16.4	47.3	−0.4	16.3
	Ge	25.7	13.7	−3.9	56.0	15.4	36.8
	Sn	53.0	14.9	9.9	80.8	42.1	63.4
	Pb	55.5	12.1	19.3	75.6	48.1	63.8

S6.3. Interaction energies from other levels of theory: Errors relative to benchmark data

The CP-LCCSD(T*)-F12 method is used to compute the benchmark interaction energies for the SHEF1552 dataset, but CP-LMP2-F12, SAPT0, SAPT2, and CP-M06-2X-D3(0) interaction energies were also computed as part of this work (full technical detail in Section S3). The error for each of these methods (ΔI.E.), relative to the benchmark, has been calculated as the CP-LCCSD(T*)-F12 value minus the comparison method value, such that a positive value corresponds to the method predicting the interaction is stronger than the benchmark value. Figure S10 presents the errors for each method as violin plots for the full SHEF1552 dataset. Figures S11–S14 show similar violin plots for each individual interaction class.

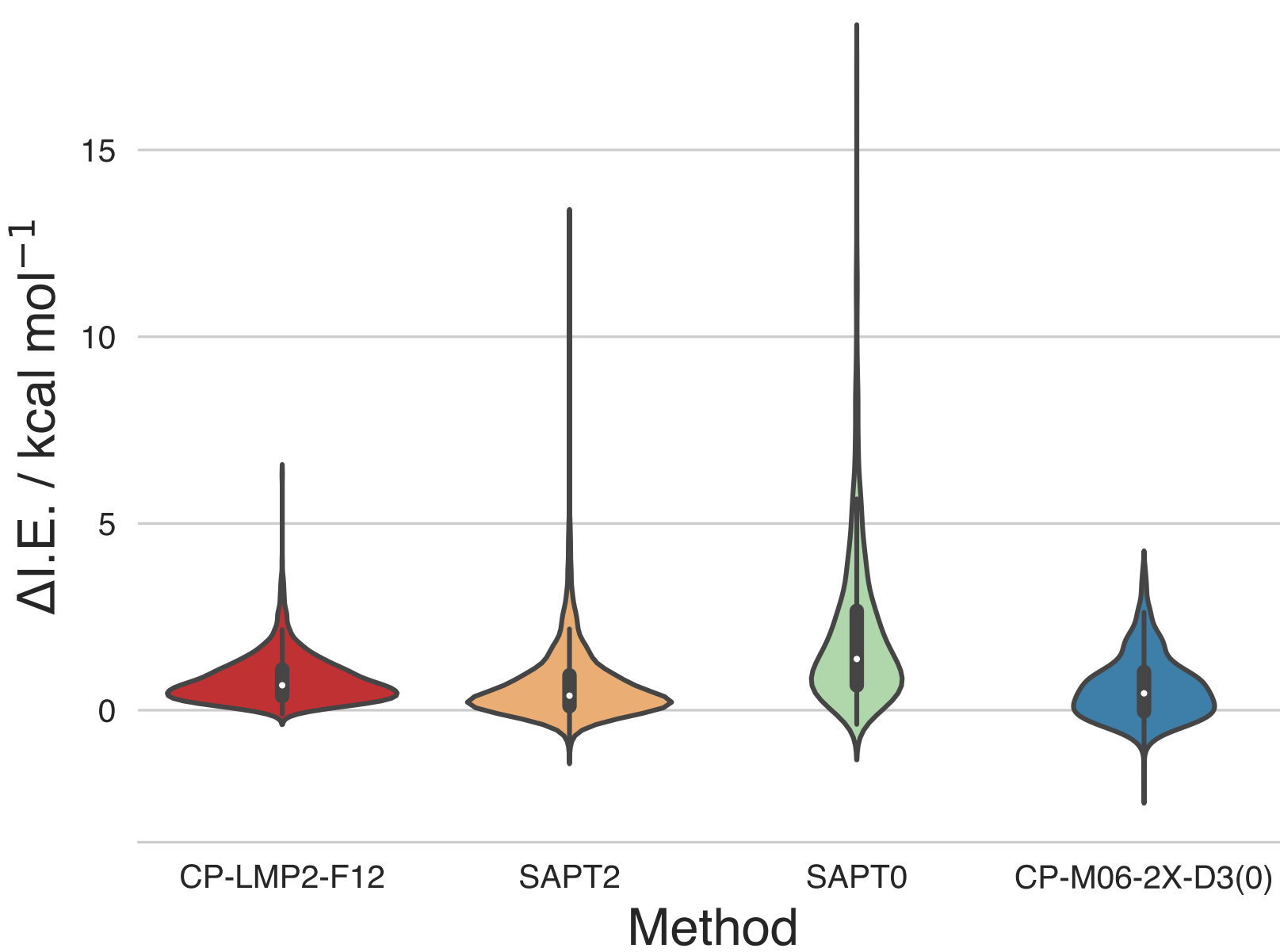

FIG. S10: Violin plots of the error in interaction energies, ΔI.E., (kcal mol^{-1}) relative to the CP-LCCSD(T*)-F12/aug-cc-pVDZ-F12 benchmark data. All geometries were optimised at the M06-2X-D3(0)/def2-TZVPD level of theory.

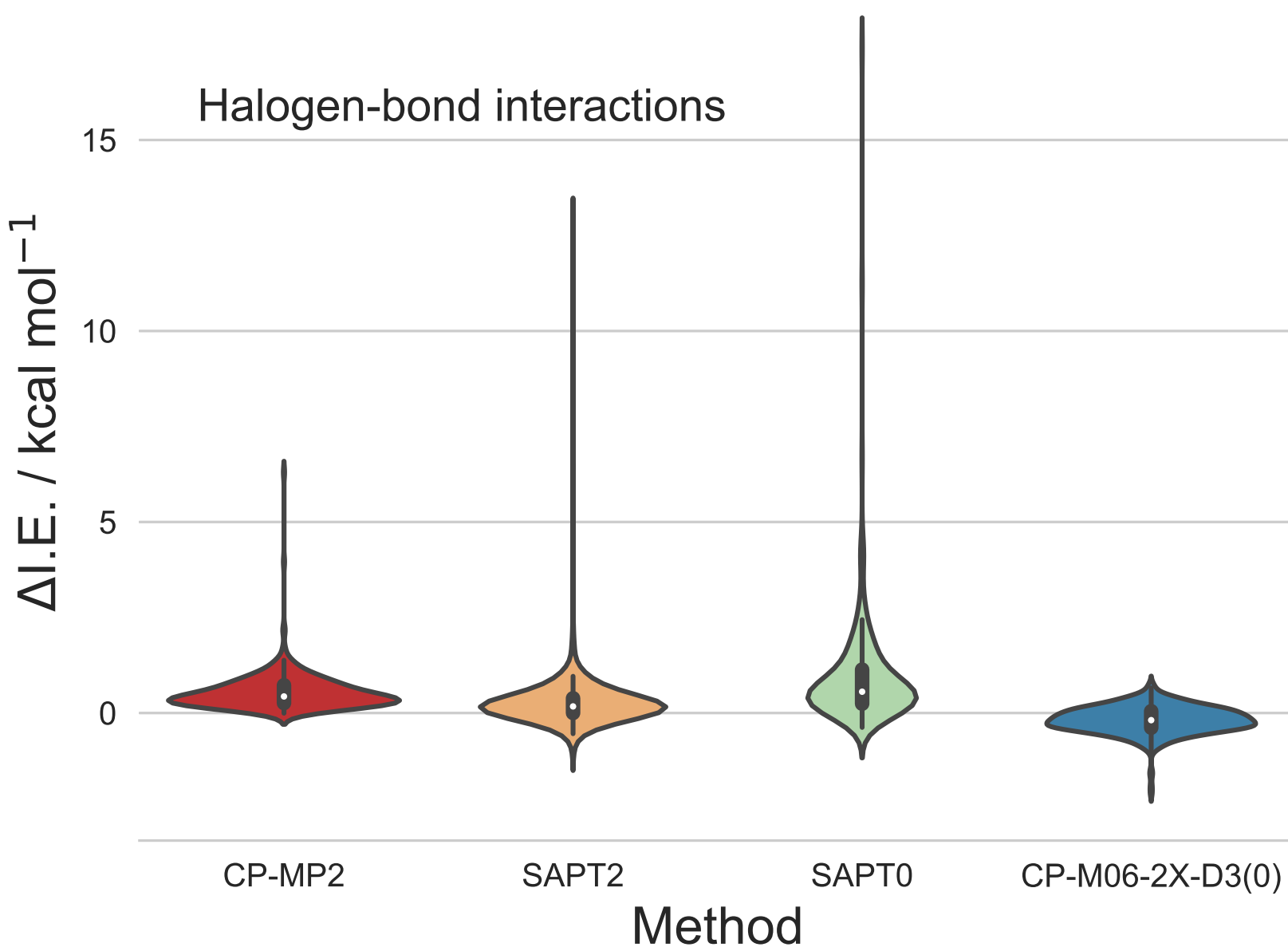

FIG. S11: Violin plots of the error in halogen bond interaction energies, ΔI.E. (kcal mol^{-1}), calculated as the CP-LCCSD(T*)-F12/aug-cc-pVDZ-F12 benchmark energy minus that of the method tested. All geometries were optimised at the M06-2X-D3(0)/def2-TZVPD level of theory.

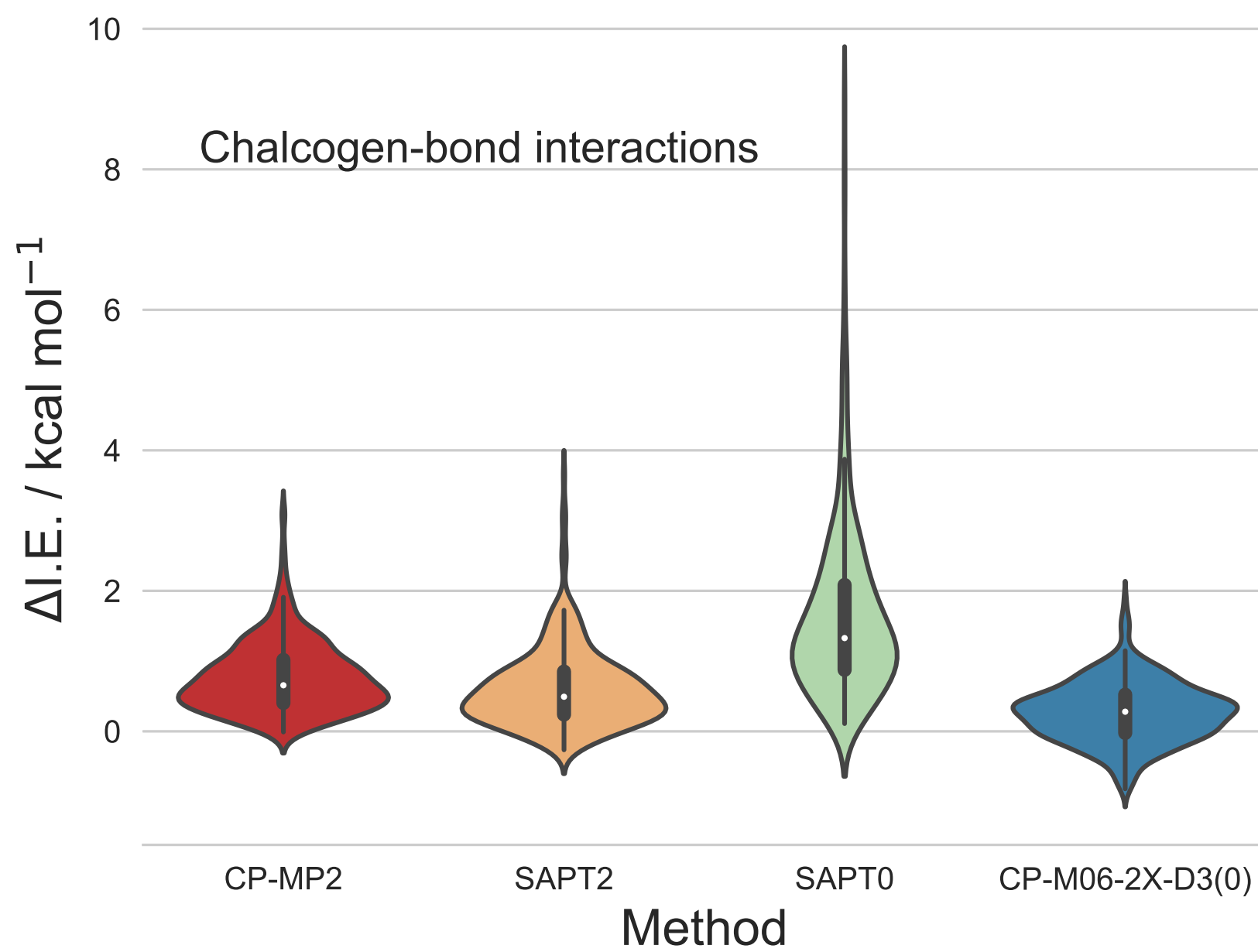

FIG. S12: Violin plots of the error in chalcogen bond interaction energies, ΔI.E. (kcal mol^{-1}), calculated as the CP-LCCSD(T*)-F12/aug-cc-pVDZ-F12 benchmark energy minus that of the method tested. All geometries were optimised at the M06-2X-D3(0)/def2-TZVPD level of theory.

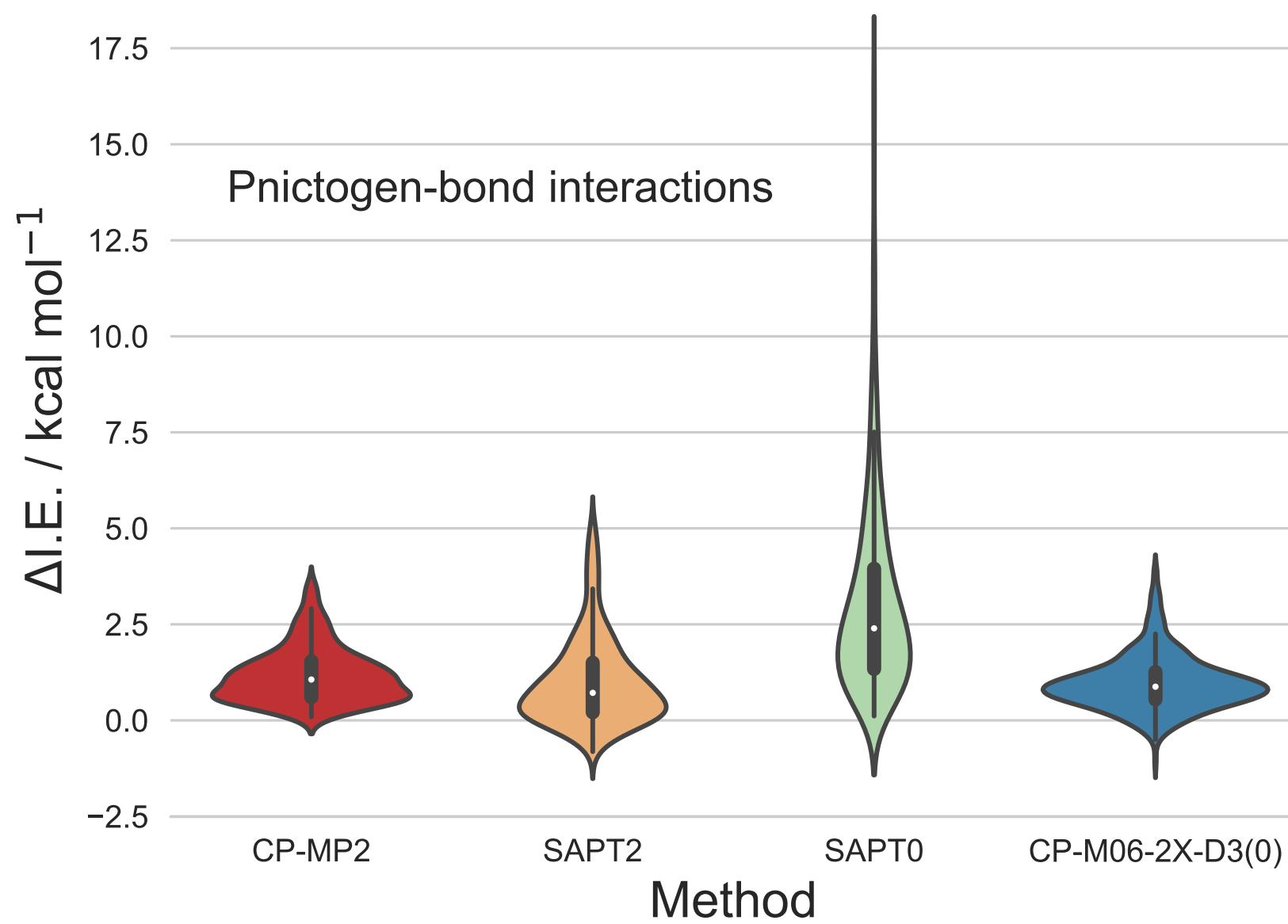

FIG. S13: Violin plots of the error in pnictogen bond interaction energies, ΔI.E. (kcal mol^{-1}), calculated as the CP-LCCSD(T*)-F12/aug-cc-pVDZ-F12 benchmark energy minus that of the method tested. All geometries were optimised at the M06-2X-D3(0)/def2-TZVPD level of theory.

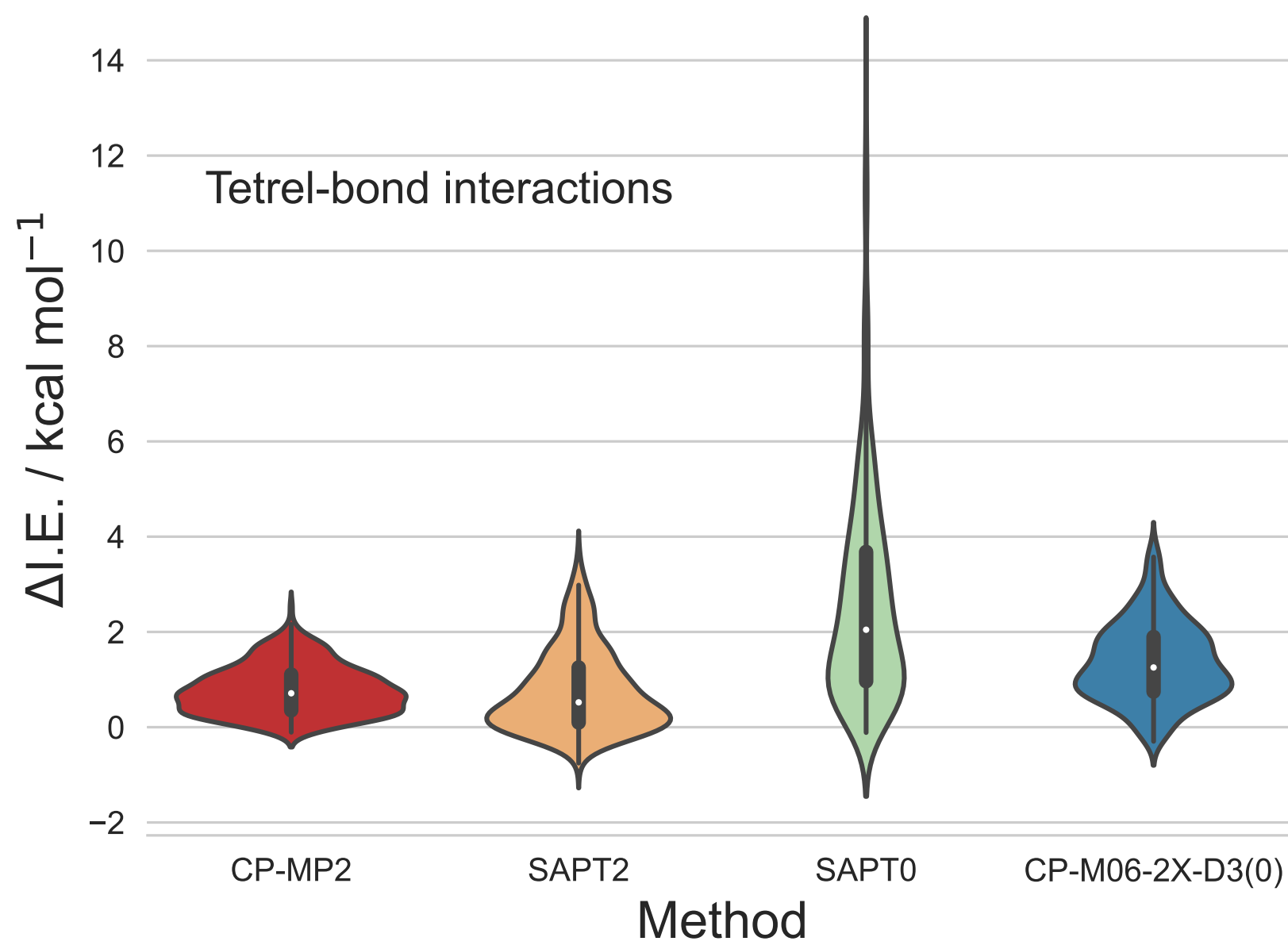

FIG. S14: Violin plots of the error in tetrel bond interaction energies, ΔI.E. (kcal mol^{-1}), calculated as the CP-LCCSD(T*)-F12/aug-cc-pVDZ-F12 benchmark energy minus that of the method tested. All geometries were optimised at the M06-2X-D3(0)/def2-TZVPD level of theory.

S6.4. SAPT Analysis

SAPT analysis reveals how the interaction energy is constructed from electrostatic, exchange, induction and dispersion terms. Herein, this analysis was conducted using the SAPT2 truncation, with full technical details in Section S3. We also use the relative importance of these terms to classify each intermolecular complex as either electrostatic-dominated, dispersion-dominated or mixed-character, with the criteria for doing so defined in Section 3.3 of the main text. Figure S15 shows histograms of the distributions of the electrostatic, induction and dispersion components for the entire SHEF1552 dataset, expressed as a percentage of the total SAPT attractive components.

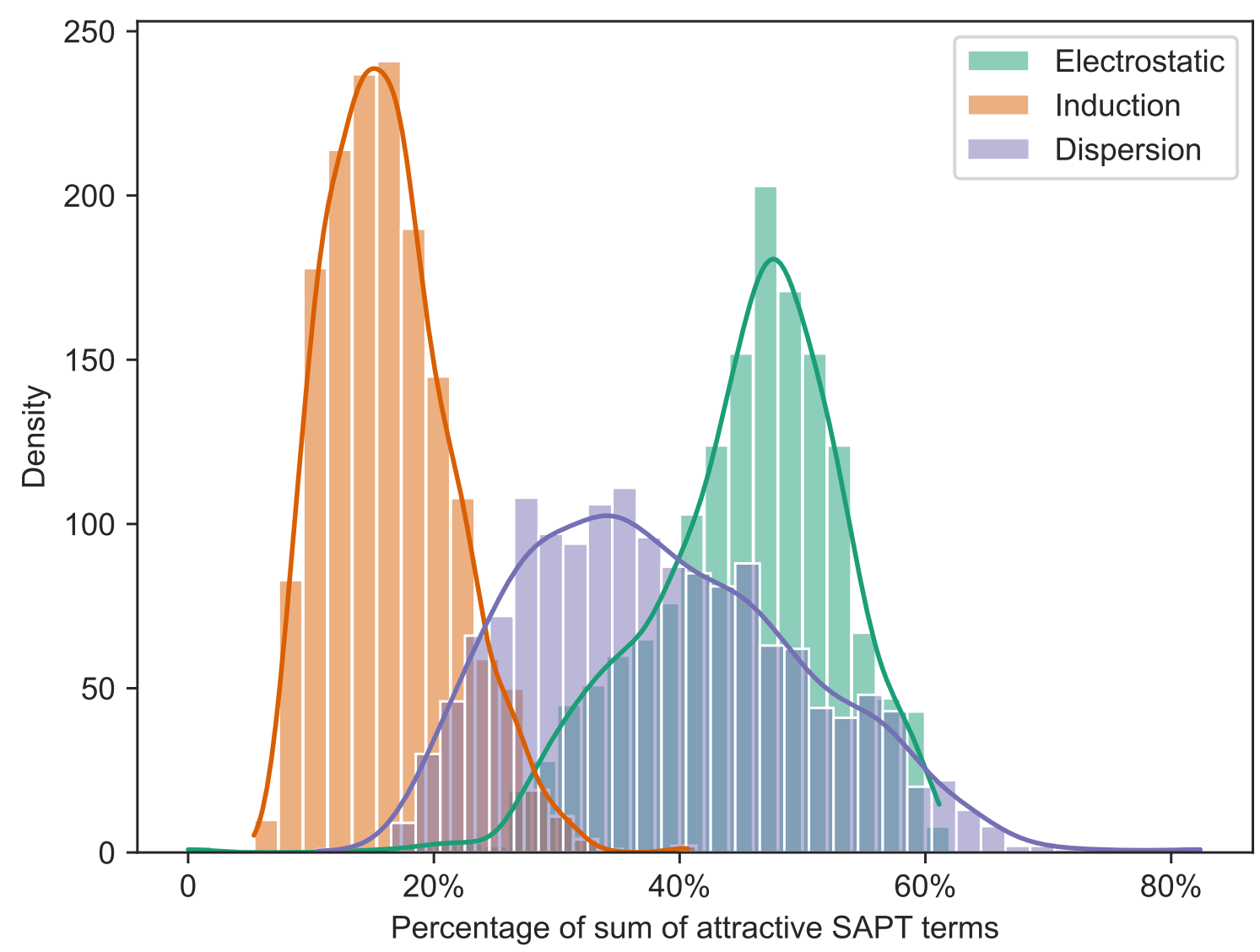

FIG. S15: Histograms of the SAPT electrostatic, induction and dispersion components of the interaction energy, expressed as a percentage of the total SAPT attractive components.

Figures S16–S18 are scatterplots of the benchmark interaction energy against d_{norm}, separated into the electrostatic-dominated, mixed-character and dispersion-dominated classifications.

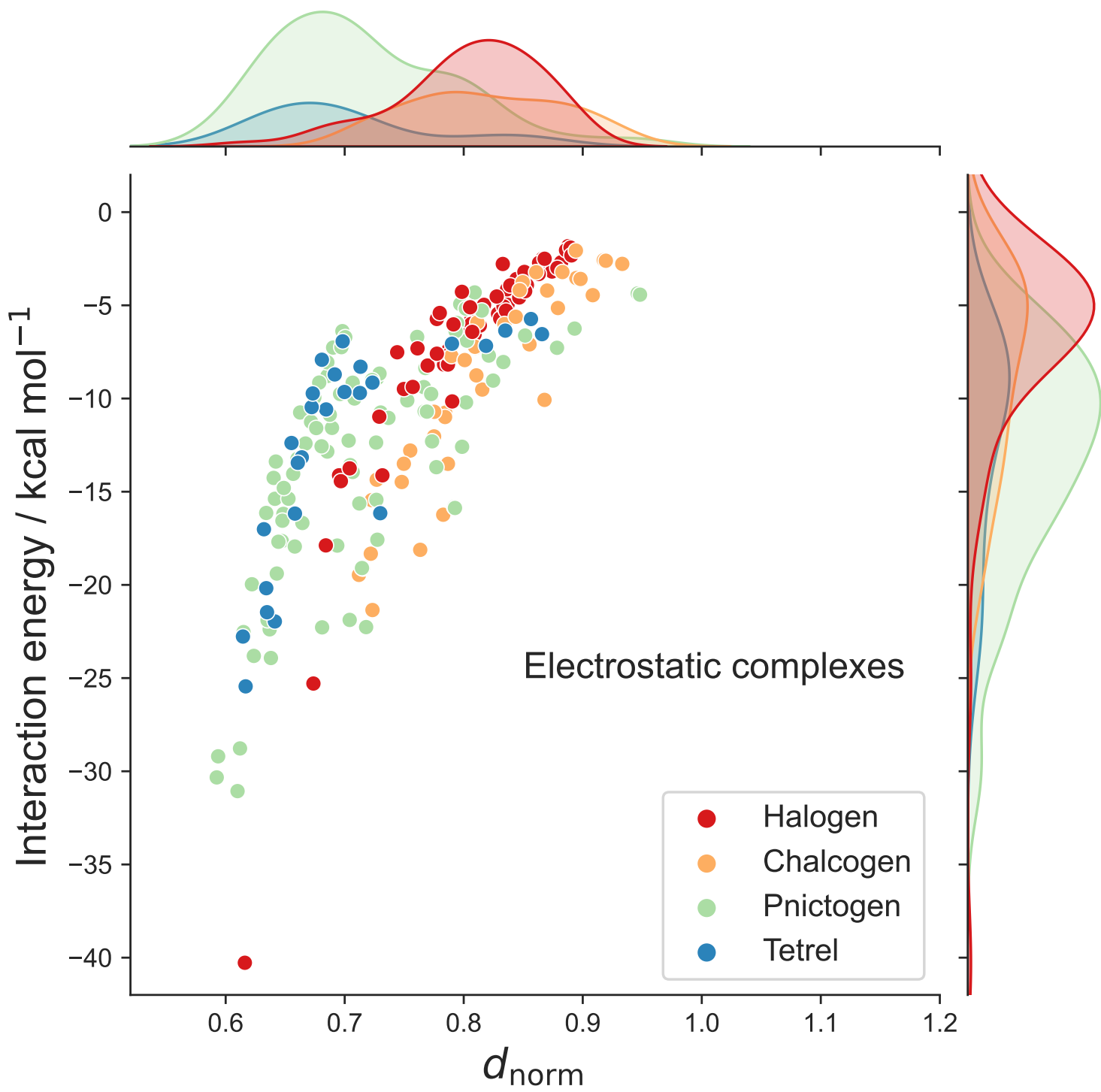

FIG. S16: Normalised distance (d_{norm}) and CP-LCCSD(T*)-F12b interaction energies (kcal mol^{-1}) of the electrostatic-dominated complexes, as determined by SAPT analysis, subdivided into σ-hole interaction class. Each of these interaction classes are also shown as kernel density estimate plots.

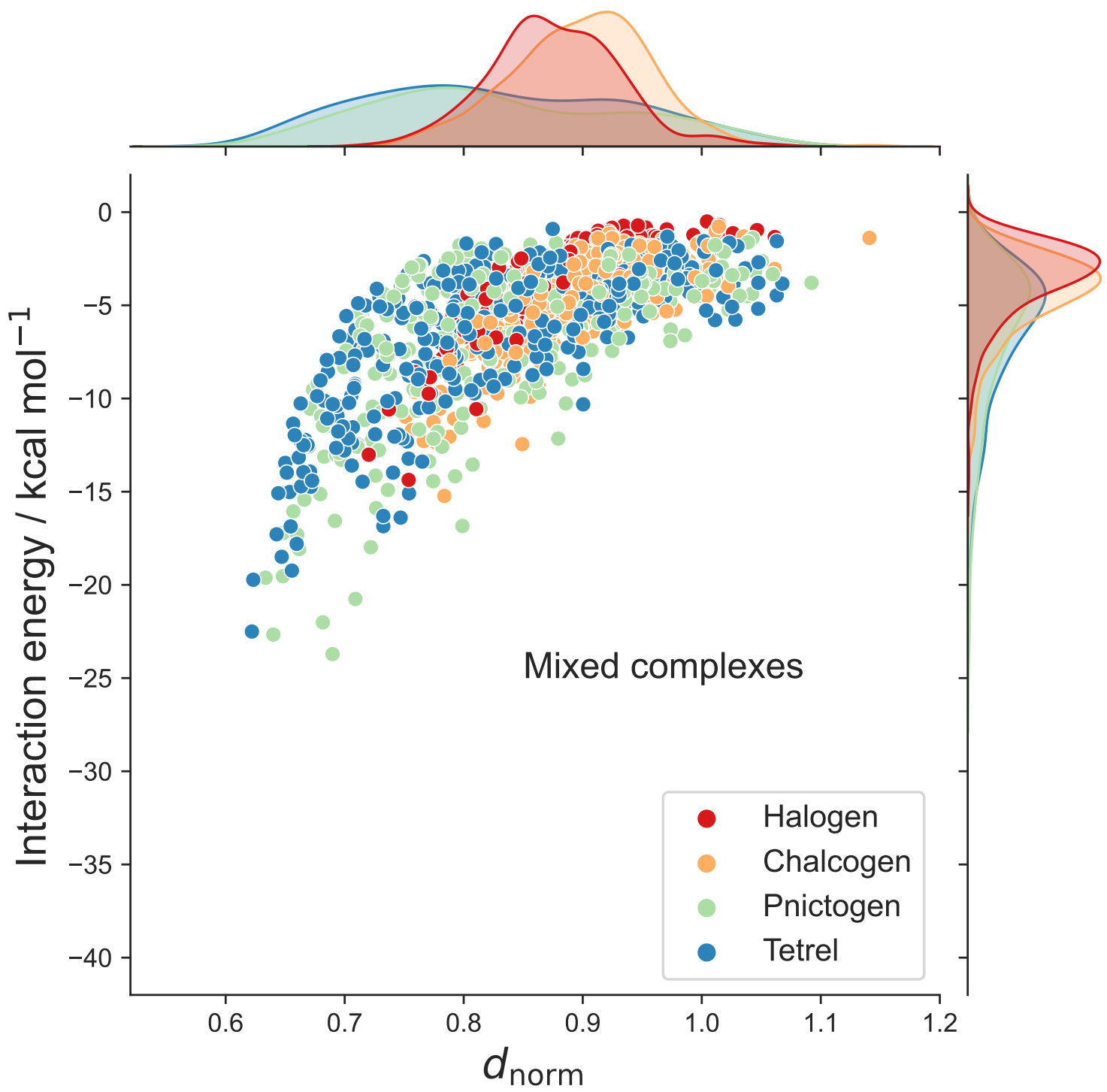

FIG. S17: Normalised distance (d_{norm}) and CP-LCCSD(T*)-F12b interaction energies (kcal mol^{-1}) of the mixed-character complexes, as determined by SAPT analysis, subdivided into σ-hole interaction class. Each of these interaction classes are also shown as kernel density estimate plots.

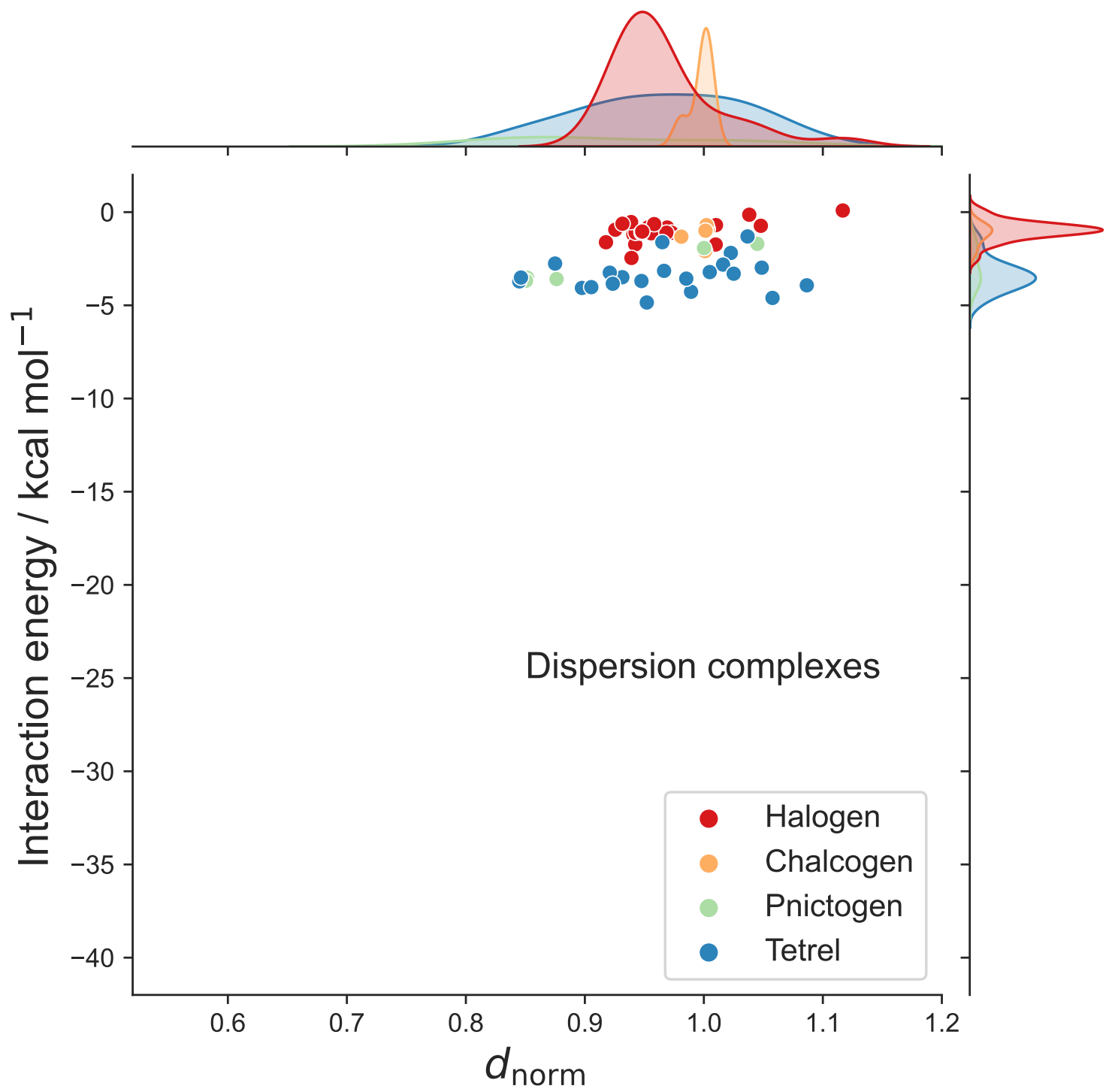

FIG. S18: Normalised distance (d_{norm}) and CP-LCCSD(T*)-F12b interaction energies (kcal mol^{-1}) of the dispersion-dominated complexes, as determined by SAPT analysis, subdivided into σ-hole interaction class. Each of these interaction classes are also shown as kernel density estimate plots.

Figures S19–S21 show scatterplots of the benchmark interaction energy against the SAPT induction component, with the latter expressed as a percentage of the sum of the attractive SAPT terms.

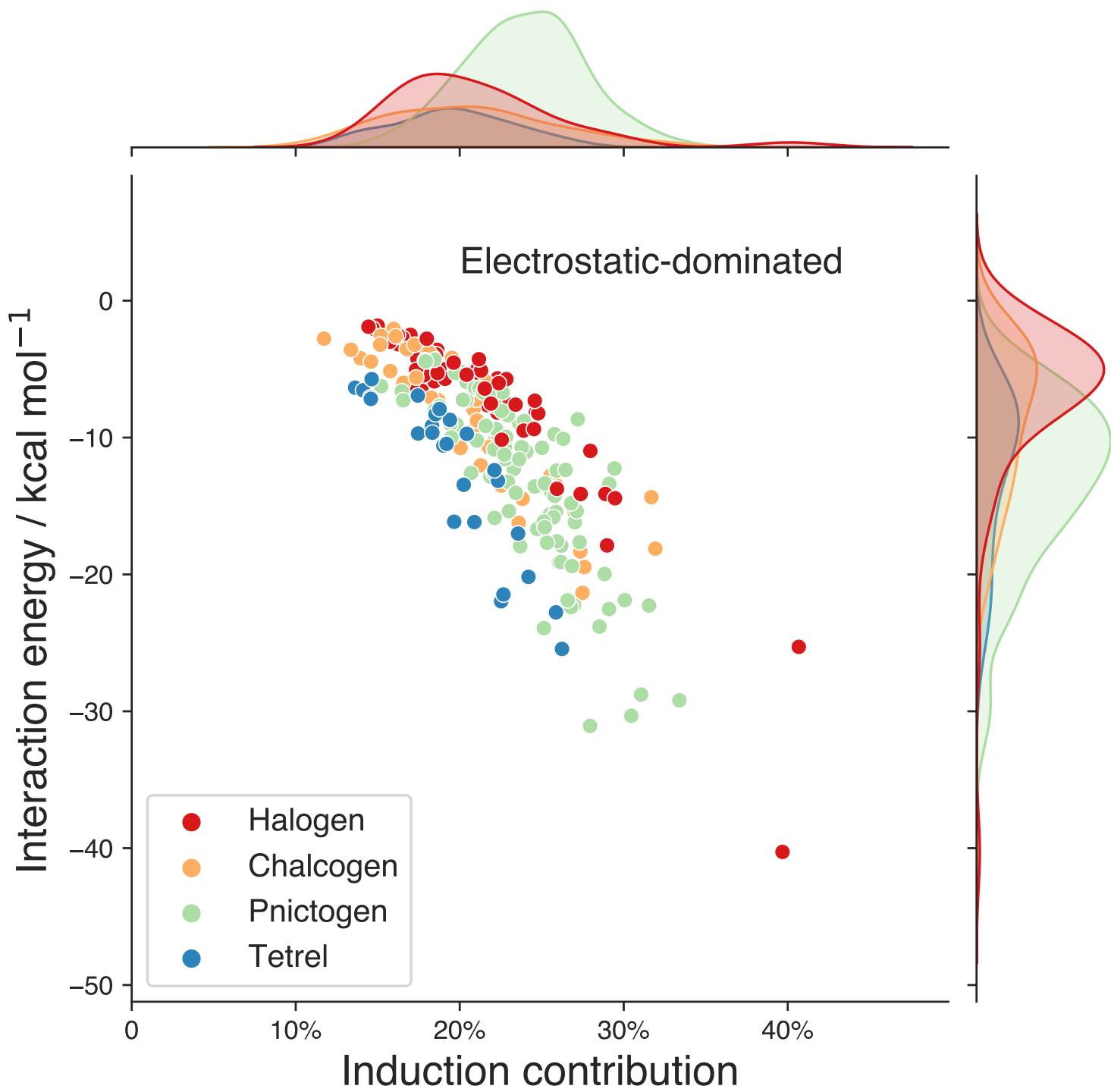

FIG. S19: Counterpoise corrected LCCSD(T*)-F12 interaction energies, for complexes classified as electrostatic-dominated, plotted against the percentage contribution of the induction component to the total of the attractive SAPT terms. Also shown are kernel density estimate plots of the induction contributions grouped by interaction class.

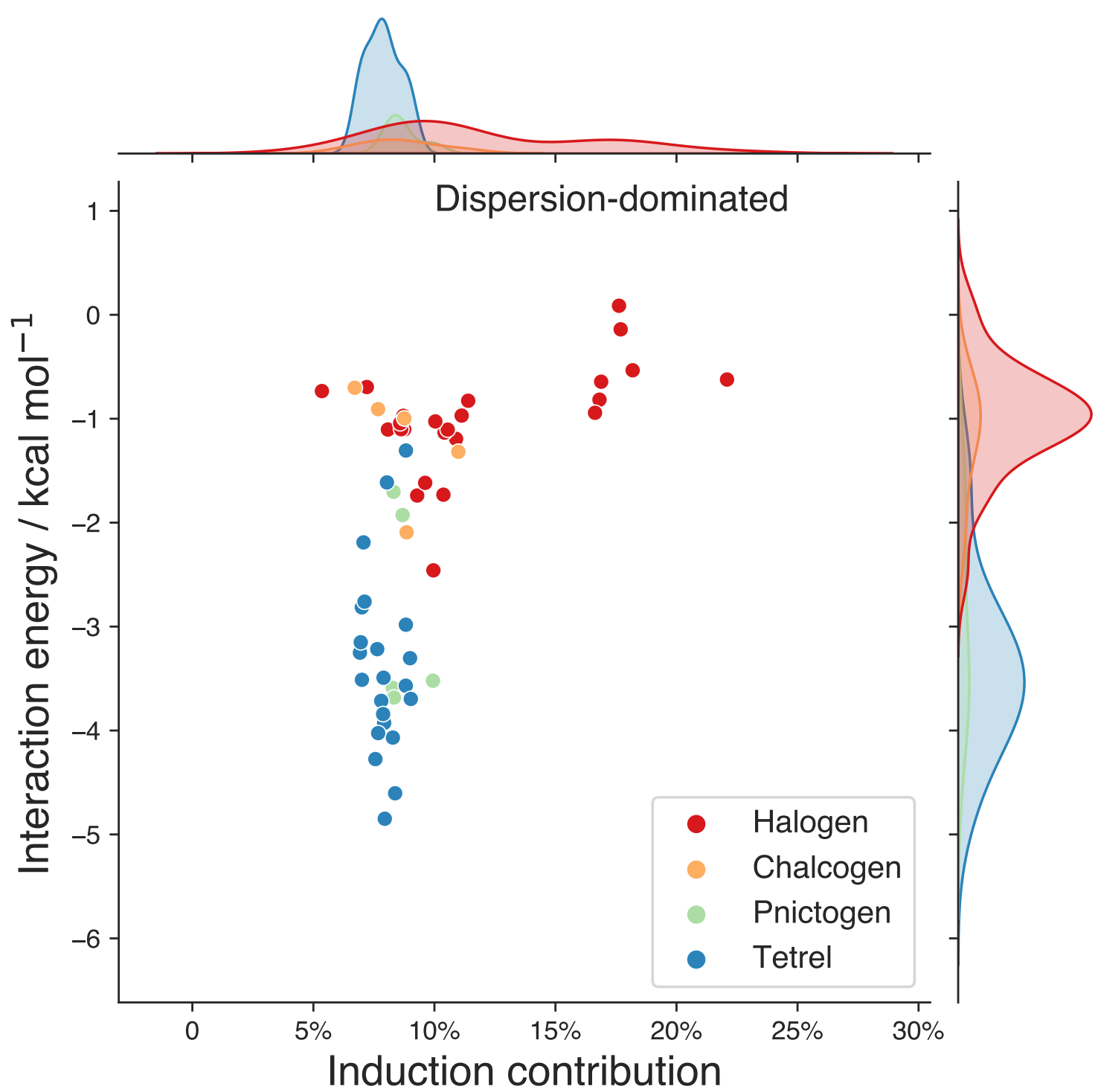

FIG. S20: Counterpoise corrected LCCSD(T*)-F12 interaction energies, for complexes classified as dispersion-dominated, plotted against the percentage contribution of the induction component to the total of the attractive SAPT terms. Also shown are kernel density estimate plots of the induction contributions grouped by interaction class.

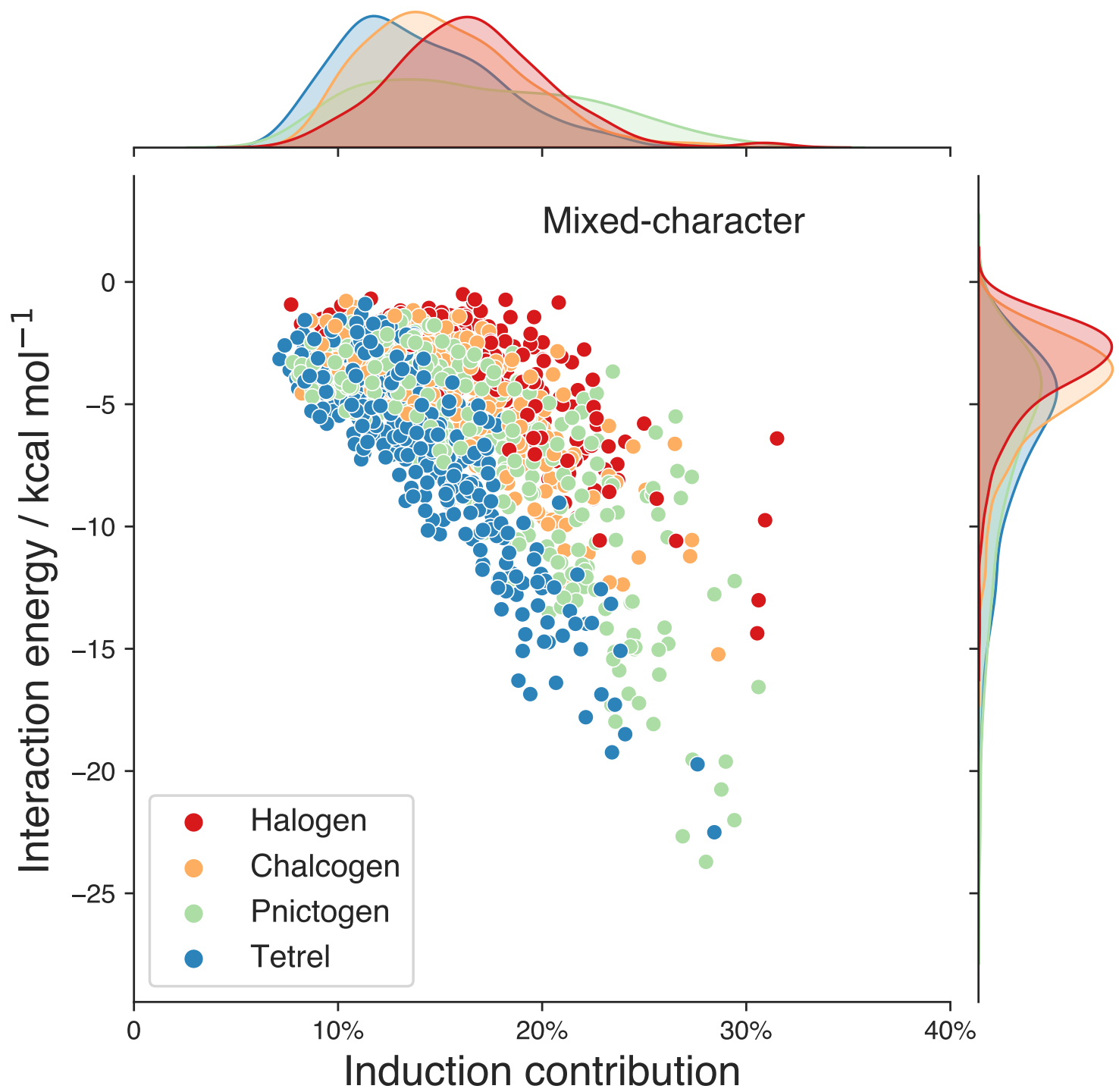

FIG. S21: Counterpoise corrected LCCSD(T*)-F12 interaction energies, for complexes classified as mixed-character, plotted against the percentage contribution of the induction component to the total of the attractive SAPT terms. Also shown are kernel density estimate plots of the induction contributions grouped by interaction class.

S6.5. Dispersion contributions to the SAPT interaction energy

Figure S22 displays a somewhat inverse relationship between the strength of the interaction and the dispersion contribution, although this is clearly not linear and roughly resembles the boomerang-like shape observed for the relationship between interaction energy and d_{norm} (Figure 6). The distributions indicate that pnictogen bonds tend to have the smallest dispersion contribution, followed by halogen, then chalcogen bonds. Tetrel bond interactions are bimodal in terms of the dispersion contribution, with one maximum close to that of chalcogen bonds, and a second for weaker interactions with a high dispersion contribution.

Plots of the dispersion contributions for complexes classified as electrostatic-dominated and mixed-character are presented in Figures S23 and S24, respectively. The methodology used to classify electrostatic-dominated complexes guarantees that the dispersion contribution to these complexes must be relatively weak,[53] and this is reflected in the scatter plot. There is visually little correlation between the interaction energy and dispersion contribution for this class of complexes, although it is clear that the strongest of the interactions have the smallest dispersion contributions. Turning to the mixed-character complexes, again a distribution resembling a boomerang-like shape is evident, with the stronger interactions also having smaller dispersion contributions. The dispersion-dominated complexes are not shown, but they (unsurprisingly) all have large dispersion contributions and are weak interactions.

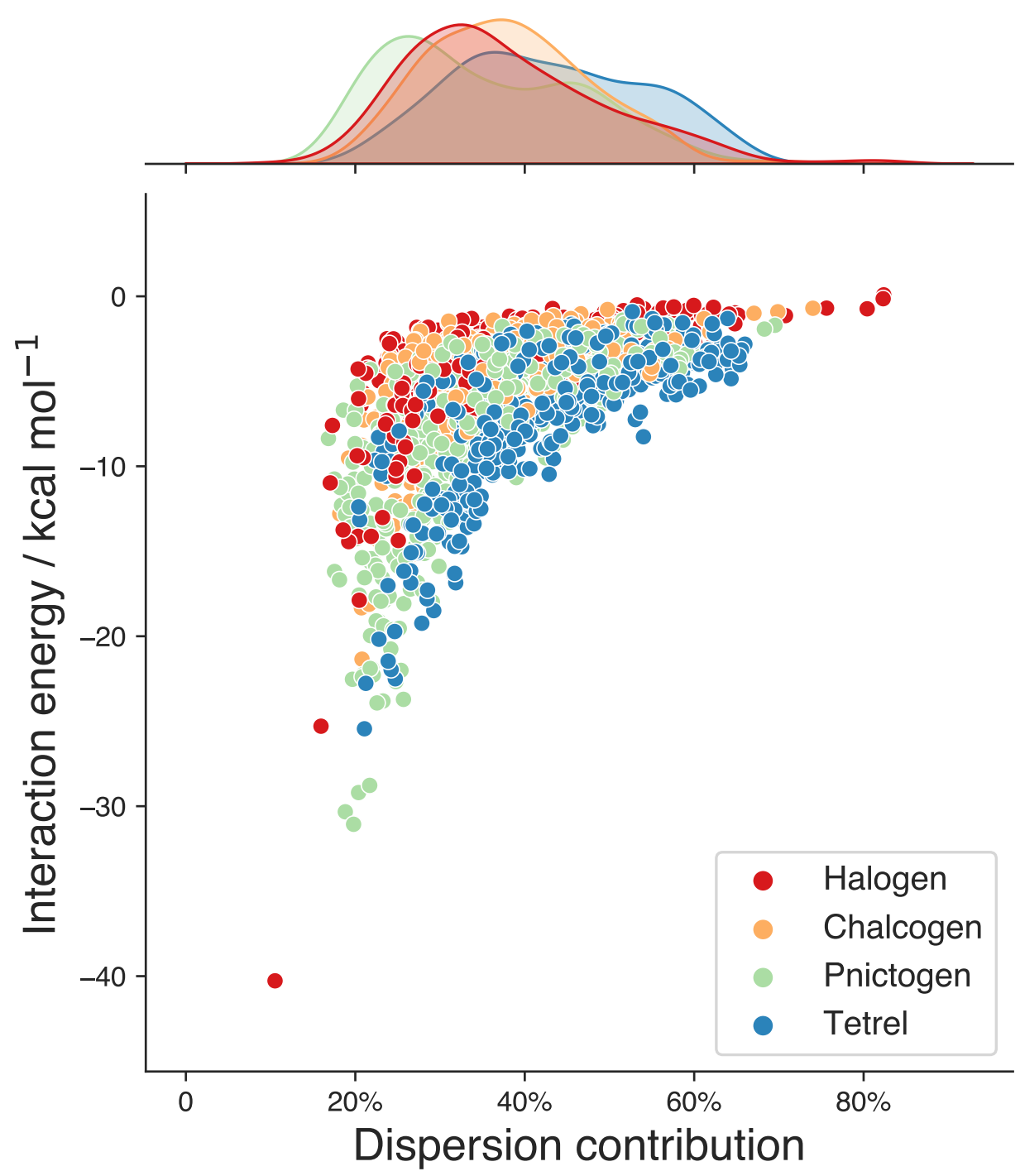

FIG. S22: Counterpoise-corrected LCCSD(T*)-F12 interaction energies plotted against the percentage contribution of the dispersion component to the total of the attractive SAPT terms. Also shown are kernel density estimate plots of the dispersion contributions grouped by interaction class.

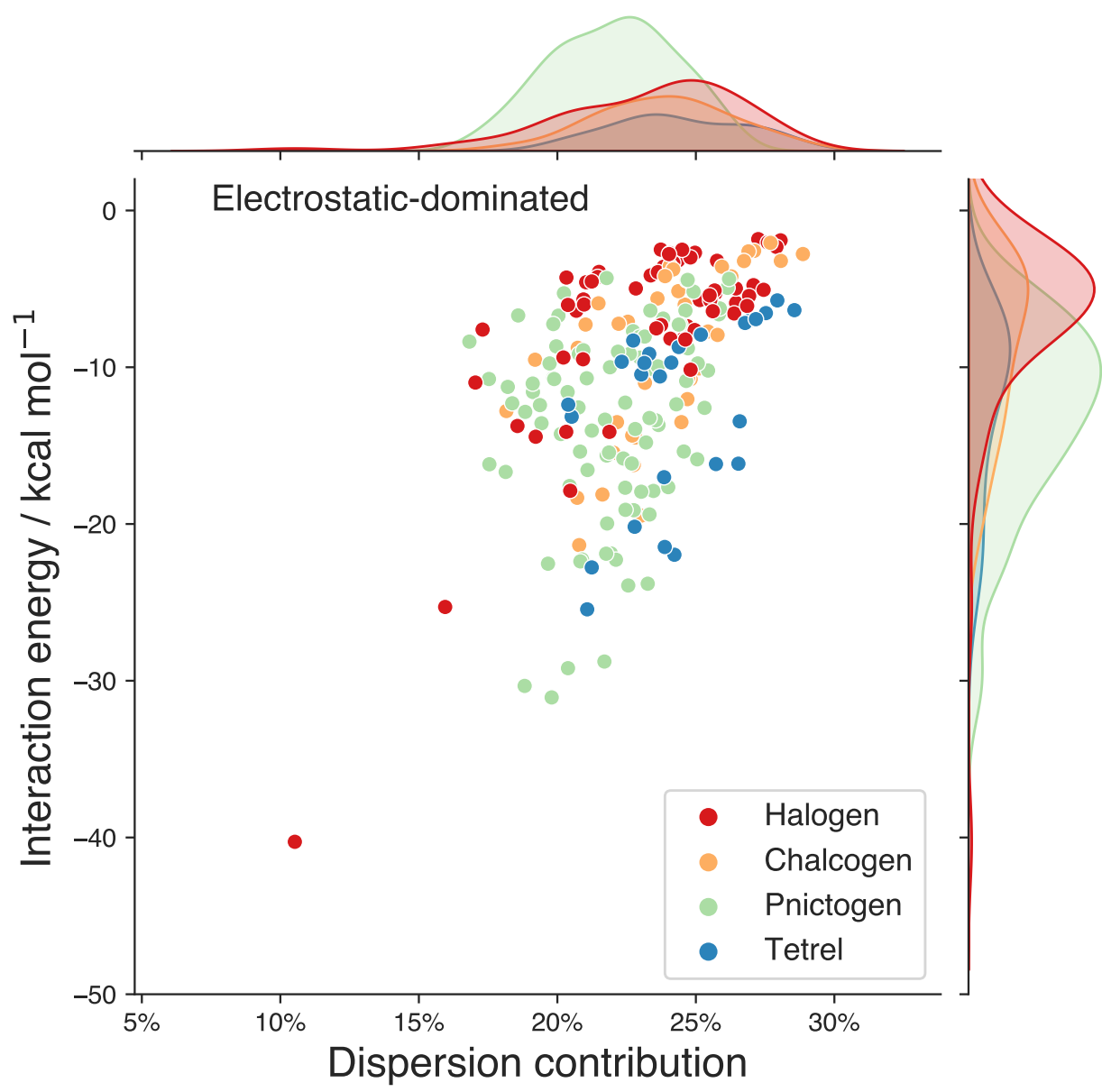

FIG. S23: Counterpoise corrected LCCSD(T*)-F12 interaction energies, for complexes classified as electrostatic-dominated, plotted against the percentage contribution of the dispersion component to the total of the attractive SAPT terms. Also shown are kernel density estimate plots of the dispersion contributions grouped by interaction class.

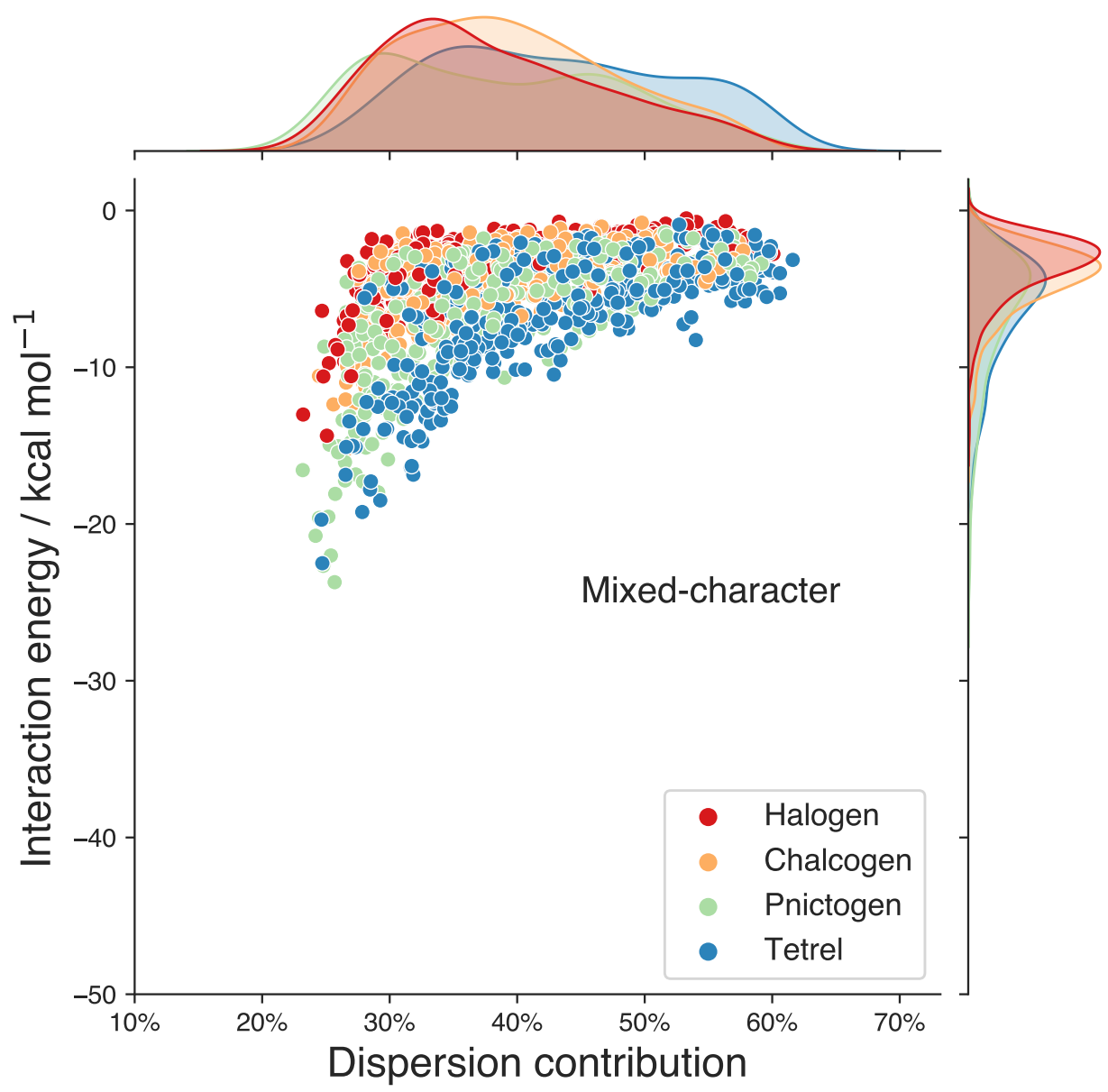

FIG. S24: Counterpoise corrected LCCSD(T*)-F12 interaction energies, for complexes classified as mixed-character, plotted against the percentage contribution of the dispersion component to the total of the attractive SAPT terms. Also shown are kernel density estimate plots of the dispersion contributions grouped by interaction class.

S7. PERFORMANCE OF THE PNO-LCCSD(T*)-F12B METHOD FOR THE SH250 DATASET INTERACTION ENERGIES

Although the PNO-LCCSD(T*)-F12b/aVDZ-F12 level of theory has been shown to produce excellent results for the S66 set of non-covalent interactions (RMSD = 0.05 kcal mol^{-1}),[19–21] less is known about its potential performance for systems that feature σ-hole interactions. Preliminary investigations by Ma and Werner on the X40 set of halogen bond interactions suggest that these interactions may have a larger basis set dependence than for S66, but the same investigation also highlighted reservations about composite CCSD(T)/CBS values that do not use explicit correlation, particularly around error cancellation between CCSD and (T) contributions.[21] To provide a gauge for the potential accuracy of the PNO-LCCSD(T*)-F12b/aVDZ-F12 (abbreviated as aVDZ-F12*) approach for σ-hole interactions, we have calculated the counterpoise-corrected interaction energy for all of the equilibrium structures in the SH250×10 set of σ-hole interactions (SH250 herein) and compared them to the composite CCSD(T)/CBS values.[54] This results in an RMSD of 0.22 kcal mol^{-1}, and a mean absolute deviation (MAD) of 0.19 kcal mol^{-1}.

The distribution of the deviation between aVDZ-F12* and composite CCSD(T)/CBS interaction energies is shown as a histogram in Figure S25, where it can be seen that aVDZ-F12* calculates the interactions to be slightly weaker, in general, than those from the composite CCSD(T)/CBS approach. Figure S26 sub-divides the SH250 set by the σ-hole donor atom, and the resulting box plots show that the interactions where iodine or arsenic are the donor atom tend to have the greatest disagreement with the CCSD(T)/CBS values. Within the aVDZ-F12* results, a total of 16 interaction energies are outside of 1.5 times the interquartile range for each donor atom. From these 'outliers', nine of the complexes involve molecular halogens (I_2, Br_2 or Cl_2) as the σ-hole donor molecule, which are also noted as difficult cases in the article describing the SH250×10 set.[54] There are no molecular halogens in the SHEF1552 set.

Although aVDZ-F12* produces results close to the composite reference at what is likely to be a small fraction of the computational cost, the effect of increasing the basis set size to the equivalent triple-zeta basis set is also of interest, both with (denoted aVTZ-F12*) and without (aVTZ-F12) size consistent scaling of the perturbative triples corrections. All other settings for the PNO-LCCSD(T)-F12b calculations remain the same. To prevent

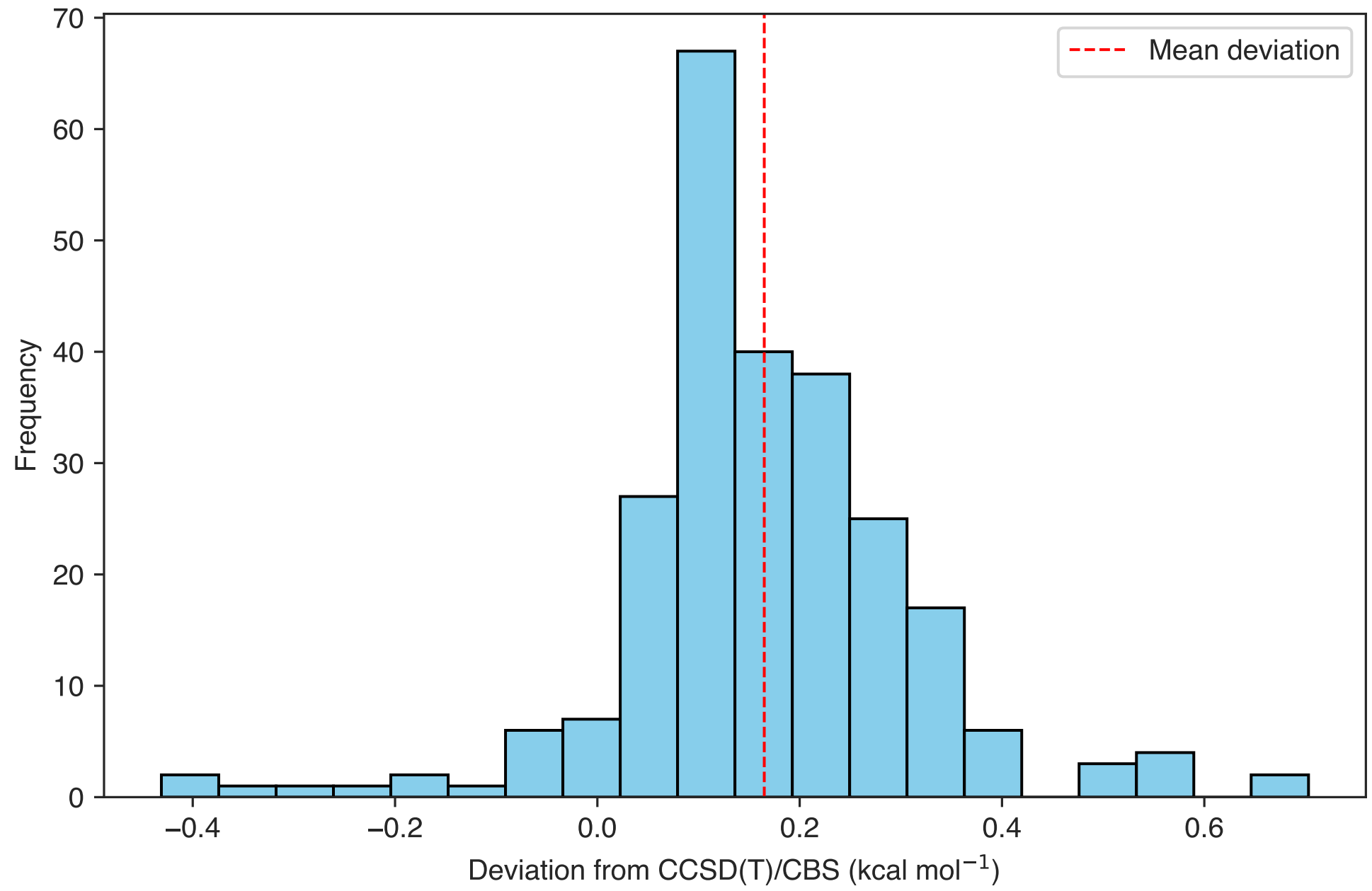

FIG. S25: Distribution of the deviation between PNO-LCCSD(T*)-F12b/aVDZ-F12 and composite CCSD(T)/CBS interaction energies for the SH250 set. Positive values of the deviation indicate that PNO-LCCSD(T*)-F12b/aVDZ-F12 underestimates the strength of the interaction.

the unnecessary computational expense of carrying out triple-zeta calculations on all 250 complexes, we adopt a stratified sampling approach to select a subset of structures for this task. Using the distribution of the deviation from the composite CCSD(T)/CBS interaction energy in Figure S25, we define three deviation 'bins'. The first is between 0 and 0.15 in terms of the quantile of the deviations, the second between 0.4 and 0.6, and a final bin between 0.85 and 1. For each of the seven donor atom types present in the SH250 set, one point is selected from the lowest deviation bin, one from the highest bin, and two from the middle bin, resulting in a total of 28 complexes sampled for further calculations.

For this sampled sub-set, the RMSD when using aVDZ-F12* is 0.23 kcal mol^{-1} and the MAD is 0.20 kcal mol^{-1}, which is very similar to the deviation for the full SH250 set. Increasing the basis set size to aVTZ-F12 produces an RMSD of 0.20 kcal mol^{-1}, and the MAD is reduced by 13% to 0.17 kcal mol^{-1}, representing only small improvements for the associated increase in computational cost. Scaling the (T) contribution to form the aVTZ-F12* level of theory again results in a relatively modest improvement in RMSD (0.16 kcal mol^{-1}), but the MAD does reduce by 56% relative to aVDZ-F12 (0.09 kcal mol^{-1}). The distributions of

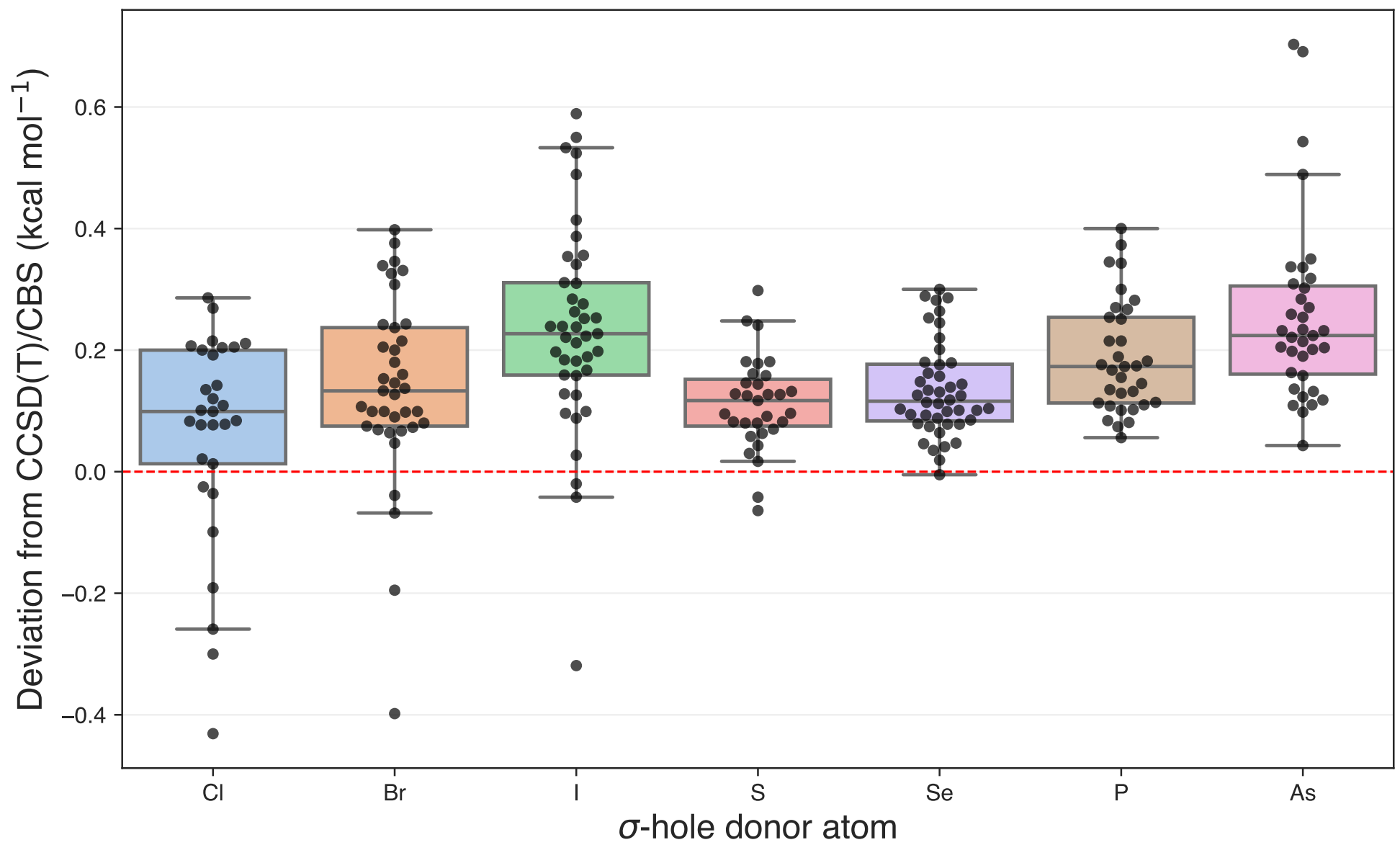

FIG. S26: Box and swarm plots of the deviation between PNO-LCCSD(T*)-F12b/aVDZ-F12 and composite CCSD(T)/CBS interaction energies for the SH250 set. The deviations are classified by the σ-hole donor atom.

the deviations for the sampled sub-set are illustrated as box and swarm plots in Figure S27. Momentarily ignoring the three obvious outliers, there seems to be little reason to choose aVTZ-F12 over aVDZ-F12* as the distributions are similar. The three over-bound outliers for aVDZ-F12* are all complexes involving molecular halogens. We also note that complexes where As or Br are the donor atom tend to perform worse with aVTZ-F12 than with aVDZ-F12*. In general aVTZ-F12* improves the agreement with the composite CCSD(T)/CBS interaction energies, but again there are some substantially over-bound outliers. Both of these interactions also involve molecular halogens. The interaction energies of complexes involving molecular halogens may be worthy of further investigation to determine the source of the disagreement between aVTZ-F12* and the composite CCSD(T)/CBS values. However, as the SHEF1552 set does not include any molecular halogens, it is beyond the scope of the current work. We conclude that the agreement between PNO-LCCSD(T*)-F12b/aVDZ-F12 and the composite CCSD(T)/CBS values is generally impressive given the relatively low computational cost of the former, and that increasing the basis set size to triple-zeta offers limited improvements that do not offset the increased costs.

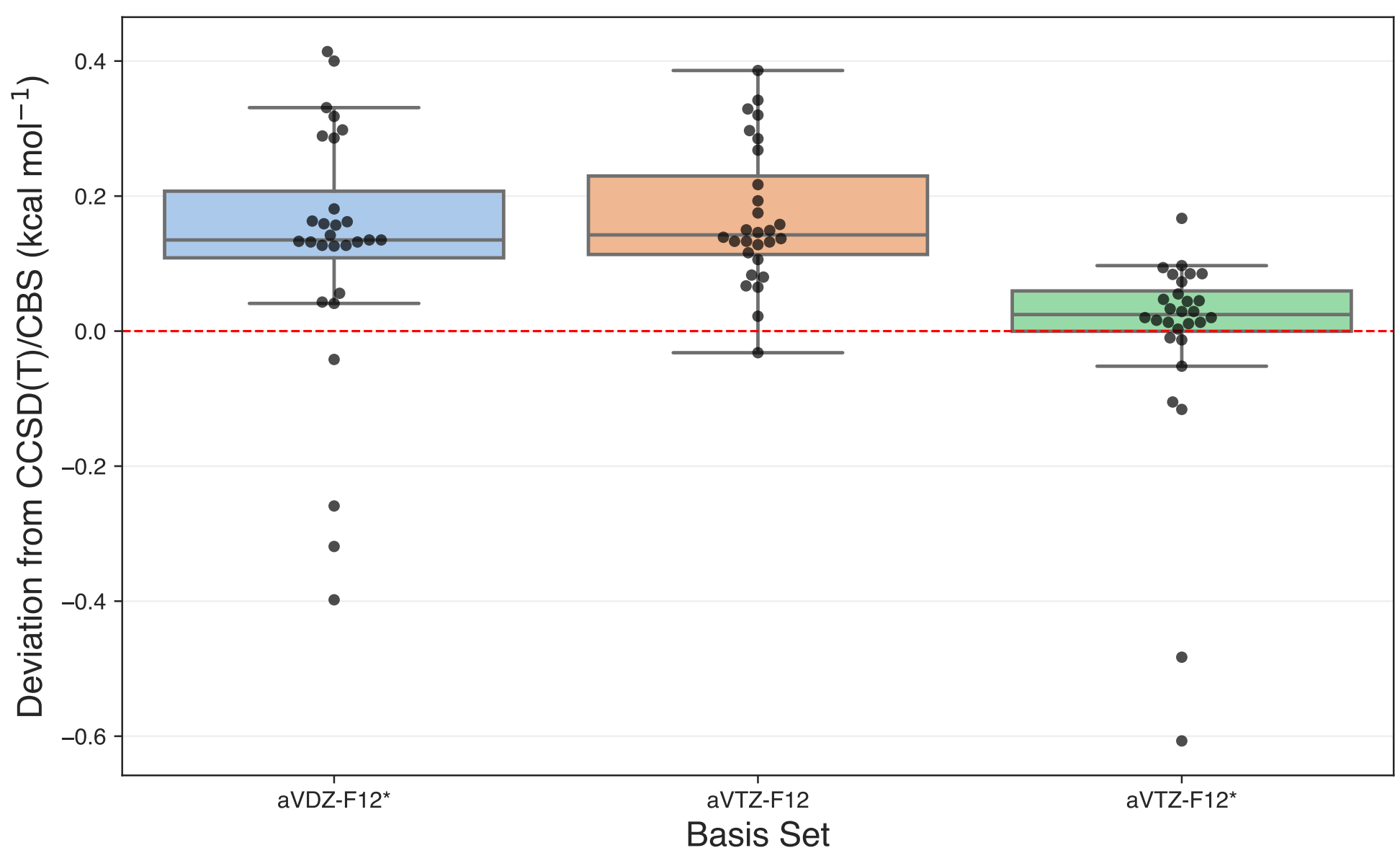

FIG. S27: Box and swarm plots of the deviation between using various basis sets and perturbative triples scaling with the PNO-LCCSD(T)-F12b method, relative to composite CCSD(T)/CBS interaction energies for 28 complexes sampled from the SH250 set.

S8. REFERENCES

[1] F. Weigend and R. Ahlrichs, *Phys. Chem. Chem. Phys.*, 2005, **7**, 3297–3305.

[2] D. Rappoport and F. Furche, *J. Chem. Phys.*, 2010, **133**, 134105.

[3] B. Metz, H. Stoll and M. Dolg, *J. Chem. Phys.*, 2000, **113**, 2563–2569.

[4] K. A. Peterson, D. Figgen, E. Goll, H. Stoll and M. Dolg, *J. Chem. Phys.*, 2003, **119**, 11113–11123.

[5] F. Neese, *Wiley Interdiscip. Rev. Comput. Mol. Sci.*, 2022, **12**, e1606.

[6] F. Weigend, *Phys. Chem. Chem. Phys.*, 2006, **8**, 1057–1065.

[7] `https://github.com/Sheffield-Theoretical-Chemistry/twizzler`, accessed August 19, 2026.

[8] N. M. O'Boyle, A. L. Tenderholt and K. M. Langner, *J. Comp. Chem.*, 2008, **29**, 839–845.

[9] M. Schwilk, Q. Ma, C. Köppl and H.-J. Werner, *J. Chem. Theory Comput.*, 2017, **13**, 3650–3675.

[10] Q. Ma, M. Schwilk, C. Köppl and H.-J. Werner, *J. Chem. Theory Comput.*, 2017, **13**, 4871–4896.

[11] Q. Ma and H.-J. Werner, *J. Chem. Theory Comput.*, 2018, **14**, 198–215.

[12] Q. Ma and H.-J. Werner, *Wiley Interdiscip. Rev. Comput. Mol. Sci.*, 2018, **8**, e1371.

[13] K. A. Peterson, T. B. Adler and H.-J. Werner, *J. Chem. Phys.*, 2008, **128**, 084102.

[14] N. Sylvetsky, M. K. Kesharwani and J. M. L. Martin, *J. Chem. Phys.*, 2017, **147**, 134106.

[15] J. G. Hill and K. A. Peterson, *J. Chem. Phys.*, 2014, **141**, 094106.

[16] J. G. Hill, *J. Chem. Phys.*, 2024, **160**, 114102.

[17] K. A. Peterson, B. C. Shepler, D. Figgen and H. Stoll, *J. Phys. Chem. A*, 2006, **110**, 13877–13883.

[18] B. Metz, M. Schweizer, H. Stoll, M. Dolg and W. Liu, *Theor. Chem. Acc.*, 2000, **104**, 22–28.

[19] J. Řezáč, K. E. Riley and P. Hobza, *J. Chem. Theory Comput.*, 2011, **7**, 2427–2438.

[20] J. Řezáč, K. E. Riley and P. Hobza, *J. Chem. Theory Comput.*, 2011, **7**, 3466–3470.

[21] Q. Ma and H.-J. Werner, *J. Chem. Theory Comput.*, 2019, **15**, 1044–1052.

[22] H.-J. Werner, P. J. Knowles, F. R. Manby, J. A. Black, K. Doll, A. Hesselmann, D. Kats, A. Köhn, T. Korona, D. A. Kreplin, Q. Ma, T. F. Miller III, A. Mitrushenkov, K. A. Peterson, I. Polyak, G. Rauhut and M. Sibaev, *J. Chem. Phys.*, 2020, **152**, 144107.

[23] H.-J. Werner, P. J. Knowles, P. Celani, W. Györffy, A. Hesselmann, D. Kats, G. Knizia, A. Köhn, T. Korona, D. Kreplin, R. Lindh, Q. Ma, F. R. Manby, A. Mitrushenkov, G. Rauhut, M. Schütz, K. R. Shamasundar, T. B. Adler, R. D. Amos, S. J. Bennie, A. Bernhardsson, A. Berning, J. A. Black, P. J. Bygrave, R. Cimiraglia, D. L. Cooper, D. Coughtrie, M. J. O. Deegan, A. J. Dobbyn, K. Doll, M. Dornbach, F. Eckert, S. Erfort, E. Goll, C. Hampel, G. Hetzer, J. G. Hill, M. Hodges, T. Hrenar, G. Jansen, C. Köppl, C. Kollmar, S. J. R. Lee, Y. Liu, A. W. Lloyd, R. A. Mata, A. J. May, B. Mussard, S. J. McNicholas, W. Meyer, T. F. Miller III, M. E. Mura, A. Nicklass, D. P. O'Neill, P. Palmieri, D. Peng, K. A. Peterson, K. Pflüger, R. Pitzer, I. Polyak, M. Reiher, J. O. Richardson, J. B. Robinson, B. Schröder, M. Schwilk, T. Shiozaki, M. Sibaev, H. Stoll, A. J. Stone, R. Tarroni, T. Thorsteinsson, J. Toulouse, M. Wang, M. Welborn and B. Ziegler, *MOLPRO, version 2023.2, a package of ab initio programs*, 2023, `http://www.molpro.net`, see http://www.molpro.net.

[24] F. Weigend, *Phys. Chem. Chem. Phys.*, 2002, **4**, 4285–4291.

[25] H. W. Nash, R. A. Shaw and J. G. Hill, *J. Comput. Chem.*., 2023, **44**, 1119–1128.

[26] F. Weigend, *J. Comput. Chem.*, 2008, **29**, 167–175.

[27] E. F. Valeev, *Chem. Phys. Lett.*, 2004, **395**, 190–195.

[28] R. A. Shaw and J. G. Hill, *J. Chem. Theory Comput.*, 2017, **13**, 1691–1698.

[29] S. Kritikou and J. G. Hill, *J. Chem. Theory Comput.*, 2015, **11**, 5269–5276.

[30] M. Schwilk, D. Usvyat and H.-J. Werner, *J. Chem. Phys.*, 2015, **142**, 121102.

[31] G. Knizia, T. B. Adler and H.-J. Werner, *J. Chem. Phys.*, 2009, **130**, 054104.

[32] O. Marchetti and H.-J. Werner, *J. Phys. Chem. A*, 2009, **113**, 11580–11585.

[33] S. Ten-No, *J. Chem. Phys.*, 2004, **121**, 117–129.

[34] S. Ten-No, *Chem. Phys. Lett.*, 2004, **398**, 56–61.

[35] M. K. Kesharwani, D. Manna, N. Sylvetsky and J. M. L. Martin, *J. Phys. Chem. A*, 2018, **122**, 2184–2197.

[36] N. Mehta and J. M. L. Martin, *Phys. Chem. Chem. Phys.*, 2025, **27**, 1274–1283.

[37] H.-J. Werner, G. Knizia and F. R. Manby, *Mol. Phys.*, 2011, **109**, 407–417.

[38] K. A. Peterson, C. Krause, H. Stoll, J. G. Hill and H.-J. Werner, *Mol. Phys.*, 2011, **109**, 2607–2623.

[39] D. G. A. Smith, L. A. Burns, A. C. Simmonett, R. M. Parrish, M. C. Schieber, R. Galvelis, P. Kraus, H. Kruse, R. Di Remigio, A. Alenaizan, A. M. James, S. Lehtola, J. P. Misiewicz,

M. Scheurer, R. A. Shaw, J. B. Schriber, Y. Xie, Z. L. Glick, D. A. Sirianni, J. S. O'Brien, J. M. Waldrop, A. Kumar, E. G. Hohenstein, B. P. Pritchard, B. R. Brooks, H. F. Schaefer III, A. Y. Sokolov, K. Patkowski, A. E. DePrince III, U. Bozkaya, R. A. King, F. A. Evangelista, J. M. Turney, T. D. Crawford and C. D. Sherrill, *J. Chem. Phys.*, 2020, **152**, 184108.

[40] E. G. Hohenstein and C. D. Sherrill, *J. Chem. Phys.*, 2010, **133**, 014101.

[41] F. Weigend, M. Häser, H. Patzelt and R. Ahlrichs, *Chem. Phys. Lett.*, 1998, **294**, 143–152.

[42] F. Weigend, A. Köhn and C. Hättig, *J. Chem. Phys.*, 2002, **116**, 3175–3183.

[43] A. Hellweg and D. Rappoport, *Phys. Chem. Chem. Phys.*, 2015, **17**, 1010–1017.

[44] C. R. Groom, I. J. Bruno, M. P. Lightfoot and S. C. Ward, *Acta Crystallogr. B*, 2016, **72**, 171–179.

[45] I. J. Bruno, J. C. Cole, P. R. Edgington, M. Kessler, C. F. Macrae, P. McCabe, J. Pearson and R. Taylor, *Acta Crystallogr. B*, 2002, **58**, 389–397.

[46] R. A. Sykes, N. T. Johnson, C. J. Kingsbury, J. Harter, A. G. P. Maloney, I. J. Sugden, S. C. Ward, I. J. Bruno, S. A. Adcock, P. A. Wood, P. McCabe, A. A. Moldovan, F. Atkinson, I. Giangreco and J. C. Cole, *J. Appl. Cryst.*, 2024, **57**, 1235–1250.

[47] D. M. Gil, J. Echeverría and S. Alvarez, *Inorg. Chem.*, 2022, **61**, 9082–9095.

[48] J. Echeverría and S. Alvarez, *Chem. Sci.*, 2023, **14**, 11647–11688.

[49] A. C. Legon, *Phys. Chem. Chem. Phys.*, 2014, **16**, 12415–12421.

[50] A. C. Legon, *Phys. Chem. Chem. Phys.*, 2014, **16**, 25199–25199.

[51] R. A. Shaw and J. G. Hill, *Inorganics*, 2019, **7**, 19.

[52] E. Engelage, D. Reinhard and S. M. Huber, *Chem. Eur. J.*, 2020, **26**, 3843–3861.

[53] L. Gráfová, M. Pitoňák, J. Řezáč and P. Hobza, *J. Chem. Theory Comput.*, 2010, **6**, 2365–2376.

[54] K. Kříž and J. Řezáč, *Phys. Chem. Chem. Phys.*, 2022, **24**, 14794–14804.
